# A meta-analysis of the reproducibility of food frequency questionnaires in nutritional epidemiological studies

**DOI:** 10.1186/s12966-020-01078-4

**Published:** 2021-01-11

**Authors:** Qi Cui, Yang Xia, Qijun Wu, Qing Chang, Kaijun Niu, Yuhong Zhao

**Affiliations:** 1 Present address: Department of Clinical Epidemiology, Shengjing Hospital of China Medical University, Shenyang, China; 2 Present address: Nutritional Epidemiology Institute and School of Public Health, Tianjin Medical University, Tianjin, China

**Keywords:** FFQ, Food frequency questionnaire, Reproducibility, Meta-analysis, Macronutrients, micronutrients

## Abstract

**Background:**

Reproducibility of FFQs measures the consistency of the same subject at different time points. We performed a meta-analysis to explore the reproducibility of FFQs and factors related to reproducibility of FFQs.

**Methods and findings:**

A systematic literature review was performed before July 2020 using PubMed and Web of Science databases. Pooled intraclass and Spearman correlation coefficients (95% confidence interval) were calculated to assess the reproducibility of FFQs. Subgroup analyses based on characteristics of study populations, FFQs, or study design were performed to investigate factors related to the reproducibility of FFQs. A total of 123 studies comprising 20,542 participants were eligible for the meta-analysis. The pooled crude intraclass correlation coefficients ranged from 0.499 to 0.803 and 0.499 to 0.723 for macronutrients and micronutrients, respectively. Energy-adjusted intraclass correlation coefficients ranged from 0.420 to 0.803 and 0.507 to 0.712 for macronutrients and micronutrients, respectively. The pooled crude and energy-adjusted Spearman correlation coefficients ranged from 0.548 to 0.851 and 0.441 to 0.793, respectively, for macronutrients; and from 0.573 to 0.828 and 0.510 to 0.744, respectively, for micronutrients. FFQs with more food items, 12 months as dietary recall interval (compared to less than 12 months), and a shorter time period between repeated FFQs resulted in superior FFQ reproducibility.

**Conclusions:**

In conclusion, FFQs with correlation coefficients greater than 0.5 for most nutrients may be considered a reliable tool to measure dietary intake. To develop FFQs with higher reproducibility, the number of food items and dietary recall interval should be taken into consideration.

**Supplementary Information:**

The online version contains supplementary material available at 10.1186/s12966-020-01078-4.

## Introduction

The FFQ is the most commonly used tool to assess individual usual dietary intake in nutritional epidemiological studies, especially for investigating the relationship between dietary and health outcomes [1, 2]. FFQs allow researchers to rank subjects according to their dietary and nutritional intake. Obtaining an accurate estimate of long-term habitual food intake is crucial [3], which is very important to better understand diet and associated diseases. However, assessment of nutritional habits is complex [4], and they are affected by real changes in regular dietary intake and random changes in FFQ [5, 6]. FFQs allow covering a wider range of foods, including those consumed rarely, and can be administered once whereas to describe usual dietary habits with a reasonable reproducibility [7]. If the reproducibility is not maintained high enough, the dietary intakes of subjects measured at baseline would substantially misclassify their true exposure during the study period [8]. To enhance the interpretation of estimated diet–disease associations and to improve the translation of such associations into dietary recommendations, reproducibility analysis is required before applying FFQ to analyze dietary intake [9].

Reproducibility reflects reliability and refers to the similarity of the same method at different timepoints [10]. Reproducibility is generally assessed by administering the same FFQ twice to the same group of subjects and analyzing the association between the two responses [11]. Previous studies reported that the intervals between two FFQs varied from 1 week [12] to 2 years [13]. And true change in regular dietary intakes and random variation in response to the FFQ have been considered factors affecting the repeatability of FFQs [14], which result in reduced reproducibility of FFQs with long interval [2, 15]. However, the two FFQs administered closely, respondents may remember and repeat their previous responses and result in high reproducibility [2].

Numerous studies have been devoted to assess the reproducibility of FFQs before applying FFQ to different populations. The Spearman and intraclass correlation coefficients (ICCs) to assess the reproducibility of 134-item FFQs with approximately 6 months apart ranged from 0.46 to 0.79 and from 0.34 to 0.71, respectively, for 25 nutrients in the Shanghai Diet and Health Study [16]. The reproducibility of another FFQ of 157 items with 3-month interval used in the Food4Me study (a randomized controlled trial across seven European countries) has been reported to range from 0.62 to 0.89 [17]. Then, a repeatability study of an interview administered FFQ of 135 items in the Mexican Women’s Bone Health Cohort Study found that the reproducibility coefficients range from 0.186 to 0.810 for energy-unadjusted data and 0.174 to 0.597 for energy-adjusted data [18]. However, the correlation coefficients of different nutrients evaluated in different studies are different, and a widely accepted reference value for the reproducibility of FFQs is currently lacking.

Furthermore, the characteristics of FFQs may affect their reproducibility. A previous study reported that the ICCs of an FFQ comprising 255 items ranged from 0.69 (fat) to 0.84 (vitamin A) in Moroccan adults [19]. A shorter FFQ assessing the average consumption of 57 food items was reported to have a reliability coefficient ranging from 0.56 to 0.70 [20]. Therefore, FFQ items may induce differences in reproducibility. A previous study suggested that the median (range) energy-adjusted Spearman correlation coefficients (SCCs) for 30 nutrients between two FFQ measurements was 0.24 (0.04–0.69) for men and 0.50 (0.27–0.60) for women [21], suggesting that the reliability of FFQs differ between men and women. Moreover, differences in FFQ reproducibility may be caused by other factors [22], such as real changes in diet over time, individual differences in diet, and study design differences [22, 23]. However, there has been a paucity of studies comprehensively exploring the effects of these factors on the reproducibility of FFQs.

Although the reproducibility of FFQs has been evaluated in various studies, there has yet to be a comprehensive meta-analysis of the reproducibility studies and definition of reference ranges for reproducibility coefficients. Moreover, no study has systematically explored the factors related to the reproducibility of FFQs. Therefore, we conducted a meta-analysis to systematically assess the reproducibility of FFQs and to explore the factors related to the reproducibility of FFQs.

## Methods

A systematic review was conducted according to the Preferred Reporting Items for Systematic Reviews and Meta-Analyses (PRISMA) guideline; the relevant checklist is provided in PRISMA Checklist.

### Literature search

We conducted a comprehensive literature search for published studies from PubMed and Web of Science databases before July 2020. The literature search was conducted by two independent researchers. The search strategy used employed the terms “FFQ OR food frequency questionnaire” AND “reproducibility OR repeatability OR reliability”.

### Study identification and selection

The potentially relevant articles were evaluated by two independent reviewers based on the inclusion. The original studies were obtained from the database. After removing duplicates, we screened the studies according to title and abstract. After reading the full texts, the eligible articles were obtained by exclusion criteria.

Articles were included if they met the following criteria: (1) FFQs were used to measure nutrient intake; (2) the age range of target healthy populations was between 8 and 86 years; (3) the study assessed the reproducibility of FFQs; (4) the study was published in English; and (5) the reproducibility of FFQs was measured with the intraclass correlation coefficient (ICC) and Pearson correlation coefficient or SCC.

The exclusion criteria were: (1) food intake was assessed using FFQs; (2) FFQs were used to assess a specific nutrient; (3) the target population was unhealthy people or specific populations, such as individuals who were overweight or malnourished; (4) the participants were less than 8 years old; (5) the article investigated diet-disease relationships; and (6) the full text was unavailable through web searches.

### Data extraction

Data were extracted from each study by independent reviewers. The extracted contents included the following, excluding authors and published years: (1) characteristics of participants including sample size, age, gender distribution, and region; (2) characteristics of FFQs including food items and dietary recall interval; (3) characteristics of study design including administration method and interval between two FFQs; and (4) statistics employed to assess reproducibility between repeated FFQs including ICC and Pearson correlation coefficient or SCC in relation to energy, macronutrients, and micronutrients (minerals and vitamins). Macronutrients included protein, fat, plant fat, animal fat, MUFA, PUFA, n-3 PUFA, n-6 PUFA, SFA, linoleic acid, linolenic acid, EPA, DHA, trans-fat, cholesterol, lipid, carbohydrate, sucrose, sugar, starch, fiber, soluble fiber, insoluble fiber, and alcohol. Minerals included selenium (Se), magnesium (Mg), calcium (Ca), iron (Fe), iodine (I); zinc (Zn), copper (Cu), potassium (K), phosphorus (P), sodium (Na), and manganese (Mn). Vitamins included vitamin A, retinol, carotene, β-carotene, vitamin C, vitamin D, vitamin E, vitamin K, thiamin, riboflavin, niacin, vitamin B6, folate and vitamin B12.

### Meta-analysis

The pooled correlation coefficients were calculated based on the ICC and SCC values obtained from each article. We converted Pearson correlation coefficients into SCCs if the latter were lacking. Fisher’s transformation was used to convert each correlation coefficient to an approximately normally distributed z-value. The standard error of z was calculated. After appropriate conversion, random effects meta-analyses were used to combine data. The heterogeneity of the z-values among studies was determined by calculating the inconsistency index (I^2^). I^2^ greater than 50% indicated the presence of heterogeneity. z-values were converted using inverse Fisher’s transformation to obtain correlation coefficients and 95% CIs to account for results. Sensitivity analysis was performed to explore the when to further explore the source of heterogeneity.

Studies were stratified according to the following characteristics: (1) population characteristics including age (< 18 years, 18–50 years, and > 50 years), gender, and region; (2) characteristics of the reproducibility studies including sample size (≤ 112 and > 112 , the cutoff point was the median of sample size) and time interval between repeated FFQs (≤6 months and > 6 months, the cutoff point was the median of time interval); and (3) characteristics of FFQ design including FFQ items (≤ 120 and > 120, the cutoff point was the median of item), dietary recall interval (≥12 months and < 12 months), administration mode (interviewer-administered or self-administered). All statistical analyses were performed using Stata Software (Version 11.0 Stata, College Station, TX, USA). A *P*-value less than 0.05 was considered statistically significant.

## Results

### Literature search and study selection

**Fig. 1:**
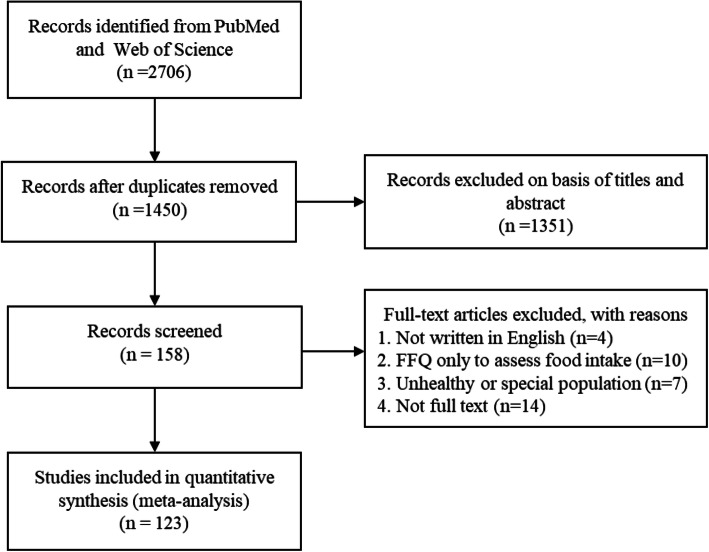
Flowchart of the study selection process

The flow chart of the study selection is shown in Fig. 1. We identified 2706 original studies from the database. After removing 1256 duplicates, 159 articles met the inclusion criteria according to title and abstract screening. After reading the full texts, 35 articles were excluded according to exclusion criteria. In total, we obtained 123 articles based on the procedure described above.

**Table 1:** Summary of the characteristics of the included studies ^a^

	Overall	Adult	Old	Adolescent
Range of age (years)	8–86	18–50	> 50	8–17
Number of studies	124	78	33	15
**Population characteristics**				
Total number	20,830	11,336	7897	1597
Sample size	112 (14–1981)	102 (20–1623)	158 (14–1981)	101 (48–185)
Distribution of gender				
Region				
Africa	6 (4.88)	5 (6.49)	0 (0)	1 (6.67)
Oceania	8 (6.50)	5 (6.49)	2 (6.07)	1 (6.67)
Asia	37 (30.08)	22 (28.57)	11 (33.33)	4 (26.67)
Europe	35 (28.46)	22 (28.57)	10 (30.30)	4 (26.67)
America	37 (30.08)	23 (29.87)	10 (30.30)	5 (33.33)
**FFQ design characteristics**				
Items of FFQ				
≥ 120	61 (49.59)	41 (53.25)	15 (45.45)	5 (33.33)
< 120	62 (50.41)	36 (46.75)	18 (54.55)	10 (66.67)
Dietary recall intervals				
≥ Previous 12 months	80 (65.04)	50 (64.94)	28 (84.85)	4 (26.67)
< Previous 12 months	33 (26.83)	20 (25.97)	3 (9.09)	10 (66.67)
Not available	10 (8.13)	7 (9.09)	2 (6.06)	1 (6.667)
**Characteristics of the reproducibility studies**				
Administration mode of FFQ				
Interview-administered	44 (35.77)	29 (37.66)	12 (36.36)	4 (26.67)
Self-administered	63 (51.22)	36 (46.75)	19 (57.58)	9 (60.00)
Not available	16 (13.01)	12 (15.59)	2 (6.06)	2 (13.33)
Intervals between FFQs				
< 6 months	63 (51.22)	45 (58.44)	8 (24.24)	12 (80.00)
≥ 6 months	55 (44.72)	29 (37.66)	23 (69.70)	3 (20,00)
Both intervals	5 (4.06)	3 (3.90)	2 (6.06)	0 (0)

^a^Values are N (%) or median (range)

### Study characteristics

An overview of the retrieved studies assessing the reproducibility of FFQs is presented in Table 1 (detail information shown in Supplemental Table 1). Of the 123 articles included [4, 10–13, 15–18, 20, 21, 23–134], two articles analyzed differences in different age groups [50, 113], and five articles assessed the differences in reproducibility according to time intervals between repeated FFQs [39, 91, 96, 113, 134]. The extracted information on characteristics of the included studies is summarized in Table 1 (detail information shown in Supplemental Table 1). The median sample size per study was 112 (range: 14–1981), with a total of 20,542 participants. The age range of participants was between 8 and 86 years. The studies were divided into three groups according to age: adult (18–50 years), elderly (> 50 years) and adolescent (< 18 years); these comprised 77, 33, and 15 studies, respectively. For studies with a wide participant age range covering cutoff point, the mean age reported in articles was used as the grouping criterion first. In addition, the median age was used to group population if the mean age was not available. For FFQ characteristics, the median number of FFQ items was 120. The number of studies that required participants to recall food intake for more or less than 12 months was 80 and 33, respectively. Of these studies, 44 were interview-administered, 63 were self-administered, and 16 were not available. Time intervals between FFQs varied considerably (from 1 week to 2.7 years), and studies were classified as less than 6 months (*n* = 63) or more than 6 months (*n* = 55).

### Correlation coefficients for energy and macronutrients

As shown in Table 2, crude ICCs for reproducibility ranged from 0.499 for starch to 0.803 for alcohol (median: 0.667). All values for energy and macronutrients exceeded 0.5. After adjusting for energy, the range of ICC was between 0.420 (n-3 PUFA) and 0.803 (alcohol) with a median value of 0.630. Energy-adjusted ICCs of most nutrients exceeded 0.5 except those for n-3 PUFA, trans-fat, and soluble fiber. For SCCs, all pooled crude values ranged from 0.548 (plant fat) to 0.851 (alcohol) with a median value of 0.637, and energy-adjusted values ranged from 0.441 (n-6 PUFA) to 0.793 (alcohol) with a median value of 0.580. Most values were decreased after adjusting for energy, except those for lipid and plant fat. All pooled crude SCCs exceeded 0.5; energy-adjusted values exceeded 0.5 except those for n-3 PUFA and n-6 PUFA. Heterogeneity was high for energy and most nutrients in crude and energy-adjusted ICCs and SCCs (I^2^ > 50%).

**Table 2:** Pooled effect estimates and heterogeneity of the correlation coefficients for the reproducibility of FFQ for energy and macronutrient^a^

Nutrient	ICC						SCC					
	Crude			Energy-adjusted			Crude			Energy-adjusted		
	N	ICC (95% CI)	*I*^*2*^	N	ICC (95% CI)	*I*^*2*^	N	ICC (95% CI)	*I*^*2*^	N	ICC (95% CI)	*I*^*2*^
Energy	61	0.709 (0.652, 0.758)	96.3	N/A	N/A	N/A	106	0.649 (0.624, 0.673)	84.6	N/A	N/A	N/A
Protein	63	0.648 (0.612, 0.682)	88.3	25	0.600 (0.546, 0.650)	80.4	106	0.609 (0.584, 0.632)	80.6	64	0.558 (0.521, 0.593)	80.5
Fat	55	0.644 (0.599, 0.684)	91.1	19	0.564 (0.483, 0.634)	86.3	104	0.623 (0.599, 0.644)	78.4	56	0.555 (0.516, 0.593)	81.3
Plant fat	5	0.572 (0.461, 0.665)	60.0	2	0.615 (0.489, 0.715)	0	6	0.548 (0.468, 0.619)	62.0	2	0.580 (0.214, 0.803)	90.6
Animal fat	2	0.696 (0.462, 0.839)	74.7	2	0.725 (0.585, 0.822)	46.4	4	0.693 (0.661, 0.722)	0	2	0.575 (0.513, 0.630)	0
MUFA	41	0.641 (0.603, 0.675)	80.9	18	0.630 (0.557, 0.693)	83.7	50	0.612 (0.583, 0.639)	68.0	31	0.551 (0.495, 0.602)	81.3
PUFA	45	0.641 (0.575, 0.699)	94.6	45	0.573 (0.488, 0.646)	85.5	57	0.595 (0.566, 0.623)	73.4	31	0.521 (0.466, 0.572)	79.4
n-3 PUFA	2	0.703 (0.657, 0.745)	63.1	1	0.420 (0.187, 0.607)	N/A	6	0.619 (0.573, 0.661)	59.8	5	0.469 (0.402, 0.532)	36.9
n-6 PUFA	2	0.727 (0.684, 0.764)	61.3	1	0.510 (0.295, 0.675)	N/A	6	0.594 (0.563, 0.624)	18.3	5	0.441 (0.355, 0.519)	57.4
SFA	49	0.687 (0.612, 0.749)	96.6	19	0.639 (0.564, 0.704)	86.4	65	0.626 (0.598, 0.651)	76.6	37	0.567 (0.520, 0.610)	81.6
Linoleic acid	5	0.666 (0.491, 0.790)	93.3	3	0.685 (0.591, 0.760)	71.0	9	0.615 (0.552, 0.670)	80.6	9	0.577 (0.487, 0.653)	86.3
Linolenic acid	4	0.658 (0.498, 0.775)	88.9	2	0.630 (0.527, 0.714)	47.8	3	0.684 (0.576, 0.769)	86.3	4	0.642 (0.486, 0.759)	91.0
EPA	2	0.600 (0.511, 0.676)	0	N/A	N/A	N/A	3	0.785 (0.579, 0.896)	87	2	0.726 (0.349, 0.901)	87.4
DHA	2	0.611 (0.525, 0.686)	0	N/A	N/A	N/A	3	0.749 (0.616, 0.840)	67.4	N/A	N/A	N/A
Trans-fat	4	0.604 (0.453, 0.721)	79.3	2	0.479 (0.385, 0.562)	0	6	0.615 (0.419, 0.756)	91.0	2	0.586 (−0.07, 0.889)	93.8
Cholesterol	48	0.657 (0.610, 0.699)	90.5	25	0.618 (0.560, 0.670)	82.1	66	0.614 (0.584, 0.642)	79.5	37	0.556 (0.507, 0.603)	82.6
Lipid	4	0.701 (0.459, 0.846)	93.4	4	0.662 (0.370, 0.835)	94.5	6	0.551 (0.495, 0.603)	0	4	0.567 (0.327, 0.738)	83.7
Carbohydrate	64	0.680 (0.616, 0.735)	96.7	23	0.641 (0.566, 0.704)	90.1	101	0.637 (0.609, 0.663)	86.3	60	0.586 (0.547, 0.622)	83.5
Sucrose	4	0.633 (0.533, 0.715)	73.1	1	0.679 (0.607, 0.741)	N/A	7	0.709 (0.644, 0.764)	67.4	1	0.632 (0.513, 0.726)	N/A
Sugar	8	0.707 (0.618, 0.777)	81.4	1	0.779 (0.747, 0.808)	N/A	11	0.689 (0.626, 0.743)	79.2	5	0.639 (0.516, 0.737)	88.6
Starch	2	0.510 (0.264, 0.693)	85.5	N/A	N/A	N/A	4	0.641 (0.604, 0.675)	0	2	0.606 (0.553, 0.654)	0
Fiber	54	0.683 (0.638, 0.723)	92.0	21	0.670 (0.595, 0.733)	91.1	87	0.639 (0.609, 0.667)	84.5	53	0.621 (0.581, 0.658)	83.3
Soluble fiber	2	0.780 (0.679, 0.852)	94.8	1	0.499 (0.340, 0.630)	N/A	14	0.658 (0.603, 0.706)	78.8	10	0.590 (0.493, 0.672)	77.0
Insoluble fiber	2	0.784 (0.684, 0.855)	94.9	2	0.690 (0.616, 0.752)	84.6	12	0.649 (0.595, 0.698)	54.9	12	0.603 (0.527, 0.670)	74.1
Alcohol	22	0.803 (0.749, 0.847)	90.1	9	0.803 (0.725, 0.860)	88.7	47	0.851 (0.821, 0.877)	93.5	27	0.793 (0.746, 0.831)	91.0

^a^*CI* confidence interval, *I*^*2*^ inconsistency index, *N/A* not available

### Correlation coefficients for micronutrients

Table 3 depicts the reproducibility of the FFQ measurements in terms of pooled ICCs and SCCs for micronutrients. For vitamins, the pooled crude and energy-adjusted ICCs varied from 0.589 (retinol) to 0.723 (vitamin B6) and from 0.512 (carotene) to 0.712 (vitamin B6), respectively; values generally exceeded 0.5. The median crude SCC was 0.613 with a range from 0.573 (retinol) to 0.643 (niacin). The median energy-adjusted SCC was 0.38 with a range from 0.510 (carotene) to 0.658 (vitamin K). For mineral intake, the crude and energy-adjusted ICCs ranged from 0.499 to 0.674 (median: 0.640) and from 0.507 to 0.690 (median: 0.626), respectively; the crude and energy-adjusted SCCs ranged from 0.613 to 0.828 (median: 0.637) and from 0.552 to 0.744 (median: 0.597), respectively. The heterogeneity of correlation coefficients for most micronutrients was high (I^2^ > 75%).

**Table 3:** Pooled effect estimates and heterogeneity of the correlation coefficients for the reproducibility of FFQ for micronutrient*

Nutrient	ICC						SCC					
	Crude			Energy-adjusted			Crude			Energy-adjusted		
	N	ICC (95% CI)	*I*^*2*^	N	ICC (95% CI)	*I*^*2*^	N	ICC (95% CI)	*I*^*2*^	N	ICC (95% CI)	*I*^*2*^
Vitamin A	27	0.623 (0.544, 0.692)	95.2	12	0.597 (0.464, 0.705)	92.2	42	0.613 (0.570, 0.651)	87.2	22	0.553 (0.470, 0.627)	89.8
Retinol	18	0.589 (0.513, 0.656)	85.3	9	0.537 (0.421, 0.635)	74	49	0.573 (0.537, 0.607)	80.6	38	0.513 (0.460, 0.562)	84.3
Carotene	9	0.632 (0.499, 0.735)	97.1	5	0.512 (0.328, 0.658)	0.86	25	0.605 (0.558, 0.649)	89	21	0.510 (0.427, 0.584)	90.8
β-Carotene	21	0.677 (0.630, 0.719)	76.1	6	0.613 (0.456, 0.733)	81.9	39	0.613 (0.573, 0.649)	72.3	28	0.554 (0.513, 0.593)	56.5
Vitamin C	47	0.665 (0.600, 0.722)	96.1	22	0.635 (0.526, 0.723)	94.9	92	0.623 (0.594, 0.650)	85.3	57	0.596 (0.555, 0.633)	83.6
Vitamin D	16	0.678 (0.546, 0.777)	98.4	5	0.671 (0.391, 0.837)	98.1	30	0.617 (0.572, 0.659)	83.5	15	0.560 (0.475, 0.635)	81.5
Vitamin E	34	0.665 (0.576, 0.738)	97.5	15	0.606 (0.484, 0.704)	94.4	52	0.626 (0.583, 0.667)	91.4	30	0.555 (0.490, 0.613)	87
Vitamin K	3	0.656 (0.430, 0.804)	97.4	2	0.693 (0.652, 0.729)	0	7	0.602 (0.511, 0.679)	60.4	5	0.658 (0.553, 0.742)	32.7
Thiamin	31	0.630 (0.587, 0.670)	87.3	12	0.606 (0.492, 0.699)	93.1	55	0.606 (0.579, 0.633)	74.3	39	0.522 (0.475, 0.566)	79.5
Riboflavin	28	0.667 (0.616, 0.712)	91.7	10	0.619 (0.483, 0.726)	94.7	54	0.640 (0.611, 0.667)	81	35	0.581 (0.528, 0.628)	85.4
Niacin	22	0.667 (0.609, 0.718)	89.4	10	0.605 (0.499, 0.693)	90.7	39	0.643 (0.573, 0.704)	94.3	34	0.517 (0.452, 0.576)	86.2
Vitamin B6	13	0.723 (0.522, 0.847)	98.4	5	0.712 (0.516, 0.838)	96.6	27	0.610 (0.553, 0.662)	78.8	19	0.555 (0.483, 0.619)	77.2
Folate	25	0.637 (0.582, 0.686)	90.5	6	0.597 (0.495, 0.684)	76.4	49	0.612 (0.577, 0.646)	81.6	26	0.605 (0.544, 0.659)	82.5
Vitamin B12	13	0.678 (0.507, 0.797)	97.7	7	0.683 (0.496, 0.809)	96.8	28	0.635 (0.577, 0.686)	82.3	21	0.575 (0.490, 0.648)	87.5
Se	11	0.661 (0.608, 0.709)	69.3	4	0.586 (0.429, 0.709)	78.7	15	0.648 (0.586, 0.702)	82.4	11	0.568 (0.446, 0.670)	87.6
Mg	19	0.674 (0.612, 0.728)	88.4	6	0.617 (0.492, 0.717)	87.5	30	0.669 (0.603, 0.725)	89.9	19	0.629 (0.544, 0.701)	86.8
Ca	52	0.635 (0.588, 0.676)	91.8	23	0.642 (0.566, 0.708)	91	87	0.622 (0.594, 0.649)	83	55	0.586 (0.545, 0.626)	84.1
Fe	39	0.640 (0.581, 0.692)	93.9	19	0.564 (0.496, 0.625)	79.4	75	0.613 (0.582, 0.642)	83.8	47	0.570 (0.525, 0.612)	82.8
I	2	0.499 (0.338, 0.632)	73.7	3	0.507 (0.421, 0.585)	35	2	0.828 (0.724, 0.894)	19.8	1	0.744 (0.600, 0.841)	N/A
Zn	26	0.595 (0.556, 0.631)	68.8	12	0.573 (0.495, 0.641)	72.3	26	0.623 (0.565, 0.675)	85.3	18	0.597 (0.507, 0.675)	86.7
Cu	4	0.658 (0.620, 0.693)	0	1	0.690 (0.646, 0.728)	N/A	6	0.748 (0.620, 0.837)	86.6	5	0.726 (0.608, 0.813)	86.6
K	25	0.672 (0.598, 0.736)	95.4	7	0.637 (0.486, 0.752)	93.6	49	0.637 (0.605, 0.667)	80	34	0.608 (0.566, 0.647)	73.4
P	23	0.605 (0.521, 0.676)	91.9	9	0.635 (0.544, 0.711)	80.7	43	0.621 (0.575, 0.662)	83.4	30	0.579 (0.521, 0.630)	82.1
Na	25	0.652 (0.499, 0.766)	98.2	8	0.670 (0.474, 0.802)	97	41	0.623 (0.582, 0.661)	83.9	30	0.552 (0.489, 0.609)	86.7
Mn	2	0.621 (0.382, 0.781)	64.8	N/A	N/A	N/A	5	0.655 (0.596, 0.707)	0	2	0.719 (0.645, 0.779)	0

^a^*CI* confidence interval, *I*^*2*^ inconsistency index, *N/A* not available

### Subgroup analysis according to age and sex

**Fig. 2:**
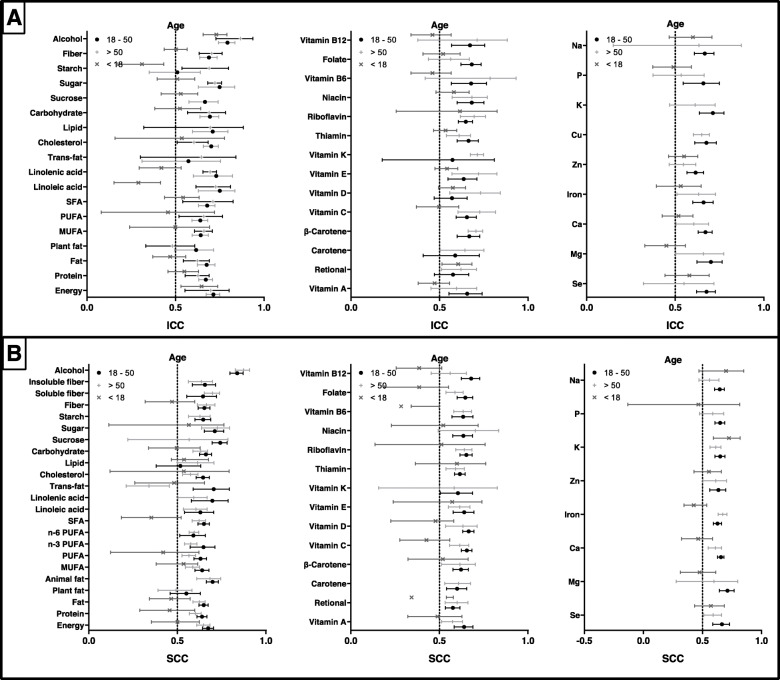
Reproducibility of food frequency questionnaires (FFQ) stratified by age. Values represent pooled intraclass correlation coefficient (ICC) and spearman correlation coefficient (SCC), with 95% confidence intervals. The results of ICCs were present in (**a)** and the results of SCCs were present in (**b)**

To assess the impact of age on the degree of reproducibility of two FFQ measures, we performed subgroup analysis according to age (Fig. 2). As shown in Supplemental Table 2, compared with those for adults aged between 18 and 50 years (median: 0.671, range: 0.510–0.793), the ICCs of reproducibility were lower in adolescents (< 18 years) except those for retinol and vitamin D (median: 0.524, range: 0.290–0.730). The median (range) of ICCs for the elderly (> 50 years) was 0.659 (0.482–0.866), which were lower than those for adults for 22 of 40 nutrients. For pooled SCCs (Supplemental Table 3), the median (range) values between repeated measures was 0.646 (0.516–0.837), 0.608 (0.339–0.873), and 0.469 (0.480–0.724) for adults, the elderly, and adolescents, respectively. SCCs were lower in adolescents than in adults for most nutrients except K, Na, and lipid. Values were higher in adults than in the elderly for 34 of 45 nutrients.

**Fig. 3:**
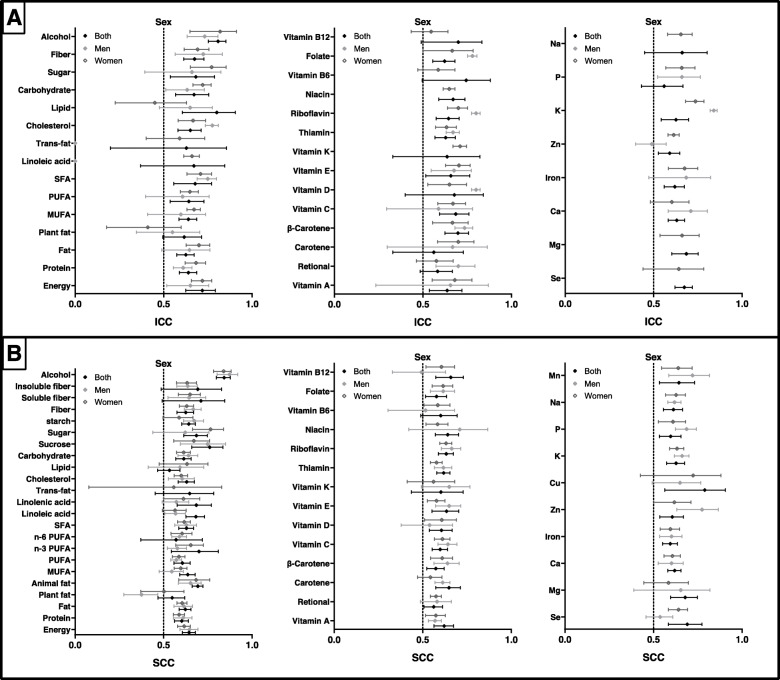
Reproducibility of food frequency questionnaires (FFQ) stratified by sex. Values represent pooled intraclass correlation coefficient (ICC) and spearman correlation coefficient (SCC), with 95% confidence intervals. The results of ICCs were present in (**a)** and the results of SCCs were present in (**b)**

Based on subgroup analysis according to sex (Fig. 3), pooled ICCs for estimation of 13 of 28 and pooled SCCs for estimation of 17 of 46 nutrient intake between two measures were higher in men than in women. The median pooled crude ICC was 0.668 (range: 0.489–0.839) and 0.666 (range: 0.410–0.819) for men and women (Supplemental Table 4). Range of SCCs was between 0.374 and 0.872 for men, and between 0.502 and 0.838 for women (Supplemental Table 5).

### Subgroup analysis according to region

Subgroup analysis according to region revealed that the ICCs of reproducibility ranged from 0.369 (retinol) to 0.829 (thiamin), 0.560 (Zn) to 0.830 (alcohol), 0.400 (vitamin K) to 0.839 (β-carotene), 0.310 (starch) to 0.859 (lipid), and 0.563 (vitamin D) to 0.861 (alcohol) in the regions of Africa, Oceania, Asia, Europe, and America, respectively (Supplemental Table 6 and Table 7). As shown in Supplemental Table 8 and Table 9, pooled crude SCCs ranged from 0.283 (vitamin B6) to 0.723 (Na) for Africa, 0.514 (vitamin A) to 0.907 (alcohol) for Oceania, 0.537 (lipid) to 0.809 (linolenic acid) for Asia, 0.487 (plant fat) to 0.857 (alcohol) for Europe, and 0.413 (vitamin K) to 0.872 (alcohol) for America.

### Factors influencing reproducibility according to study design

**Fig. 4:**
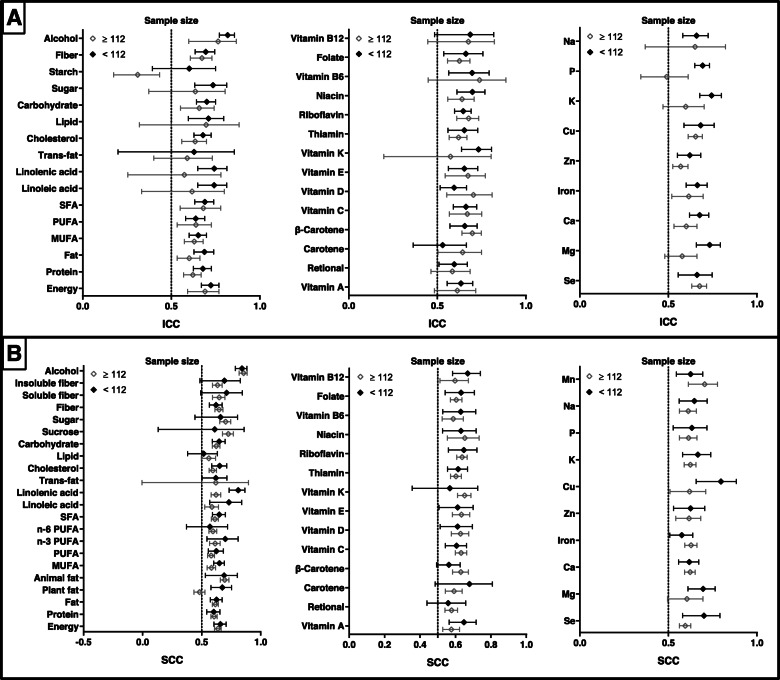
Reproducibility of food frequency questionnaires (FFQ) stratified by sample size. Values represent pooled intraclass correlation coefficient (ICC) and spearman correlation coefficient (SCC), with 95% confidence intervals. The results of ICCs were present in (**a)** and the results of SCCs were present in (**b)**

The results of pooled ICCs and SCCs for reproducibility stratified according to sample size are presented in Fig. 4. The results of pooled ICCs stratified according to sample size are presented in Supplemental Table 10. The median (range) of ICCs in small (≤112) and large sample sizes (> 112) were 0.678 (0.529–0.818) and 0.636 (0.310–0.764), respectively. The ICCs of reproducibility for small sample sizes were higher than those for large sample size for 30 of 39 nutrients. When SCCs were used to assess the reproducibility of FFQs, the values ranged from 0.482 to 0.855 for large sample sizes. The values for small sample sizes varied from 0.516 to 0.841, which were higher than those of large sample sizes for most nutrients (28/46) (Supplemental Table 11).

**Fig. 5:**
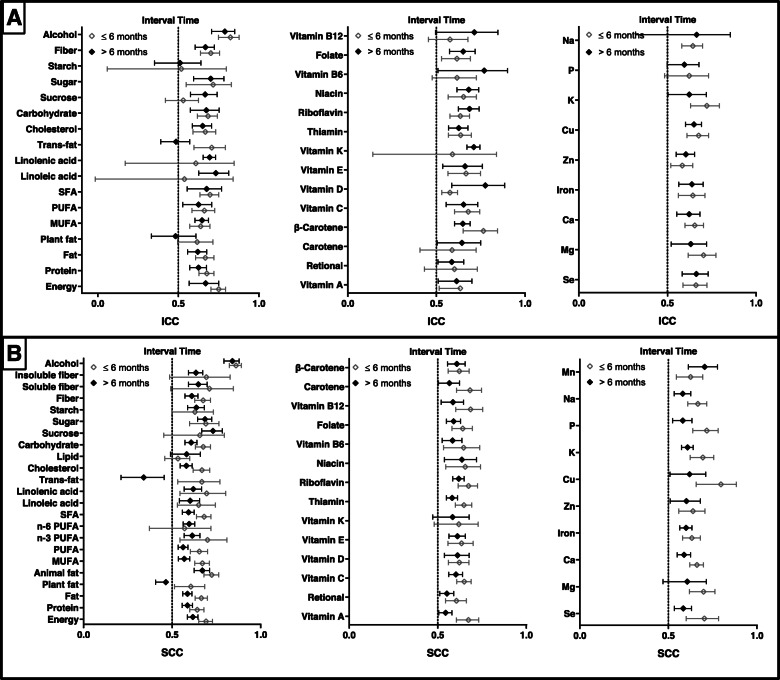
Reproducibility of food frequency questionnaires (FFQ) stratified by interval time. Values represent pooled intraclass correlation coefficient (ICC) and spearman correlation coefficient (SCC), with 95% confidence intervals. The results of ICCs were present in (**a)** and the results of SCCs were present in (**b)**

The results of analysis of subgroups by interval time between two measures of FFQs is present in Fig. 5. And we found that a median (range) of pooled ICCs of 0.643 (0.518–0.822) for short-term reproducibility and 0.652 (0.485–0.788) for long-term reproducibility (Supplemental Table 12). SCCs ranged from 0.532 to 0.860 and 0.339 to 0.840 for short-term and long-term reproducibility, respectively (Supplemental Table 13). For participants with a shorter period (≤6 months) between completing FFQs, pooled ICCs of energy and most nutrient (24/40) intake were higher than those for longer periods (> 6 months). Higher SCCs were identified for most nutrients (42/48) for assessment of the short-term reliability of FFQs when compared with those for long-term reliability.

In order to assess the influence of seasons on the reproducibility of FFQs, we conducted a subgroup analysis with 12-month interval as cut-point. For the long-term and short-term reproducibility of FFQ, the pooled ICC was from 0.501 to 0.859 (median = 0.676) and from 0.485 to 0.788 (median = 0.643), respectively (Supplemental Table 14). Compared with the reproducibility of FFQs at long time intervals (≥ 12 months), the ICCs of FFQs reproducibility at short intervals were higher (28/40). As shown in Supplemental Table 15, the SCCs of reproducibility of FFQ at long intervals (≥ 12 months) were from 0.339 to 0.848 (median = 0.602) and SCCs of reproducibility of FFQ at short intervals (< 12 months) were from 0.248 to 0.845 (median = 0.632). The SCCs for short-term reproducibility of FFQs were higher for energy and most nutrients (34/49) than long-term reproducibility of FFQs.

### Factors influencing reproducibility according to FFQ design

**Fig. 6:**
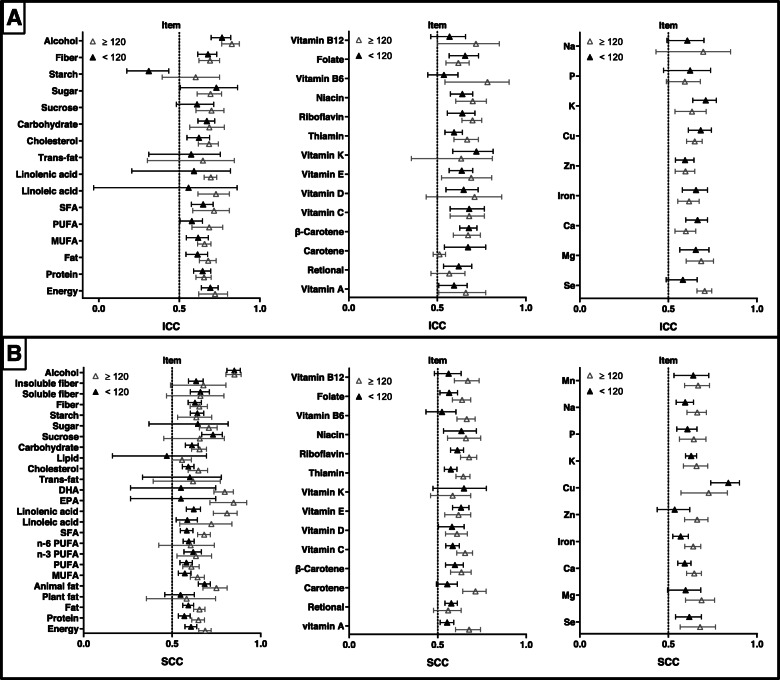
Reproducibility of food frequency questionnaires (FFQ) stratified by items of FFQ. Values represent pooled intraclass correlation coefficient (ICC) and spearman correlation coefficient (SCC), with 95% confidence intervals. The results of ICCs were present in (**a)** and the results of SCCs were present in (**b)**

The results of subgroup analyses according to items of FFQ are presented in Fig. 6. For FFQ items, the pooled ICCs between two measures of FFQs with many items (> 120) varied from 0.512 to 0.825, whereas values of FFQs with small items (≤120) ranged from 0.310 to 0.764 (Supplemental Table 16). The pooled SCCs of long FFQs varied from 0.555 to 0.85, while the values of short FFQs ranged from 0.469 to 0.851 (Supplemental Table 17). Compared with those of short FFQs, pooled ICCs and SCCs of long FFQs were higher for 38 of 39 nutrients and 43 of 49 nutrients, respectively.

**Fig. 7:**
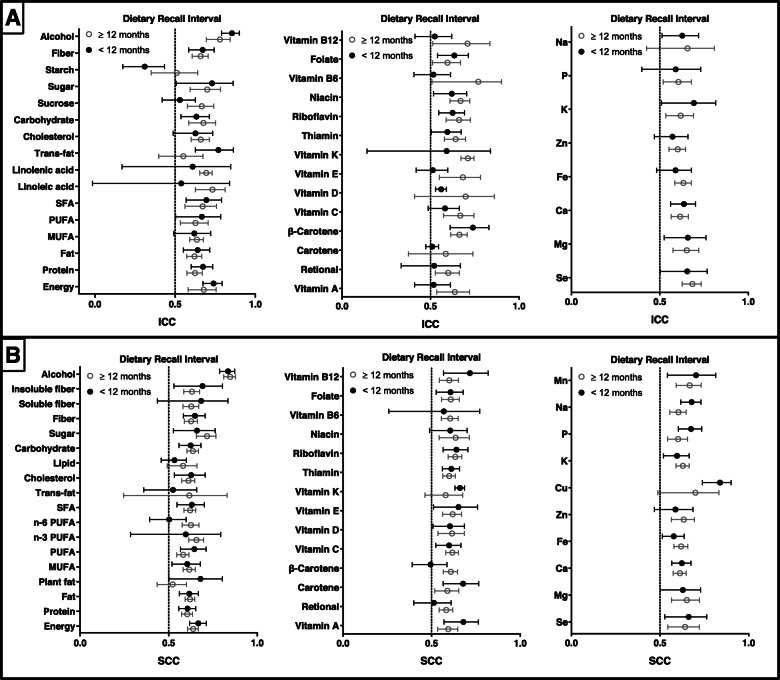
Reproducibility of food frequency questionnaires (FFQ) stratified by dietary recall interval. Values represent pooled intraclass correlation coefficient (ICC) and spearman correlation coefficient (SCC), with 95% confidence intervals. The results of ICCs were present in (**a)** and the results of SCCs were present in (**b)**

ICCs and SCCs for reproducibility stratified according to dietary recall interval are presented in Fig. 7. The median ICC values were 0.659 (range: 0.557–0.836) for long-term FFQs (≥12 months) and 0.622 (range: 0.310–0.854) for short-term FFQs (< 12 months). SCCs ranged from 0.522 to 0.847 and 0.494 to 0.838 for long-term and short-term FFQs, respectively. The combined ICCs of 24/38 nutrients and SCCs of 20/42 nutrients between repeated long-term FFQs were higher than those for short-term FFQs (Supplemental Table 18 and Table 19).

**Fig. 8:**
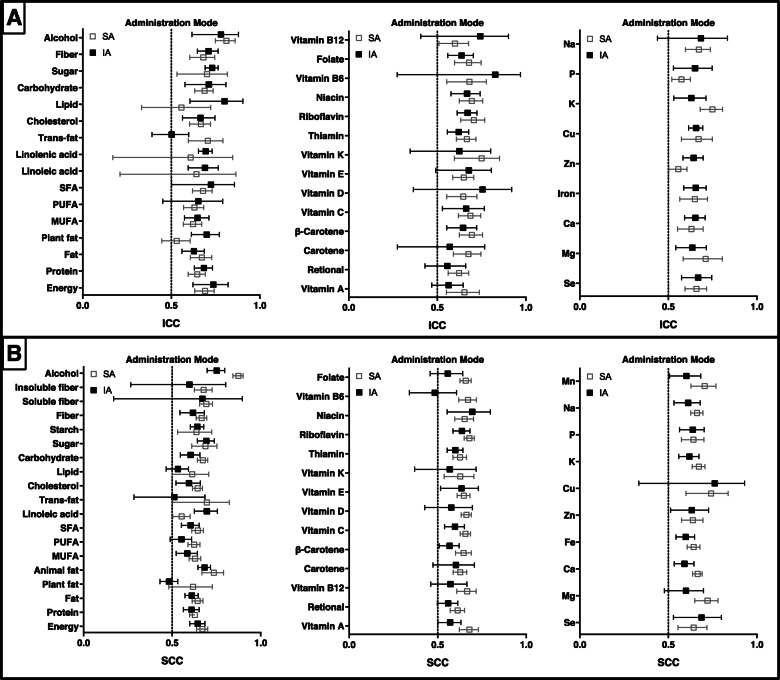
Reproducibility of food frequency questionnaires (FFQ) stratified by administration mode. Values represent pooled intraclass correlation coefficient (ICC) and spearman correlation coefficient (SCC), with 95% confidence intervals. The results of ICCs were present in (**a)** and the results of SCCs were present in (**b)**

Figure 8 present the difference of correlations between self-administered and interviewer-administered FFQs. Pooled ICCs ranged from 0.530 to 0.811 and 0.502 to 0.826 for the reproducibility of self-administered FFQs and interviewer-administered FFQs, respectively (Supplemental Table 20). In total, values for 17/39 nutrients were higher for self-administered FFQs than for interviewer-administered FFQs. SCCs for the reproducibility of self-administered FFQs (range: 0.553–0.874) were higher than those for interviewer-administered FFQs (range: 0.482–0.761) for 37 of 43 nutrients (Supplemental Table 21).

## Discussion

In the present study, we conducted a meta-analysis to systematically assess the reproducibility of FFQs and to explore the factors related to the reproducibility of FFQs. And the pooled ICCs and SCCs were found exceeded 0.5 for energy and most nutrients in general heathy populations. For the elderly and adolescents, pooled ICCs and SCCs for most nutrients were lower than those in adults (18–50 years old). In terms of energy and 24 macronutrients, all ICC and SCC values exceeded 0.5, except for I, soluble fiber, trans-fat, n-3 PUFA, and n-6 PUFA. Moreover, we identified that FFQs with more food items, 12 months as dietary recall interval, and shorter time periods between repeated FFQs resulted in superior FFQ reproducibility.

To evaluate the ability of FFQs to accurately evaluate long-term dietary intake in different age groups, we conducted subgroup analysis according to age which revealed that the correlations of the reproducibility of FFQs exceeded 0.5 for most nutrients in the elderly, adolescents, and adults, indicating that the reliability of FFQs was relatively consistent across age groups. However, the reproducibility of FFQs for adults was higher than that for the elderly and adolescents for most nutrients. A potential reason for the lower correlation in adolescents was that older individuals may have more established dietary habits than younger individuals [55]. Further, in adolescents, it is more challenging to assess dietary intake levels, particularly for cooking-related ingredients such as spices [123], and to understand abstract concepts of average intake, particularly for seasonal food such as fruits [37], although the ability to self-report food intake in adolescents improves rapidly from 8 years of age [135]. Compared with that in adults, the reproducibility of FFQs in the elderly tended to be poor. Although the elderly have a relatively stable dietary intake, a decline in memory or cognitive function may have contributed to the tendency for poor reproducibility in the elderly [70].

Gender differences in the reproducibility of FFQs were observed in this study. The degree of reproducibility was generally higher in women than in men for most nutrients, suggesting that women have more stable long-term dietary intake than that of men [21]. Generally, women pay more attention to food intake and cook more often [79], which may contribute to the higher reproducibility of FFQs in women.

In addition, we observed that the reproducibility of FFQ was low when the sample size was large. This low correlation was not the true reproducibility coefficients between FFQs, but might be caused by irrelevant factors in the operation process. As the large sample sizes may facilitate the management of more participants; consume time, resources, and effort; induce loss to follow-up and put a burden on researchers. However, a small sample size may limit representativeness, which induces large differences in within-person nutrient intakes, leading to less reliable correlation coefficients and ICCs [122]. Therefore, when conducting FFQ reproducibility research, a sample size with sufficient statistical power is recommended to ensure reproducibility of FFQs, rather than increasing sample size blindly.

FFQs with more items presented better reproducibility for most nutrients, indicating that long FFQs collated more reliable information [136] and enabled better estimations of dietary and nutrient consumption [92]. However, participants require more time to accurately complete the questionnaire and may lose patience, leading to potential biases and, ultimately, data of lower quality [2]. Therefore, to balance reporting errors and reproducibility of FFQs, pilot studies should be performed to explore the appropriate number of FFQ items based on the demographic characteristics of participants.

FFQs were used to assess regular dietary habits over extended periods. The correlation coefficients of the study assessing the reproducibility of FFQs over more than a 1-year period were higher than those over short periods for most nutrients. Relatively high correlations for 1 year indicated that FFQs can provide an accurate estimation of long-term dietary habits. The reasons for lower correlations of FFQs over less than 1 year may be related to the seasonal availability of food [130]. In addition, it is useful for researchers to assess the complete dietary intake of participants with 1 year as the reference time for FFQs [130].

The combined correlation coefficients were higher when FFQs were administered over a short period (≤6 months) compared with those over a long time interval (> 6 months), suggesting that shorter intervals between repeated FFQ administrations were a key factor contributing to high reproducibility of FFQs, in accordance with a previous review [2]. A possible explanation for the higher correlations for short-term reproducibility is that it is easier for respondents to remember and replicate their previous FFQ responses accurately when two FFQs are administered closely in time [15]. The difference between the two subgroups may also be because of changes in diet over time [137]. The lower correlation of long-term reproducibility suggested that the participants’ usual intake of food may have changed during the study period [134]. Because food intake also exhibits yearly trends [138, 139], longer intervals between repeated FFQs were selected to avoid the effects of seasonal or yearly variations in diet [82]. Therefore, before selecting intervals between repeated FFQs, memory bias and seasonal changes in diet should be taken into consideration.

The main strength of this study is that it is the first meta-analysis to comprehensively analyze FFQ reproducibility. Current research is based on a large number of different populations with a wide age range which revealed good reproducibility of nutrient intake, making FFQs suitable for analyzing dietary intake among different subgroups of age, sample size, gender, and region. We comprehensively evaluated the reproducibility of FFQs by analyzing the intake of 50 nutrients, which strengthened the conclusions of this study.

This study has some limitations. First, our screening criteria excluded articles that assessed the effectiveness of specific nutrients, which may have affected our results in different ways. Second, learning ability and lifestyles, such as education level and body mass index, may have influenced FFQ reliability. However, the relevant data were not available in the included articles. Third, we did not evaluate the quality of included studies because there are currently no tools to assess the quality of reproducibility studies for FFQs. Further studies are needed to establish such tools to evaluate the quality of reproducibility studies for FFQ.

## Conclusions

In conclusion, FFQs with correlation coefficients greater than 0.5 for most nutrients may be considered a reliable tool to measure dietary intake. In addition, factors related to FFQ design may be associated with the reproducibility of FFQs, such as FFQ items and dietary recall intervals. To increase the reproducibility of FFQs, the following points should be considered before developing FFQs. First, pilot studies are warranted to explore the appropriate number of FFQ items based on the characteristics of the study population. Second, 12 months is suggested as the dietary recall interval. Third, when performing reproducibility studies for FFQs, a sample size with sufficient statistical power, but no larger, is recommended.

## Supplementary Information

**Additional file 1.** PRISMA Checklist.

**Additional file 2 Supplemental Table 1.** The overview of the retrieved studies assessing the reproducibility of FFQs.

**Additional file 3 Supplemental Table 2.** Pooled intraclass correlation coefficients for energy and nutrients stratified by age.

**Additional file 4 Supplemental Table 3.** Pooled spearman correlation coefficients for energy and nutrients stratified by age.

**Additional file 5 Supplemental Table 4.** Pooled intraclass correlation coefficients for energy and nutrients stratified by sex.

**Additional file 6 Supplemental Table 5.** Pooled spearman correlation coefficients for energy and nutrients stratified by sex.

**Additional file 7 Supplemental Table 6.** Pooled crude intraclass correlation coefficients for energy and nutrients stratified by regions.

**Additional file 8 Supplemental Table 7.** Pooled energy-adjusted intraclass correlation coefficients for energy and nutrients stratified by regions.

**Additional file 9 Supplemental Table 8.** Pooled crude spearman correlation coefficients for energy and nutrients stratified by regions.

**Additional file 10 Supplemental Table 9.** Pooled energy-adjusted spearman correlation coefficients for energy and nutrients stratified by regions.

**Additional file 11 Supplemental Table 10.** Pooled intraclass correlation coefficients for energy and nutrients stratified by sample size.

**Additional file 12 Supplemental Table 11.** Pooled spearman correlation coefficients for energy and nutrients stratified by sample size.

**Additional file 13 Supplemental Table 12.** Pooled intraclass correlation coefficient for energy and nutrients stratified by time interval (6 months as cut-point).

**Additional file 14 Supplemental Table 13.** Pooled spearman correlation coefficient for energy and nutrients stratified by time interval (6 months as cut-point).

**Additional file 15 Supplemental Table 14.** Pooled intraclass correlation coefficient for energy and nutrients stratified by time interval (12 months as cut-point).

**Additional file 16 Supplemental Table 15.** Pooled spearman correlation coefficient for energy and nutrients stratified by time interval (12 months as cut-point).

**Additional file 17 Supplemental Table 16.** Pooled intraclass correlation coefficient for energy and nutrients stratified by items of FFQ.

**Additional file 18 Supplemental Table 17.** Pooled spearman correlation coefficient for energy and nutrients stratified by items of FFQ.

**Additional file 19 Supplemental Table 18.** Pooled intraclass correlation coefficient for energy and nutrients stratified by dietary recall interval.

**Additional file 20 Supplemental Table 19.** Pooled spearman correlation coefficient for energy and nutrients stratified by dietary recall interval.

**Additional file 21 Supplemental Table 20.** Pooled intraclass correlation coefficient for energy and nutrients stratified by administration mode.

**Additional file 22 Supplemental Table 21.** Pooled spearman correlation coefficient for energy and nutrients stratified by administration mode.

## Data Availability

Not applicable.

## Abbreviations

I^2^: Inconsistency index
ICCs: Intraclass coefficients
I: Iodine
K: Potassium
Na: Sodium
SCC: Spearman correlation coefficients
Zn: Zinc
