# Supplementary material for: A meta-analysis of the reproducibility of food frequency questionnaires in nutritional epidemiological studies
Source: Int J Behav Nutr Phys Act. 2021 Jan 11;18:12. doi: 10.1186/s12966-020-01078-4 (PMC7802360; doi:10.1186/s12966-020-01078-4)
Supplement: Supplementary file 1 — Additional file 1. PRISMA Checklist. [file 12966_2020_1078_MOESM1_ESM.doc]

| **Section/topic** | **#** | **Checklist item T** | **Reported on page #** |
| --- | --- | --- | --- |
| **TITLE** | | |  |
| Title | 1 | Identify the report as a systematic review, meta-analysis, or both. | Title |
| **ABSTRACT** | | |  |
| Structured summary | 2 | Provide a structured summary including, as applicable: background; objectives; data sources; study eligibility criteria, participants, and interventions; study appraisal and synthesis methods; results; limitations; conclusions and implications of key findings; systematic review registration number. | Abstract |
| **INTRODUCTION** | | |  |
| Rationale | 3 | Describe the rationale for the review in the context of what is already known. | Introduction, paragraphs 1-3 |
| Objectives | 4 | Provide an explicit statement of questions being addressed with reference to participants, interventions, comparisons, outcomes, and study design (PICOS). | Introduction, paragraphs 4 |
| **METHODS** | | |  |
| Protocol and registration | 5 | Indicate if a review protocol exists, if and where it can be accessed (e.g., Web address), and, if available, provide registration information including registration number. | Methods, paragraph 1 |
| Eligibility criteria | 6 | Specify study characteristics (e.g., PICOS, length of follow-up) and report characteristics (e.g., years considered, language, publication status) used as criteria for eligibility, giving rationale. | Methods/Study Identification and Selection  “Articles were included if they met the following criteria: (1) FFQs were used to measure nutrient intake; (2) the age range of target healthy populations was between 8 and 86 years; (3) the study assessed the reproducibility of FFQs; (4) the study was published in English; and (5) the reproducibility of FFQs was measured with the intraclass correlation coefficient (ICC) and Pearson correlation coefficient or SCC.  The exclusion criteria were: (1) food intake was assessed using FFQs; (2) FFQs were used to assess a specific nutrient; (3) the target population was unhealthy people or specific populations, such as individuals who were overweight or malnourished; (4) the participants were less than 8 years old; (5) the article investigated diet-disease relationships; and (6) the full text was unavailable through web searches.” |
| Information sources | 7 | Describe all information sources (e.g., databases with dates of coverage, contact with study authors to identify additional studies) in the search and date last searched. | Methods/Literature Search  “PubMed and Web of Science databases before July 2020” |
| Search | 8 | Present full electronic search strategy for at least one database, including any limits used, such that it could be repeated. | Methods/Literature Search  “The search strategy used employed the terms “FFQ OR food frequency questionnaire” AND “reproducibility OR repeatability OR reliability”.” |
| Study selection | 9 | State the process for selecting studies (i.e., screening, eligibility, included in systematic review, and, if applicable, included in the meta-analysis). | Methods/Study Identification and Selection  “The original studies were obtained from the database. After removing duplicates, we screen the studies according to title and abstract. After reading the full texts, the eligible articles were obtained by exclusion criteria.” |
| Data collection process | 10 | Describe method of data extraction from reports (e.g., piloted forms, independently, in duplicate) and any processes for obtaining and confirming data from investigators. | Methods/ Data Extraction  “Data were extracted from each study by independent reviewers.” |
| Data items | 11 | List and define all variables for which data were sought (e.g., PICOS, funding sources) and any assumptions and simplifications made. | Methods/ Data Extraction  “The extracted contents included the following, excluding authors and published years: (1) characteristics of participants including sample size, age, gender distribution, and region; (2) characteristics of FFQs including food items and dietary recall interval; (3) characteristics of study design including administration method and interval between two FFQs; and (4) statistics employed to assess reproducibility between repeated FFQs including ICC and Pearson correlation coefficient or SCC in relation to energy, macronutrients, and micronutrients (minerals and vitamins).” |
| Risk of bias in individual studies | 12 | Describe methods used for assessing risk of bias of individual studies (including specification of whether this was done at the study or outcome level), and how this information is to be used in any data synthesis. | Not available |
| Summary measures | 13 | State the principal summary measures (e.g., risk ratio, difference in means). | Methods/ Meta-Analysis  “The pooled correlation coefficients were calculated based on the ICC and SCC values obtained from each article.” |
| Synthesis of results | 14 | Describe the methods of handling data and combining results of studies, if done, including measures of consistency (e.g., I^2^) for each meta-analysis. | Methods/ Meta-Analysis  “After appropriate conversion, random effects meta-analyses were used to combine data. The heterogeneity of the z-values among studies was determined by calculating the inconsistency index (I^2^).” |

Page 1 of 2

| **Section/topic** | **#** | **Checklist item** | **Reported on page #** |
| --- | --- | --- | --- |
| Risk of bias across studies | 15 | Specify any assessment of risk of bias that may affect the cumulative evidence (e.g., publication bias, selective reporting within studies). | Methods/ Meta-Analysis  “Sensitivity analysis was performed to explore the when to further explore the source of heterogeneity.” |
| Additional analyses | 16 | Describe methods of additional analyses (e.g., sensitivity or subgroup analyses, meta-regression), if done, indicating which were pre-specified. | Methods/ Meta-Analysis  “Sensitivity analysis was performed to explore the when to further explore the source of heterogeneity.” |
| **RESULTS** | | |  |
| Study selection | 17 | Give numbers of studies screened, assessed for eligibility, and included in the review, with reasons for exclusions at each stage, ideally with a flow diagram. | Results/ Literature Search and Study Selection  Fig 1 |
| Study characteristics | 18 | For each study, present characteristics for which data were extracted (e.g., study size, PICOS, follow-up period) and provide the citations. | Results/ Study Characteristics  Fig2 and S1 Table |
| Risk of bias within studies | 19 | Present data on risk of bias of each study and, if available, any outcome level assessment (see item 12). | Not available |
| Results of individual studies | 20 | For all outcomes considered (benefits or harms), present, for each study: (a) simple summary data for each intervention group (b) effect estimates and confidence intervals, ideally with a forest plot. | Table 2-3 |
| Synthesis of results | 21 | Present results of each meta-analysis done, including confidence intervals and measures of consistency. | Table 2-3 |
| Risk of bias across studies | 22 | Present results of any assessment of risk of bias across studies (see Item 15). | S2 Table |
| Additional analysis | 23 | Give results of additional analyses, if done (e.g., sensitivity or subgroup analyses, meta-regression [see Item 16]). | S2 Table-S19 Table |
| **DISCUSSION** | | |  |
| Summary of evidence | 24 | Summarize the main findings including the strength of evidence for each main outcome; consider their relevance to key groups (e.g., healthcare providers, users, and policy makers). | Discussion  paragraph 1: the main outcomes of this study.  paragraph 2-7: the relevance |
| Limitations | 25 | Discuss limitations at study and outcome level (e.g., risk of bias), and at review-level (e.g., incomplete retrieval of identified research, reporting bias). | Discussion/paragraph 1 |
| Conclusions | 26 | Provide a general interpretation of the results in the context of other evidence, and implications for future research. | Conclusions |
| **FUNDING** | | |  |
| Funding | 27 | Describe sources of funding for the systematic review and other support (e.g., supply of data); role of funders for the systematic review. | Funding information was uploaded to the manuscript submission system. |

*From:*  Moher D, Liberati A, Tetzlaff J, Altman DG, The PRISMA Group (2009). Preferred Reporting Items for Systematic Reviews and Meta-Analyses: The PRISMA Statement. PLoS Med 6(6): e1000097. doi:10.1371/journal.pmed1000097

For more information, visit: **www.prisma-statement.org**.

Page 2 of 2
