# Supplementary material for: A meta-analysis of the reproducibility of food frequency questionnaires in nutritional epidemiological studies
Source: Int J Behav Nutr Phys Act. 2021 Jan 11;18:12. doi: 10.1186/s12966-020-01078-4 (PMC7802360; doi:10.1186/s12966-020-01078-4)
Supplement: Supplementary file 2 — Additional file 2 Supplemental Table 1. The overview of the retrieved studies assessing the reproducibility of FFQs. [file 12966_2020_1078_MOESM2_ESM.docx]

**Supplemental Table 1.** The overview of the retrieved studies assessing the reproducibility of FFQs

| Author (year) | Country | Characteristic of FFQ or reproducibility study | | | | Number of participants | | | Age of participants | |
| --- | --- | --- | --- | --- | --- | --- | --- | --- | --- | --- |
|  |  | Consumption  interval | Administration mode | Interval | Item | Total | Women | Men | Range | Mean |
| Ahn (2007)[[1](#_ENREF_1)] | Korea | 12 months | N/A | 12 months | 103 | 124 | 89 | 35 | 40-69 | 48.8 |
| Amog (2010)[[2](#_ENREF_2)] | Vietnam | 6 months | IA | 4 weeks | 170 | 180 | 97 | 83 | 11-15 | N/A |
| Bae (2010)[[3](#_ENREF_3)] | Korea | N/A | N/A | 9 months | 100 | 38 | 38 | 0 | 20-29 | 21.9 |
| Barrat (2012)[[4](#_ENREF_4)] | France | 0.25 months | SA | 3 weeks | 50 | 54 | 13 | 41 | N/A | 37.1 |
| Bjerregaard (2016)[[5](#_ENREF_5)] | Denmark | 1 month | SA | 4 weeks | 145 | 48 | 29 | 19 | 13-15 | 13.5 |
| Bohlscheid (1997)[[6](#_ENREF_6)] | Germany | 12 months | SA | 6 months | 158 | 104 | 55 | 49 | 35-64 | N/A |
| Boucher (2006)[[7](#_ENREF_7)] | Canada | N/A | SA | 56 days | 126 | 96 | 96 | 0 | 25-74 | N/A |
| Cantin (2016)[[8](#_ENREF_8)] | Quebec | 1 month | SA | 3-5 weeks | 157 | 47 | 26 | 21 | 19-86 | 53 |
| Cardoso (2001)[[9](#_ENREF_9)] | Brazil | 12 months | SA | 1 month | 120 | 77 | 77 | 0 | 21-65 | 35 |
| Collins (2014)[[10](#_ENREF_10)] | Australia | 6 months | SA | 6 months | 120 | 67 | 47 | 20 | N/A | 44.9 men 41.3women |
| Dehghan (2012)[[11](#_ENREF_11)] | Argentina | 12 months | N/A | 12 months | 96 | 156 | 116 | 40 | 35-70 | 52.7 |
| Dehghan (2012)[[12](#_ENREF_12)] | Poland | 12 months | IA | 12 months | 134 | 146 | 73 | 73 | 30-70 | 54.25 |
| Deschamps (2009)[[13](#_ENREF_13)] | France | 12 months | N/A | 12 months | 124 | 57 | 29 | 28 | 18-63 | N/A |
| Doustmohammadian (2020)[[14](#_ENREF_14)] | Iran | 1 month | IA | 6 months | 142 | 230 | 120 | 110 | 18-65 | N/A |
| Dumartheray (2006)[[15](#_ENREF_15)] | Switzerland | 12 months | IA | 1 month  /12 months | 110 | 15 | 15 | 0 | 75-87 | 80.3 |
| Egami (1999)[[16](#_ENREF_16)] | Japan | 12 months | SA | 12 months | 97 | 88 | 42 | 46 | 41-88 | N/A |
| El (2018)[[17](#_ENREF_17)] | Morocco | 12 months | N/A | 1 month | 255 | 87 | 61 | 26 | N/A | 27.3 |
| Elorriaga (2015)[[18](#_ENREF_18)] | Latin America | 12 months | SA | 3 months | 126 | 147 | 95 | 52 | 21-74 | 45.4 |
| Engle (2009)[[19](#_ENREF_19)] | US | 3 months | SA | 1 month | 85 | 50 | 34 | 16 | 26-69 | 49.3 |
| Fallaize (2014)[[20](#_ENREF_20)] | UK | 1 month | SA | 4 weeks | 157 | 100 | 69 | 31 | N/A | 31.5 |
| Fernández (2010)[[21](#_ENREF_21)] | Spain | 12 months | SA | 12 months | 137 | 158 | 85 | 73 | 55-80 | 65.5 |
| Field (1999)[[22](#_ENREF_22)] | US | 12 months | SA | 12 months | 97 | 109 | 57 | 52 | 8-10 | N/A |
| Filippi (2014)[[23](#_ENREF_23)] | Italy | 6 months | SA | 1 month | 106 | 185 | 46 | 139 | 14-17 | 15.9 |
| Flagg (2000)[[24](#_ENREF_24)] | US | 12 months | SA | 12 months | 68 | 427 | 214 | 214 | N/A | 61 |
| Franceschi (1995)[[25](#_ENREF_25)] | Italy | 12 months | IA | 3-10 months | 77 | 452 | 308 | 144 | 35-69 | 50 |
| Friis (1997)[[26](#_ENREF_26)] | Denmark | 12 months | SA | 4 months | 122 | 122 | 122 | 0 | 20-29 | 24.3 |
| Gilsing (2018)[[27](#_ENREF_27)] | Canada | 12 months | SA | 3 months | 36 | 232 | 117 | 115 | 45-87 | N/A |
| Gnardellis (1994)[[28](#_ENREF_28)] | Greece | 12 months | SA | 12 months | 190 | 80 | 38 | 42 | 25-67 | N/A |
| Goulet (2004)[[29](#_ENREF_29)] | Sweden | 1 months | IA | 1 month | 91 | 32 | 17 | 15 | 25-70 | 46.8 |
| Hebden (2013)[[30](#_ENREF_30)] | Australia | 1 month | SA | 1 week | 74 | 77 | 50 | 27 | 18-31 | 23.5 |
| Hernandez (1998)[[31](#_ENREF_31)] | Mexico | 12 months | IA | 12 months | 85 | 134 | 134 | 0 | N/A | N/A |
| Ibiebele (2009)[[32](#_ENREF_32)] | Australia | 12 months | SA | 12 months | 135 | 100 | 50 | 50 | 22-79 | N/A |
| Imaeda (2007)[[33](#_ENREF_33)] | Japan | N/A | SA | 12 months | 47 | 1918 | 1074 | 844 | 23-86 | 56.6 men  57.0 women |
| Iqbal (2009)[[34](#_ENREF_34)] | India | 12 months | SA | 4 months | 132 | 100 | 74 | 26 | N/A | 50.7 |
| Ishihara (2003)[[35](#_ENREF_35)] | Japan | 12 months | SA | 12 months | 44 | 289 | 146 | 143 | 40-69 | 58 men  55 women |
| Ishihara (2009)[[36](#_ENREF_36)] | Brazil | 12 months | IA | 12 months | 118 | 55 | 55 | 0 | 33-72 | 55 |
| Jackson (2013)[[37](#_ENREF_37)] | Botswana | 12 months | IA | 12 months | 122 | 79 | 63 | 16 | 18–75 | 39.9 men 37.2 women |
| Jackson (2001)[[38](#_ENREF_38)] | Spain | 12 months | IA | 12 months | 70 | 123 | 73 | 50 | 25-74 | 45.2 men 43.6 women |
| Jackson (2011)[[39](#_ENREF_39)] | US | 12 months | IA | 12 months | 120 | 70 | 39 | 31 | N/A | 40.9 men 41.5women |
| Jain (2003)[[40](#_ENREF_40)] | Canada | 12 months | SA | 12 months | 131 | 310 | 159 | 151 | N/A | 54.7 men 54.2 women |
| Johansson (2002)[[41](#_ENREF_41)] | Sweden | N/A | SA | 12 months | 84 | 195 | 99 | 96 | ≥ 30 | N/A |
| Johansson (1997)[[42](#_ENREF_42)] | Norway | 12 months | SA | 6 months | 180 | 90 | 38 | 52 | 16-79 | 44 |
| Juliana (2007)[[43](#_ENREF_43)] | Brazil | 12 months | IA | 6 months | 54 | 93 | 0 | 93 | 18-70 | 35.8 |
| Katsouyanni (1997)[[44](#_ENREF_44)] | Greece | 12 months | SA | 12 months | 190 | 80 | 38 | 42 | 25-67 | N/A |
| Kesse-Guyot (2010)[[45](#_ENREF_45)] | France | 12 months | SA | 12 months | 240 | 140 | 74 | 66 | N/A | 63.1 |
| Kim (2015)[[46](#_ENREF_46)] | Korean | 12 months | IA | 9 months | 109 | 126 | 63 | 63 | 20-65 | 42.7 |
| Kristal (2014)[[47](#_ENREF_47)] | US | 3 months | SA | 3 months | 156 | 74 | 34 | 40 | 18-69 | N/A |
| Labonte (2012)[[48](#_ENREF_48)] | France | 1 month | SA | 4–6 weeks | 136 | 74 | 40 | 34 | 18-65 | 37.1 |
| Lazarus (1995)[[49](#_ENREF_49)] | Australia | N/A | N/A | 4 weeks | 170 | 62 | 38 | 24 | 65-88 | 78 |
| Lee (2016)[[50](#_ENREF_50)] | Korea | 12 months | SA | 3 months | 146 | 65 | 48 | 17 | 38-62 | 46 |
| Lee (2006)[[51](#_ENREF_51)] | China | 6 months | IA | 3 months | 64 | 83 | 25 | 58 | N/A | N/A |
| Leon (2015)[[52](#_ENREF_52)] | US | 12 months | SA | 4 weeks | 142 | 56 | 28 | 28 | 18-35 | N/A |
| Li (2005)[[53](#_ENREF_53)] | China | 12 months | IA | 3 months | 125 | 102 | 63 | 39 | 23-55 | 37 men  35 women |
| Longnecker (1993)[[54](#_ENREF_54)] | US | 12 months | SA | 6-12 months | 116 | 138 | 74 | 64 | 22-82 | 49 |
| Lyu (2007)[[55](#_ENREF_55)] | China | 6 months | IA | 7.5 months | 216 | 22 | 20 | 2 | 20-62 | 29 |
| Macedo-Ojeda (2013)[[56](#_ENREF_56)] | Mexico | 12 months | IA | 12 months | 162 | 97 | 59 | 38 | 18-71 | 27.5 |
| MacIntyre (2001)[[57](#_ENREF_57)] | South Africa | N/A | IA | 6-12 weeks | 145 | 144 | 99 | 45 | 15-65 | N/A |
| Malekshah (2006)[[58](#_ENREF_58)] | Iran | 12 months | SA | 2 months | 158 | 131 | 80 | 51 | 35-65 | 51.2 men  49.9 women |
| Männistö (1996)[[59](#_ENREF_59)] | Finland | 12 months | SA | 3 months | 110 | 152 | 152 | 0 | 25-75 | 51 |
| Marchioni (2007) [[60](#_ENREF_60)] | Brazil | 6 months | SA | 3 months | 76 | 49 | 24 | 25 | 16-19 | N/A |
| María (2015)[[61](#_ENREF_61)] | Argentina | 12 months | IA | 10 months | 66 | 88 | 53 | 35 | 18-65 | 33.8 |
| Marques-Vidal (2011)[[62](#_ENREF_62)] | Switzerland | 1 month | N/A | 1 months | 126 | 40 | 17 | 23 | 25-65 | 43.1 |
| Marshall (2016)[[63](#_ENREF_63)] | Europe | 1 month | SA | 1 month | 157 | 567 | 334 | 233 | N/A | 38.7 |
| Martinez (2013)[[64](#_ENREF_64)] | Brazil | 12 months | N/A | 3 months | 50 | 109 | 49 | 60 | 13-17 | 16 |
| Martin-Moreno (1993)[[65](#_ENREF_65)] | Spain | 12 months | IA | 3 months | 118 | 147 | 147 | 0 | 18-74 | N/A |
| Maruyama (2015)[[66](#_ENREF_66)] | Japan | 12 months | SA | 3 months | 81 | 58 | 29 | 29 | 30-79 | N/A |
| Marventano (2016)[[67](#_ENREF_67)] | Italy | 6 months | SA | 10 months | 110 | 178 | 116 | 62 | 18-80 | 56.2 |
| Maryam (2017)[[68](#_ENREF_68)] | Iran | 1 month | N/A | 3 months | 160 | 59 | 39 | 20 | 20-69 | 35 |
| McKeown (2001)[[69](#_ENREF_69)] | UK | 12 months | SA | 6 months | 130 | 146 | 88 | 58 | 45-74 | 60 men  58 women |
| McPherson (1995)[[70](#_ENREF_70)] | US | 1 month | N/A | 1 months | 188 | 20 | 10 | 10 | 35-41 | N/A |
| Messerer (2004)[[71](#_ENREF_71)] | Sweden | 12 months | SA | 12 months | 88 | 248 | 0 | 248 | 40-74 | 60.8 |
| Tijerina (2020)[[72](#_ENREF_72)] | Mexico | 12 months | IA | 12 months | 136 | 200 | 200 | 0 | 40-65 | 50.6 |
| Mirmiran (2010)[[73](#_ENREF_73)] | Iran | 12 months | IA | 12 months | 168 | 132 | 71 | 61 | ≥ 20 | 35.5 |
| Morris (2003)[[74](#_ENREF_74)] | US | 12 months | SA | 12 months | 139 | 232 | 125 | 107 | 68-99 | 77.8 |
| Munger (1992)[[75](#_ENREF_75)] | US | 12 months | SA | 3-4 months | 126 | 44 | 44 | 0 | 55-69 | 61.7 |
| Nelida (2003)[[76](#_ENREF_76)] | Brazil | 6 months | IA | 4 months | 127 | 104 | 62 | 42 | 18-60 | 28.5 men 38.8 women |
| Ocke (1997)[[77](#_ENREF_77)] | Netherland | 12 months | SA | 6 months/  12 months | 79 | 121 | 58 | 63 | 20-70 | N/A |
| Ogawa (2003)[[78](#_ENREF_78)] | Japan | 12 months | SA | 12 months | 40 | 113 | 58 | 55 | 45–77 | 62.1 men  61 women |
| Overby (2014)[[79](#_ENREF_79)] | Norway | 4 weeks | SA | 4 weeks | 131 | 58 | 36 | 22 | 13-14 | 13.8 |
| Palacios (2015)[[80](#_ENREF_80)] | US | 12 months | IA | 1 months | 193 | 92 | 63 | 29 | ≥ 21 | 28.9 |
| Park (2012)[[81](#_ENREF_81)] | Korea | 12 months | IA | 12 months | 112 | 288 | 173 | 115 | 30-66 | 44.7 |
| Preston (2011)[[82](#_ENREF_82)] | puerto rico | 1 month | IA | 2 weeks | 97 | 94 | 54 | 40 | N/A | 10.4 |
| Riboli (1997)[[83](#_ENREF_83)] | US | 12 months | SA | 2-3 months/  2-4 years | 71 | 474 | 474 | 0 | 35-65 | N/A |
| Rifas (2001)[[84](#_ENREF_84)] | US | 12 months | SA | 2 weeks | 131 | 160 | 91 | 69 | 19-65 | 48 |
| Rimm (1992)[[85](#_ENREF_85)] | US | 12 months | SA | 12 months | 131 | 127 | 0 | 127 | 40-75 | N/A |
| Rockett (1995)[[86](#_ENREF_86)] | US | 12 months | SA | 50 weeks | 116 | 179 | 101 | 75 | 9-18 | 14 |
| Rodriguez (2017)[[87](#_ENREF_87)] | Peru | 12 months | N/A | 6 months | 150 | 53 | 30 | 23 | 8-14 | 11 |
| Salvesen (2019)[[88](#_ENREF_88)] | Norway | 1 month | SA | 19 days | 146 | 21 | 17 | 4 | 20-30 | 23 |
| Sam (2014)[[89](#_ENREF_89)] | New Zealand | 12 months | SA | 9 months | 154 | 132 | 107 | 25 | 30-59 | 44 |
| Sam (2020)[[90](#_ENREF_90)] | New Zealand | 12 months | N/A | 9 months | 57 | 132 | 67 | 65 | 30-59 | 44 |
| Sanjeevi (2017)[[91](#_ENREF_91)] | US | 0.25 months | IA | 1 months | 95 | 70 | 70 | 0 | 18-37 | 36.8 |
| Sasaki (2003)[[92](#_ENREF_92)] | Japan | 12 months | SA | 12 months | 44 | 209 | 108 | 101 | N/A | N/A |
| Schroder (2001)[[93](#_ENREF_93)] | Spain | N/A | SA | 6 weeks | 157 | 44 | 31 | 13 | N/A | 30.7 |
| Selem (2014)[[94](#_ENREF_94)] | Brazil | 12 months | N/A | 12 months | 60 | 74 | 42 | 32 | ≥ 20 | N/A |
| Shai (2005)[[95](#_ENREF_95)] | Israel | N/A | SA | 12 months | 126 | 161 | 126 | 35 | ≥ 35 | 50 |
| Shimizu (1999)[[96](#_ENREF_96)] | Japan | 12 months | SA | 12 months | 169 | 117 | 59 | 58 | 35-64 | 57.9 men  53.8 women |
| Shu (2004)[[97](#_ENREF_97)] | China | 12 months | IA | 2 years | 77 | 191 | 191 | 0 | 40-70 | 55.4 |
| Silva-Jaramillo (2015)[[98](#_ENREF_98)] | Ecuador | 12 months | IA | 30-40 days | 111 | 95 | 61 | 34 | 20-65 | 40 |
| Smith (1998)[[99](#_ENREF_99)] | Australia | 12 months | SA | 4-6 weeks/  15 months | 145 | 131 | 83 | 48 | 63-80 | 67.7 |
| Sudha (2006)[[100](#_ENREF_100)] | India | 12 months | IA | 6 months | 222 | 102 | 68 | 34 | ≥ 20 | 40.9 |
| Takachi (2011)[[101](#_ENREF_101)] | Japan | 12 months | SA | 2.7 years | 138 | 143 | 74 | 69 | 40-60 | N/A |
| Tang (2015)[[102](#_ENREF_102)] | China | N/A | IA | 4 weeks | 120 | 110 | 110 | 0 | 12-44 | N/A |
| Tavani (1995)[[103](#_ENREF_103)] | Italy | 12 months | IA | 3-6 months | 77 | 452 | 308 | 144 | 35-69 | N/A |
| Tollosa (2017)[[104](#_ENREF_104)] | Belgium | 12 months | SA | 2 weeks | 109 | 162 | 96 | 66 | ≥50 | 57.5 |
| Tsubono (2003)[[105](#_ENREF_105)] | Japan | 1 month | N/A | 5 years | 44 | 201 | 107 | 94 | 40-59 | N/A |
| Tsubono (2001)[[106](#_ENREF_106)] | Japan | 12 months | SA | 12 months | 141 | 113 | 58 | 55 | N/A | 62.1 men  61 women |
| Turconi (2010)[[107](#_ENREF_107)] | Italy | 12 months | IA | 6 weeks | 189 | 112 | 60 | 52 | 20-60 | 39.4 |
| Villegas (2007)[[108](#_ENREF_108)] | China | 12 months | IA | 12 months | 81 | 195 | 0 | 195 | 40-70 | 54·8 |
| Vereecken (2010)[[109](#_ENREF_109)] | Belgium | 2 months | SA | 1-2 weeks | 137 | 48 | 22 | 26 | 13-17 | 14.6 |
| Watanabe (2011)[[110](#_ENREF_110)] | Japan | 1 month | SA | 1 months | 82 | 63 | 63 | 0 | 12-13 | N/A |
| Watson (2009)[[111](#_ENREF_111)] | Australia | 6 months | SA | 5 months | 120 | 101 | 65 | 36 | 9-16 | 11.9 |
| Whitton (2017)[[112](#_ENREF_112)] | Singapore | 12 months | IA | 6 months | 163 | 161 | 80 | 81 | 18-79 | 44 |
| Willett (1985)[[113](#_ENREF_113)] | US | 12 months | N/A | 12 months | 61 | 173 | 173 | 0 | 34-59 | N/A |
| Xia (2011)[[114](#_ENREF_114)] | China | 6 months | IA | 9 months | 86 | 168 | 168 | 0 | 12-18 | 16.1 |
| Xu (2004)[[115](#_ENREF_115)] | China | 1 month | IA | 14 days | 110 | 21 | 21 | 0 | 50-70 | N/A |
| Ye (2016)[[116](#_ENREF_116)] | China | 12 months | IA | 12 months | 87 | 203 | 104 | 99 | 31-80 | 50.4 |
| Yuan (2017)[[117](#_ENREF_117)] | US | 12 months | IA | 12 months | 152 | 632 | 632 | 0 | 45-80 | 61 |
| Yum (2016)[[118](#_ENREF_118)] | Korea | N/A | IA | 4 weeks | 71 | 153 | 77 | 77 | 12-18 | N/A |
| Zang (2019)[[119](#_ENREF_119)] | China | 3 months | IA | 6 months | 123 | 1623 | 825 | 798 | ≥18 | N/A |
| Zaragoza (2018)[[120](#_ENREF_120)] | Spain | 12 months | IA | 9 months | 137 | 341 | 191 | 150 | ≥ 60 | 72.59 |
| Zhang (2005)[[121](#_ENREF_121)] | China | 5 years | IA | 11.3 weeks | 128 | 41 | 41 | 0 | 24-67 | 46.9 |
| Zhang (2009)[[122](#_ENREF_122)] | China | 12 months | N/A | 12 months | 81 | 61 | 61 | 0 | 24-64 | 44.6 |
| Zhuang (2012)[[123](#_ENREF_123)] | China | 12 months | IA | 9 months | 86 | 207 | 129 | 78 | 30-75 | 51.5 |

AM: administration mode; IA: interview-administration; SA: self- administration;

**Reference**

1. Ahn Y, Kwon E, Shim JE, Park MK, Joo Y, et al. (2007) Validation and reproducibility of food frequency questionnaire for Korean genome epidemiologic study. Eur J Clin Nutr 61: 1435-1441.

2. Hong TK, Dibley MJ, Sibbritt D (2010) Validity and reliability of an FFQ for use with adolescents in Ho Chi Minh City, Vietnam. Public Health Nutr 13: 368-375.

3. Bae YJ, Choi HY, Sung MK, Kim MK, Choi MK (2010) Validity and reproducibility of a food frequency questionnaire to assess dietary nutrients for prevention and management of metabolic syndrome in Korea. Nutr Res Pract 4: 121-127.

4. Barrat E, Aubineau N, Maillot M, Derbord E, Barthes P, et al. (2012) Repeatability and relative validity of a quantitative food-frequency questionnaire among French adults. Food Nutr Res 56.

5. Bjerregaard AA, Tetens I, Olsen SF, Halldorsson TI (2016) Reproducibility of a web-based FFQ for 13- to 15-year-old Danish adolescents. J Nutr Sci 5: e5.

6. Bohlscheid-Thomas S, Hoting I, Boeing H, Wahrendorf J (1997) Reproducibility and relative validity of energy and macronutrient intake of a food frequency questionnaire developed for the German part of the EPIC project. European Prospective Investigation into Cancer and Nutrition. Int J Epidemiol 26 Suppl 1: S71-81.

7. Boucher B, Cotterchio M, Kreiger N, Nadalin V, Block T, et al. (2006) Validity and reliability of the Block98 food-frequency questionnaire in a sample of Canadian women. Public Health Nutr 9: 84-93.

8. Cantin J, Latour E, Ferland-Verry R, Morales Salgado S, Lambert J, et al. (2016) Validity and reproducibility of a food frequency questionnaire focused on the Mediterranean diet for the Quebec population. Nutr Metab Cardiovasc Dis 26: 154-161.

9. M.A. C (2001) Reproducibility and validity of a food frequency questionnaire among women of Japanese ancestry living in Brazil.

10. Collins CE, Boggess MM, Watson JF, Guest M, Duncanson K, et al. (2014) Reproducibility and comparative validity of a food frequency questionnaire for Australian adults. Clin Nutr 33: 906-914.

11. Dehghan M, del Cerro S, Zhang X, Cuneo JM, Linetzky B, et al. (2012) Validation of a semi-quantitative Food Frequency Questionnaire for Argentinean adults. PLoS One 7: e37958.

12. Dehghan M, Ilow R, Zatonska K, Szuba A, Zhang X, et al. (2012) Development, reproducibility and validity of the food frequency questionnaire in the Poland arm of the Prospective Urban and Rural Epidemiological (PURE) study. J Hum Nutr Diet 25: 225-232.

13. Deschamps V, de Lauzon-Guillain B, Lafay L, Borys JM, Charles MA, et al. (2009) Reproducibility and relative validity of a food-frequency questionnaire among French adults and adolescents. Eur J Clin Nutr 63: 282-291.

14. Doustmohammadian A, Amini M (2020) Validity and reliability of a dish-based semi-quantitative food frequency questionnaire for assessment of energy and nutrient intake among Iranian adults. 13: 95.

15. Dumartheray EW, Krieg MA, Cornuz J, Whittamore DR, Lovell DP, et al. (2006) Validation and reproducibility of a semi-quantitative Food Frequency Questionnaire for use in elderly Swiss women. J Hum Nutr Diet 19: 321-330.

16. Egami I, Wakai K, Kato K, Lin Y, Kawamura T, et al. (1999) A simple food frequency questionnaire for Japanese diet--Part II. Reproducibility and validity for nutrient intakes. J Epidemiol 9: 227-234.

17. El Kinany K, Garcia-Larsen V (2018) Adaptation and validation of a food frequency questionnaire (FFQ) to assess dietary intake in Moroccan adults. 17: 61.

18. Elorriaga N, Irazola VE, Defagó MD, Britz M, Martínez-Oakley SP, et al. (2015) Validation of a self-administered FFQ in adults in Argentina, Chile and Uruguay. Public Health Nutr 18: 59-67.

19. Engle A, Lynn LL, Koury K, Boyar AP (1990) Reproducibility and comparability of a computerized, self-administered food frequency questionnaire. Nutr J 13: 281-292.

20. Fallaize R, Forster H (2014) Online dietary intake estimation: reproducibility and validity of the Food4Me food frequency questionnaire against a 4-day weighed food record. 16: e190.

21. Fernandez-Ballart JD, Pinol JL, Zazpe I, Corella D, Carrasco P, et al. (2010) Relative validity of a semi-quantitative food-frequency questionnaire in an elderly Mediterranean population of Spain. Br J Nutr 103: 1808-1816.

22. Field AE, Peterson KE, Gortmaker SL, Cheung L, Rockett H, et al. (1999) Reproducibility and validity of a food frequency questionnaire among fourth to seventh grade inner-city school children: implications of age and day-to-day variation in dietary intake. Public Health Nutr 2: 293-300.

23. Filippi AR, Amodio E, Napoli G, Breda J, Bianco A, et al. (2014) The web-based ASSO-food frequency questionnaire for adolescents: relative and absolute reproducibility assessment. Clin Nutr Res 13: 119.

24. Flagg EW, Coates RJ, Calle EE, Potischman N, Thun MJ (2000) Validation of the American Cancer Society Cancer Prevention Study II Nutrition Survey Cohort Food Frequency Questionnaire. Epidemiology 11: 462-468.

25. Franceschi S, Barbone F, Negri E, Decarli A, Ferraroni M, et al. (1995) Reproducibility of an Italian food frequency questionnaire for cancer studies. Results for specific nutrients. Ann Epidemiol 5: 69-75.

26. Friis S, Kruger Kjaer S, Stripp C, Overvad K (1997) Reproducibility and relative validity of a self-administered semiquantitative food frequency questionnaire applied to younger women. J Clin Epidemiol 50: 303-311.

27. Gilsing A, Mayhew AJ, Payette H, Shatenstein B, Kirkpatrick SI, et al. (2018) Validity and Reliability of a Short Diet Questionnaire to Estimate Dietary Intake in Older Adults in a Subsample of the Canadian Longitudinal Study on Aging. Nutrients 10.

28. Gnardellis C, Trichopoulou A, Katsouyanni K, Polychronopoulos E, Rimm EB, et al. (1995) Reproducibility and validity of an extensive semiquantitative food frequency questionnaire among Greek school teachers. Epidemiology 6: 74-77.

29. Goulet J, Nadeau G, Lapointe A, Lamarche B, Lemieux S (2004) Validity and reproducibility of an interviewer-administered food frequency questionnaire for healthy French-Canadian men and women. Nutr J 3: 13.

30. Hebden L, Kostan E, O'Leary F, Hodge A, Allman-Farinelli M (2013) Validity and reproducibility of a food frequency questionnaire as a measure of recent dietary intake in young adults. PLoS One 8: e75156.

31. Hernandez-Avila M, Romieu I, Parra S, Hernandez-Avila J, Madrigal H, et al. (1998) Validity and reproducibility of a food frequency questionnaire to assess dietary intake of women living in Mexico City. Salud Publica Mex 40: 133-140.

32. Ibiebele TI, Parekh S, Mallitt KA, Hughes MC, O'Rourke PK, et al. (2009) Reproducibility of food and nutrient intake estimates using a semi-quantitative FFQ in Australian adults. Public Health Nutr 12: 2359-2365.

33. Imaeda N, Goto C, Tokudome Y, Hirose K, Tajima K, et al. (2007) Reproducibility of a short food frequency questionnaire for Japanese general population. J Epidemiol 17: 100-107.

34. Iqbal R, Ajayan K, Bharathi AV, Zhang X, Islam S, et al. (2009) Refinement and validation of an FFQ developed to estimate macro- and micronutrient intakes in a south Indian population. Public Health Nutr 12: 12-18.

35. Ishihara J, Sobue T, Yamamoto S, Yoshimi I, Sasaki S, et al. (2003) Validity and reproducibility of a self-administered food frequency questionnaire in the JPHC Study Cohort II: study design, participant profile and results in comparison with Cohort I. J Epidemiol 13: S134-147.

36. Ishihara J, Iwasaki M, Kunieda CM, Hamada GS, Tsugane S (2009) Food frequency questionnaire is a valid tool in the nutritional assessment of Brazilian women of diverse ethnicity. Asia Pac J Clin Nutr 18: 76-80.

37. Jackson MD, Motswagole BS, Kwape LD, Kobue-Lekalake RI, Rakgantswana TB, et al. (2013) Validation and reproducibility of an FFQ for use among adults in Botswana. Public Health Nutr 16: 1995-2004.

38. Jackson M, Walker S, Cade J, Forrester T, Cruickshank JK, et al. (2001) Reproducibility and validity of a quantitative food-frequency questionnaire among Jamaicans of African origin. Public Health Nutr 4: 971-980.

39. Jackson MD, Walker SP, Younger NM, Bennett FI (2011) Use of a food frequency questionnaire to assess diets of Jamaican adults: validation and correlation with biomarkers. Nutr J 10: 28.

40. Esmaillzadeh A, Omidvar N, Abtahi M, Dadkhah-Piraghaj M, Nikooyeh B, et al. (2003) Calibration of the dietary questionnaire for the Canadian Study of Diet, Lifestyle and Health cohort. BMC Res Notes 6: 79-86.

41. Johansson I, Hallmans G, Wikman A, Biessy C, Riboli E, et al. (2002) Validation and calibration of food-frequency questionnaire measurements in the Northern Sweden Health and Disease cohort. Public Health Nutr 5: 487-496.

42. Johansson L, Solvoll K, Opdahl S, Bjørneboe GE, Drevon CA (1997) Response rates with different distribution methods and reward, and reproducibility of a quantitative food frequency questionnaire. Eur J Clin Nutr 51: 346-353.

43. Juliana Araujo Teixeira MLB, Anna R. Giuliano, Regina Mara Fisberg, and Dirce Maria Lobo Marchioni4 (2011) Performance of the quantitative food frequency questionnaire used in the Brazilian center of the prospective study Natural History of Human Papillomavirus Infection in Men: The HIM Study. J Am Diet Assoc 111: 1045-1051.

44. Katsouyanni K, Rimm EB, Gnardellis C, Trichopoulos D, Polychronopoulos E, et al. (1997) Reproducibility and relative validity of an extensive semi-quantitative food frequency questionnaire using dietary records and biochemical markers among Greek schoolteachers. Int J Epidemiol 26 Suppl 1: S118-127.

45. Kesse-Guyot E, Castetbon K, Touvier M, Hercberg S, Galan P (2010) Relative validity and reproducibility of a food frequency questionnaire designed for French adults. Ann Nutr Metab 57: 153-162.

46. Kim DW, Song S, Lee JE, Oh K, Shim J, et al. (2015) Reproducibility and validity of an FFQ developed for the Korea National Health and Nutrition Examination Survey (KNHANES). Public Health Nutr 18: 1369-1377.

47. Kristal AR, Kolar AS, Fisher JL, Plascak JJ, Stumbo PJ, et al. (2014) Evaluation of web-based, self-administered, graphical food frequency questionnaire. J Acad Nutr Diet 114: 613-621.

48. Labonté M, Cyr A, Baril-Gravel L, Royer MM, Lamarche B (2012) Validity and reproducibility of a web-based, self-administered food frequency questionnaire. Eur J Clin Nutr 66: 166-173.

49. Lazarus R, Wilson A, Gliksman M, Aiken J (1995) Repeatability of nutrient intakes estimated by a semiquantitative food frequency questionnaire in elderly subjects. JMIR Hum Factors 5: 65-68.

50. Lee Y, Park K (2016) Reproducibility and validity of a semi-quantitative FFQ for trace elements. Br J Nutr 116: 864-873.

51. Lee MS, Pan WH, Liu KL, Yu MS (2006) Reproducibility and validity of a Chinese food frequency questionnaire used in Taiwan. Asia Pac J Clin Nutr 15: 161-169.

52. Leon Guerrero RT, Chong M, Novotny R, Wilkens LR, Badowski G, et al. (2015) Relative validity and reliability of a quantitative food frequency questionnaire for adults in Guam. Food Nutr Res 59: 26276.

53. Li K, Takezaki T, Lv LW, Yu P, Song FY, et al. (2005) Reproducibility of a semi-quantitative food frequency questionnaire in Chaoshan area, China. Asian Pac J Cancer Prev 6: 521-526.

54. Longnecker MP, Lissner L, Holden JM, Flack VF, Taylor PR, et al. (1993) The reproducibility and validity of a self-administered semiquantitative food frequency questionnaire in subjects from South Dakota and Wyoming. Epidemiology 4: 356-365.

55. Lyu LC, Lin CF, Chang FH, Chen HF, Lo CC, et al. (2007) Meal distribution, relative validity and reproducibility of a meal-based food frequency questionnaire in Taiwan. Asia Pac J Clin Nutr 16: 766-776.

56. Macedo-Ojeda G, Vizmanos-Lamotte B, Marquez-Sandoval YF, Rodriguez-Rocha NP, Lopez-Uriarte PJ, et al. (2013) Validation of a semi-quantitative food frequency questionnaire to assess food groups and nutrient intake. Nutr Hosp 28: 2212-2220.

57. MacIntyre UE, Venter CS, Vorster HH (2001) A culture-sensitive quantitative food frequency questionnaire used in an African population: 1. Development and reproducibility. Public Health Nutr 4: 53-62.

58. Malekshah AF, Kimiagar M, Saadatian-Elahi M, Pourshams A, Nouraie M, et al. (2006) Validity and reliability of a new food frequency questionnaire compared to 24 h recalls and biochemical measurements: pilot phase of Golestan cohort study of esophageal cancer. Eur J Clin Nutr 60: 971-977.

59. Mannisto S, Virtanen M, Mikkonen T, Pietinen P (1996) Reproducibility and validity of a food frequency questionnaire in a case-control study on breast cancer. J Clin Epidemiol 49: 401-409.

60. Marchioni DM, Voci SM, Lima FE, Fisberg RM, Slater B (2007) Reproducibility of a food frequency questionnaire for adolescents. Cad Saude Publica 23: 2187-2196.

61. María Elisa Zapataa, Romina Buffarinib, Nadia Lingiardia, Ana Luiza Gonçalves-Soaresb (2015) Reproducibility and relative validity of a semi-quantitative food-frequency questionnaire in an adult population of Rosario, Argentina.

62. Marques-Vidal P, Ross A, Wynn E, Rezzi S, Paccaud F, et al. (2011) Reproducibility and relative validity of a food-frequency questionnaire for French-speaking Swiss adults. Food Nutr Res 55.

63. Marshall SJ, Livingstone KM, Celis-Morales C, Forster H, Fallaize R, et al. (2016) Reproducibility of the Online Food4Me Food-Frequency Questionnaire for Estimating Dietary Intakes across Europe. J Nutr 146: 1068-1075.

64. Martinez MF, Philippi ST, Estima C, Leal G (2013) Validity and reproducibility of a food frequency questionnaire to assess food group intake in adolescents. Cad Saude Publica 29: 1795-1804.

65. Martin-Moreno JM, Boyle P, Gorgojo L, Maisonneuve P, Fernandez-Rodriguez JC, et al. (1993) Development and validation of a food frequency questionnaire in Spain. Int J Epidemiol 22: 512-519.

66. Maruyama K, Kokubo Y, Yamanaka T, Watanabe M, Iso H, et al. (2015) The reasonable reliability of a self-administered food frequency questionnaire for an urban, Japanese, middle-aged population: the Suita study. Nutr Res 35: 14-22.

67. Marventano S, Mistretta A, Platania A, Galvano F, Grosso G (2016) Reliability and relative validity of a food frequency questionnaire for Italian adults living in Sicily, Southern Italy. Int J Food Sci Nutr 67: 857-864.

68. Maryam Nouri SG, Seyed Amir Reza Mohajeri (2017) Relative Validity and Reproducibility of a Semi-quantitative Food Frequency Questionnaire among Urban Iranians. Int J Health Life Sci.

69. McKeown NM, Day NE, Welch AA, Runswick SA, Luben RN, et al. (2001) Use of biological markers to validate self-reported dietary intake in a random sample of the European Prospective Investigation into Cancer United Kingdom Norfolk cohort. Am J Clin Nutr 74: 188-196.

70. McPherson RS, Kohl HW, 3rd, Garcia G, Nichaman MZ, Hanis CL (1995) Food-frequency questionnaire validation among Mexican-Americans: Starr County, Texas. Ann Epidemiol 5: 378-385.

71. Messerer M, Johansson SE, Wolk A (2004) The validity of questionnaire-based micronutrient intake estimates is increased by including dietary supplement use in Swedish men. J Nutr 134: 1800-1805.

72. Tijerina A, Tur JA (2020) Development and Validation of a Semiquantitative Food Frequency Questionnaire to Assess Dietary Intake in 40-65-Year-Old Mexican Women. Ann Nutr Metab 76: 73-82.

73. Mirmiran P, Esfahani FH, Mehrabi Y, Hedayati M, Azizi F (2010) Reliability and relative validity of an FFQ for nutrients in the Tehran lipid and glucose study. Public Health Nutr 13: 654-662.

74. Morris MC, Tangney CC, Bienias JL, Evans DA, Wilson RS (2003) Validity and reproducibility of a food frequency questionnaire by cognition in an older biracial sample. Am J Epidemiol 158: 1213-1217.

75. Munger RG, Folsom AR, Kushi LH, Kaye SA, Sellers TA (1992) Dietary assessment of older Iowa women with a food frequency questionnaire: nutrient intake, reproducibility, and comparison with 24-hour dietary recall interviews. Am J Epidemiol 136: 192-200.

76. Nelida Nelida Fornes MLFS, and Berenice Muller Elias (2003) Reproducibility and validity of a food-frequency questionnaire for use among low-income Brazilian workers. Public Health Nutr 6: 821-827.

77. Ocke MC, Bueno-de-Mesquita HB, Pols MA, Smit HA, van Staveren WA, et al. (1997) The Dutch EPIC food frequency questionnaire. II. Relative validity and reproducibility for nutrients. Int J Epidemiol 26 Suppl 1: S49-58.

78. Ogawa K, Tsubono Y, Nishino Y, Watanabe Y, Ohkubo T, et al. (2003) Validation of a food-frequency questionnaire for cohort studies in rural Japan. Public Health Nutr 6: 147-157.

79. Overby NC, Johannesen E, Jensen G, Skjaevesland AK, Haugen M (2014) Test-retest reliability and validity of a web-based food-frequency questionnaire for adolescents aged 13-14 to be used in the Norwegian Mother and Child Cohort Study (MoBa). Food Nutr Res 58.

80. Palacios C, Trak MA, Betancourt J, Joshipura K, Tucker KL (2015) Validation and reproducibility of a semi-quantitative FFQ as a measure of dietary intake in adults from Puerto Rico. Public Health Nutr 18: 2550-2558.

81. Park MK, Noh HY, Song NY, Paik HY, Park S, et al. (2012) Validity and reliability of a dish-based, semi-quantitative food frequency questionnaire for Korean diet and cancer research. Asian Pac J Cancer Prev 13: 545-552.

82. Preston AM, Palacios C, Rodriguez CA, Velez-Rodriguez RM (2011) Validation and reproducibility of a semi-quantitative food frequency questionnaire for use in Puerto Rican children. P R Health Sci J 30: 58-64.

83. Riboli E, Toniolo P, Kaaks R, Shore RE, Casagrande C, et al. (1997) Reproducibility of a food frequency questionnaire used in the New York University Women's Health Study: effect of self-selection by study subjects. Eur J Clin Nutr 51: 437-442.

84. Rifas-Shiman SL, Willett WC, Lobb R, Kotch J, Dart C, et al. (2001) PrimeScreen, a brief dietary screening tool: reproducibility and comparability with both a longer food frequency questionnaire and biomarkers. Public Health Nutr 4: 249-254.

85. Rimm EB, Giovannucci EL, Stampfer MJ, Colditz GA, Litin LB, et al. (1992) Reproducibility and validity of an expanded self-administered semiquantitative food frequency questionnaire among male health professionals. Am J Epidemiol 135: 1114-1126; discussion 1127-1136.

86. Rockett HR, Wolf AM, Colditz GA (1995) Development and reproducibility of a food frequency questionnaire to assess diets of older children and adolescents. J Am Diet Assoc 95: 336-340.

87. Rodriguez CA, Smith ER, Villamor E, Zavaleta N, Respicio-Torres G, et al. (2017) Development and Validation of a Food Frequency Questionnaire to Estimate Intake among Children and Adolescents in Urban Peru. Nutrients 9.

88. Salvesen L, Hillesund ER, Vik FN, Brantsæter AL (2019) Reproducibility and relative validity of a newly developed web-based food-frequency questionnaire for assessment of preconception diet. 5: 47.

89. Sam CH, Skeaff S, Skidmore PM (2014) A comprehensive FFQ developed for use in New Zealand adults: reliability and validity for nutrient intakes. Public Health Nutr 17: 287-296.

90. Sam CHY, Skidmore P, Skeaff S, Wall C, Bradbury KE, et al. (2020) Relative Validity and Reproducibility of a Short FoodFrequency Questionnaire to Assess Nutrient Intakesof New Zealand Adults. Nutrients 12.

91. Sanjeevi N, Freeland-Graves J, George GC (2017) Relative Validity and Reliability of a 1-Week, Semiquantitative Food Frequency Questionnaire for Women Participating in the Supplemental Nutrition Assistance Program. J Acad Nutr Diet 117: 1972-1982 e1972.

92. Sasaki S, Ishihara J, Tsugane S (2003) Reproducibility of a self-administered food frequency questionnaire used in the 5-year follow-up survey of the JPHC Study Cohort I to assess food and nutrient intake. J Epidemiol 13: S115-124.

93. Schroder H, Covas MI, Marrugat J, Vila J, Pena A, et al. (2001) Use of a three-day estimated food record, a 72-hour recall and a food-frequency questionnaire for dietary assessment in a Mediterranean Spanish population. Clin Nutr 20: 429-437.

94. Selem SS, Carvalho AM, Verly-Junior E, Carlos JV, Teixeira JA, et al. (2014) Validity and reproducibility of a food frequency questionnaire for adults of Sao Paulo, Brazil. Rev Bras Epidemiol 17: 852-859.

95. Shai I, Rosner BA, Shahar DR, Vardi H, Azrad AB, et al. (2005) Dietary evaluation and attenuation of relative risk: multiple comparisons between blood and urinary biomarkers, food frequency, and 24-hour recall questionnaires: the DEARR study. J Nutr 135: 573-579.

96. Shimizu H, Ohwaki A, Kurisu Y, Takatsuka N, Ido M, et al. (1999) Validity and reproducibility of a quantitative food frequency questionnaire for a cohort study in Japan. Jpn J Clin Oncol 29: 38-44.

97. Shu XO, Yang G, Jin F, Liu D, Kushi L, et al. (2004) Validity and reproducibility of the food frequency questionnaire used in the Shanghai Women's Health Study. Eur J Clin Nutr 58: 17-23.

98. (!!! INVALID CITATION !!!).

99. Smith W, Mitchell P, Reay EM, Webb K, Harvey PW (1998) Validity and reproducibility of a self-administered food frequency questionnaire in older people. Aust N Z J Public Health 22: 456-463.

100. Sudha V, Radhika G, Sathya RM, Ganesan A, Mohan V (2006) Reproducibility and validity of an interviewer-administered semi-quantitative food frequency questionnaire to assess dietary intake of urban adults in southern India. Int J Food Sci Nutr 57: 481-493.

101. Takachi R, Ishihara J, Iwasaki M, Hosoi S, Ishii Y, et al. (2011) Validity of a self-administered food frequency questionnaire for middle-aged urban cancer screenees: comparison with 4-day weighed dietary records. J Epidemiol 21: 447-458.

102. Tang Y, Liu Y, Xu L, Jia Y, Shan D, et al. (2015) Validity and reproducibility of a revised semi-quantitative food frequency questionnaire (SQFFQ) for women of age-group 12-44 years in Chengdu. J Health Popul Nutr 33: 50-59.

103. Tavani A, Negri E, Ferraroni M, D'Avanzo B, Decarli A, et al. (1995) Influence of some covariates on the reproducibility of an Italian semi-quantitative food frequency questionnaire. Eur J Cancer Prev 4: 319-327.

104. Tollosa DN, Van Camp J, Huybrechts I, Huybregts L, Van Loco J, et al. (2017) Validity and Reproducibility of a Food Frequency Questionnaire for Dietary Factors Related to Colorectal Cancer. Int J Environ Res Public Health 9.

105. Tsubono Y, Kobayashi M, Sasaki S, Tsugane S (2003) Validity and reproducibility of a self-administered food frequency questionnaire used in the baseline survey of the JPHC Study Cohort I. J Epidemiol 13: S125-133.

106. Tsubono Y, Ogawa K, Watanabe Y, Nishino Y, Tsuji I, et al. (2001) Food frequency questionnaire and a screening test. Nutr Cancer 39: 78-84.

107. Turconi G, Bazzano R, Roggi C, Cena H (2010) Reliability and relative validity of a quantitative food-frequency questionnaire for use among adults in Italian population. Int J Food Sci Nutr 61: 846-862.

108. Villegas R, Yang G, Liu D, Xiang YB, Cai H, et al. (2007) Validity and reproducibility of the food-frequency questionnaire used in the Shanghai men's health study. Br J Nutr 97: 993-1000.

109. Vereecken CA, De Bourdeaudhuij I, Maes L (2010) The HELENA online food frequency questionnaire: reproducibility and comparison with four 24-h recalls in Belgian-Flemish adolescents. Eur J Clin Nutr 64: 541-548.

110. Watanabe M, Yamaoka K, Yokotsuka M, Adachi M, Tango T (2011) Validity and reproducibility of the FFQ (FFQW82) for dietary assessment in female adolescents. Public Health Nutr 14: 297-305.

111. Watson JF, Collins CE, Sibbritt DW, Dibley MJ, Garg ML (2009) Reproducibility and comparative validity of a food frequency questionnaire for Australian children and adolescents. Int J Behav Nutr Phys Act 6: 62.

112. Whitton C, Ho JCY, Tay Z, Rebello SA, Lu Y, et al. (2017) Relative Validity and Reproducibility of a Food Frequency Questionnaire for Assessing Dietary Intakes in a Multi-Ethnic Asian Population Using 24-h Dietary Recalls and Biomarkers. Nutrients 9.

113. Willett WC, Sampson L, Stampfer MJ, Rosner B, Bain C, et al. (1985) Reproducibility and validity of a semiquantitative food frequency questionnaire. Am J Epidemiol 122: 51-65.

114. Xia W, Sun C, Zhang L, Zhang X, Wang J, et al. (2011) Reproducibility and relative validity of a food frequency questionnaire developed for female adolescents in Suihua, North China. J Nutr Gerontol Geriatr 6: e19656.

115. Xu L, M JD, D'Este C (2004) Reliability and validity of a food-frequency questionnaire for Chinese postmenopausal women. Public Health Nutr 7: 91-98.

116. Ye Q, Hong X, Wang Z, Yang H, Chen X, et al. (2016) Reproducibility and validity of an FFQ developed for adults in Nanjing, China. Br J Nutr 115: 887-894.

117. Yuan C, Spiegelman D, Rimm EB, Rosner BA, Stampfer MJ, et al. (2017) Validity of a Dietary Questionnaire Assessed by Comparison With Multiple Weighed Dietary Records or 24-Hour Recalls. Am J Epidemiol 185: 570-584.

118. Yum J, Lee S (2016) Development and evaluation of a dish-based semiquantitative food frequency questionnaire for Korean adolescents. Nutr Res Pract 10: 433-441.

119. Zang J, Luo B, Chang S, Jin S, Shan C, et al. (2019) Validity and reliability of a food frequency questionnaire for assessing dietary intake among Shanghai residents. 18: 30.

120. Zaragoza-Marti A, Ferrer-Cascales R, Hurtado-Sanchez JA, Laguna-Perez A, Cabanero-Martinez MJ (2018) Cross-Cultural Adaptation, Validity, and Reproducibility of the Mediterranean Islands Study Food Frequency Questionnaire in the Elderly Population Living in the Spanish Mediterranean. Nutrients 10.

121. Zhang M, Binns CW, Lee AH (2005) A quantitative food frequency questionnaire for women in southeast China: development and reproducibility. Nutr Diet 17: 29-35.

122. Zhang CX, Ho SC (2009) Validity and reproducibility of a food frequency Questionnaire among Chinese women in Guangdong province. Asia Pac J Clin Nutr 18: 240-250.

123. Zhuang M, Yuan Z, Lin L, Hu B, Wang X, et al. (2012) Reproducibility and relative validity of a food frequency questionnaire developed for adults in Taizhou, China. PLoS One 7: e48341.
