# Supplementary material for: A meta-analysis of the reproducibility of food frequency questionnaires in nutritional epidemiological studies
Source: Int J Behav Nutr Phys Act. 2021 Jan 11;18:12. doi: 10.1186/s12966-020-01078-4 (PMC7802360; doi:10.1186/s12966-020-01078-4)
Supplement: Supplementary file 3 — Additional file 3 Supplemental Table 2. Pooled intraclass correlation coefficients for energy and nutrients stratified by age. [file 12966_2020_1078_MOESM3_ESM.docx]

**Supplemental Table 2. Pooled** **intraclass correlation coefficients for energy and nutrients stratified by age ***

| Nutrient | Adult (18-50) | | | | | | the elderly (> 50) | | | | | | adolescent (< 18) | | | | | |
| --- | --- | --- | --- | --- | --- | --- | --- | --- | --- | --- | --- | --- | --- | --- | --- | --- | --- | --- |
|  | Crude | | | Energy-adjusted | | | Crude | | | Energy-adjusted | | | Crude | | | Energy-adjusted | | |
|  | ICC (95% CI) | N | *I^2^* | ICC (95% CI) | N | *I^2^* | ICC (95% CI) | N | *I^2^* | ICC (95% CI) | N | *I^2^* | ICC (95% CI) | N | *I^2^* | ICC (95% CI) | N | *I^2^* |
| Energy | 0.713 (0.673, 0.750) | 38 | 84.3 | N/A | N/A | N/A | 0.698 (0.553, 0.802) | 20 | 98.5 | N/A | N/A | N/A | 0.646 (0.530, 0.738) | 3 | 44.2 | N/A | N/A | N/A |
| Protein | 0.671 (0.630, 0.708) | 36 | 80.2 | 0.621 (0.549, 0.684) | 13 | 66.6 | 0.626 (0.554, 0.690) | 22 | 93.1 | 0.615 (0.525, 0.692) | 9 | 87.5 | 0.549 (0.457, 0.629) | 5 | 50.1 | 0.447 (0.355, 0.529) | 3 | N/A |
| Fat | 0.675 (0.623, 0.722) | 32 | 87.6 | 0.584 (0.473, 0.677) | 10 | 78.4 | 0.623 (0.544, 0.691) | 18 | 92.8 | 0.614 (0.486, 0.716) | 6 | 91.4 | 0.469 (0.371, 0.558) | 5 | 46.8 | 0.371 (0.274, 0.461) | 3 | N/A |
| Plant fat | 0.616 (0.494, 0.714) | 3 | 60.4 | N/A | N/A | N/A | 0.482 (0.331, 0.608) | 2 | N/A | N/A | N/A | N/A | N/A | N/A | N/A | N/A | N/A | N/A |
| Animal fat | N/A | N/A | N/A | N/A | N/A | N/A | N/A | N/A | N/A | N/A | N/A | N/A | N/A | N/A | N/A | N/A | N/A | N/A |
| MUFA | 0.642 (0.594, 0.685) | 26 | 69.1 | 0.618 (0.527, 0.695) | 12 | 69.4 | 0.660 (0.606, 0.707) | 13 | 84.8 | 0.680 (0.556, 0.774) | 5 | 92.3 | 0.499 (0.241, 0.691) | 2 | 84.7 | 0.430 (0.265, 0.570) | 1 | N/A |
| PUFA | 0.639 (0.592, 0.682) | 28 | 70.9 | 0.527 (0.422, 0.618) | 12 | 69 | 0.659 (0.519, 0.765) | 15 | 97.7 | 0.670 (0.549, 0.763) | 5 | 91.6 | 0.457 (0.080, 0.719) | 2 | 91.8 | 0.430 (0.265, 0.570) | 1 | N/A |
| n-3 PUFA | N/A | N/A | N/A | N/A | N/A | N/A | N/A | N/A | N/A | N/A | N/A | N/A | N/A | N/A | N/A | N/A | N/A | N/A |
| n-6 PUFA | N/A | N/A | N/A | N/A | N/A | N/A | N/A | N/A | N/A | N/A | N/A | N/A | N/A | N/A | N/A | N/A | N/A | N/A |
| SFA | 0.678 (0.629, 0.722) | 32 | 80.1 | 0.601 (0.504, 0.684) | 13 | 74.8 | 0.711 (0.540, 0.825) | 15 | 98.7 | 0.731 (0.620, 0.814) | 5 | 92.7 | 0.542 (0.438, 0.633) | 2 | 29.5 | 0.480 (0.323, 0.611) | 1 | N/A |
| Linoleic acid | 0.750 (0.628, 0.835) | 1 | N/A | 0.679 (0.533, 0.786) | 1 | N/A | 0.727 (0.615, 0.809) | 3 | 80.2 | 0.689 (0.551, 0.790) | 2 | 85.2 | 0.290 (0.152, 0.416) | 1 | N/A | N/A | N/A | N/A |
| Linolenic acid | 0.730 (0.600, 0.822) | 1 | N/A | N/A | N/A | N/A | 0.695 (0.654, 0.731) | 2 | N/A | N/A | N/A | N/A | 0.420 (0.294, 0.531) | 1 | N/A | N/A | N/A | N/A |
| EPA | N/A | N/A | N/A | N/A | N/A | N/A | N/A | N/A | N/A | N/A | N/A | N/A | N/A | N/A | N/A | N/A | N/A | N/A |
| DHA | N/A | N/A | N/A | N/A | N/A | N/A | N/A | N/A | N/A | N/A | N/A | N/A | N/A | N/A | N/A | N/A | N/A | N/A |
| Trans-fat | 0.573 (0.310, 0.754) | 2 | 82.5 | N/A | N/A | N/A | 0.646 (0.301, 0.841) | 2 | 88 | N/A | N/A | N/A | N/A | N/A | N/A | N/A | N/A | N/A |
| Cholesterol | 0.701 (0.657, 0.741) | 27 | 75.2 | 0.644 (0.571, 0.706) | 14 | 66.9 | 0.603 (0.509, 0.683) | 18 | 94.7 | 0.593 (0.481, 0.686) | 9 | 91.3 | 0.535 (0.158, 0.776) | 3 | 93.3 | 0.542 (0.423, 0.643) | 2 | N/A |
| Lipid | 0.709 (0.596, 0.795) | 1 | N/A | 0.499 (0.336, 0.633) | 1 | N/A | 0.696 (0.320, 0.882) | 3 | 95.1 | 0.707 (0.410, 0.869) | 3 | 93.2 | N/A | N/A | N/A | N/A | N/A | N/A |
| Carbohydrate | 0.694 (0.637, 0.743) | 31 | 90.4 | 0.694 (0.637, 0.743) | 31 | 90.4 | 0.690 (0.567, 0.782) | 25 | 98.1 | 0.690 (0.567, 0.782) | 25 | 98.1 | 0.524 (0.382, 0.642) | 6 | 78.4 | 0.524 (0.382, 0.642) | 6 | 78.4 |
| Sucrose | 0.666 (0.575, 0.740) | 3 | 59.6 | N/A | N/A | N/A | N/A | N/A | N/A | N/A | N/A | N/A | 0.529 (0.418, 0.625) | 1 | N/A | N/A | N/A | N/A |
| Sugar | 0.749 (0.628, 0.834) | 5 | 74.8 | N/A | N/A | N/A | 0.723 (0.681, 0.759) | 2 | 4.1 | N/A | N/A | N/A | 0.510 (0.396, 0.608) | 1 | N/A | N/A | N/A | N/A |
| Starch | 0.510 (0.351, 0.640) | 1 | N/A | N/A | N/A | N/A | 0.690 (0.536, 0.799) | 1 | N/A | N/A | N/A | N/A | 0.310 (0.174, 0.434) | 1 | N/A | N/A | N/A | N/A |
| Fiber | 0.688 (0.636, 0.734) | 32 | 84.8 | 0.720 (0.636, 0.788) | 9 | 77.3 | 0.704 (0.632, 0.763) | 18 | 94.5 | 0.677 (0.560, 0.768) | 9 | 94 | 0.503 (0.435, 0.565) | 4 | N/A | 0.451 (0.234, 0.624) | 3 | 78.2 |
| Soluble fiber | N/A | N/A | N/A | N/A | N/A | N/A | N/A | N/A | N/A | N/A | N/A | N/A | N/A | N/A | N/A | N/A | N/A | N/A |
| Insoluble fiber | N/A | N/A | N/A | N/A | N/A | N/A | N/A | N/A | N/A | N/A | N/A | N/A | N/A | N/A | N/A | N/A | N/A | N/A |
| Alcohol | 0.793 (0.742, 0.835) | 18 | 81.1 | 0.770 (0.727, 0.807) | 7 | 2.3 | 0.866 (0.728, 0.937) | 3 | 91.4 | 0.884 (0.811, 0.930) | 2 | 92.4 | 0.730 (0.655, 0.790) | 1 | N/A | N/A | N/A | N/A |
| Vitamin A | 0.656 (0.552, 0.739) | 16 | 93.8 | 0.673 (0.524, 0.782) | 5 | 84.6 | 0.596 (0.451, 0.709) | 9 | 95.8 | 0.565 (0.333, 0.732) | 6 | 94.5 | 0.472 (0.379, 0.556) | 2 | N/A | 0.349 (0.176, 0.502) | 1 | N/A |
| Retinol | 0.576 (0.470, 0.665) | 11 | 86.5 | 0.575 (0.365, 0.729) | 5 | 77 | 0.620 (0.511, 0.709) | 5 | 67.3 | 0.539 (0.411, 0.646) | 2 | 45.5 | 0.605 (0.513, 0.682) | 2 | N/A | 0.414 (0.214, 0.581) | 2 | 52.7 |
| Carotene | 0.588 (0.407, 0.724) | 2 | 88.3 | N/A | N/A | N/A | 0.644 (0.504, 0.750) | 7 | 95.5 | 0.512 (0.328, 0.658) | 5 | 86.2 | N/A | N/A | N/A | N/A | N/A | N/A |
| β-Carotene | 0.667 (0.599, 0.726) | 15 | 75.7 | 0.596 (0.335, 0.773) | 5 | 85.2 | 0.704 (0.661, 0.743) | 4 | 36.4 | 0.660 (0.613, 0.701) | 1 | N/A | N/A | N/A | N/A | N/A | N/A | N/A |
| Vitamin C | 0.654 (0.593, 0.707) | 30 | 89.6 | 0.626 (0.507, 0.722) | 13 | 87.7 | 0.726 (0.603, 0.816) | 13 | 97.9 | 0.732 (0.539, 0.852) | 6 | 97.6 | 0.497 (0.368, 0.608) | 4 | 67.6 | 0.379 (0.214, 0.524) | 3 | 60 |
| Vitamin D | 0.570 (0.469, 0.655) | 4 | 71.5 | N/A | N/A | N/A | 0.731 (0.558, 0.842) | 10 | 98.7 | 0.709 (0.404, 0.872) | 4 | 98.5 | 0.575 (0.493, 0.646) | 2 | N/A | 0.469 (0.311, 0.603) | 1 | N/A |
| Vitamin E | 0.636 (0.547, 0.710) | 18 | 92.3 | 0.572 (0.353, 0.731) | 5 | 88.8 | 0.720 (0.570, 0.823) | 13 | 98.4 | 0.669 (0.519, 0.779) | 8 | 95.4 | 0.542 (0.474, 0.603) | 3 | N/A | 0.370 (0.264, 0.467) | 2 | N/A |
| Vitamin K | 0.573 (0.176, 0.809) | 2 | 93.6 | N/A | N/A | N/A | 0.712 (0.673, 0.747) | 2 | N/A | N/A | N/A | N/A | N/A | N/A | N/A | N/A | N/A | N/A |
| Thiamin | 0.664 (0.599, 0.719) | 16 | 85.8 | 0.629 (0.443, 0.763) | 5 | 88 | 0.610 (0.538, 0.674) | 12 | 89.5 | 0.635 (0.442, 0.771) | 5 | 96.4 | 0.534 (0.466, 0.596) | 3 | N/A | 0.454 (0.355, 0.542) | 2 | N/A |
| Riboflavin | 0.648 (0.606, 0.686) | 14 | 59.4 | 0.564 (0.397, 0.695) | 3 | 69.8 | 0.695 (0.617, 0.759) | 12 | 93.5 | 0.668 (0.493, 0.790) | 6 | 96.2 | 0.615 (0.255, 0.825) | 2 | 94.8 | 0.439 (0.310, 0.553) | 1 | N/A |
| Niacin | 0.682 (0.599, 0.750) | 14 | 90 | 0.634 (0.515, 0.730) | 3 | 55.3 | 0.682 (0.570, 0.769) | 5 | 88.7 | 0.649 (0.492, 0.764) | 5 | 94.4 | 0.581 (0.480, 0.666) | 3 | 57 | 0.424 (0.323, 0.516) | 2 | N/A |
| Vitamin B6 | 0.678 (0.566, 0.765) | 6 | 81.8 | 0.526 (0.400, 0.633) | 2 | 18 | 0.786 (0.418, 0.932) | 6 | 99.2 | 0.797 (0.581, 0.908) | 3 | 97.8 | 0.459 (0.339, 0.565) | 1 | N/A | N/A | N/A | N/A |
| Folate | 0.682 (0.620, 0.735) | 15 | 84.2 | 0.598 (0.443, 0.719) | 5 | 81.1 | 0.563 (0.436, 0.668) | 9 | 94.6 | 0.599 (0.547, 0.647) | 1 | N/A | 0.519 (0.406, 0.616) | 1 | N/A | N/A | N/A | N/A |
| Vitamin B12 | 0.671 (0.567, 0.755) | 6 | 80.3 | 0.656 (0.492, 0.774) | 3 | 76.1 | 0.712 (0.377, 0.882) | 6 | 98.9 | 0.702 (0.411, 0.863) | 4 | 98.3 | 0.459 (0.339, 0.565) | 1 | N/A | N/A | N/A | N/A |
| Se | 0.676 (0.620, 0.726) | 9 | 70.3 | 0.619 (0.422, 0.761) | 3 | 82.2 | 0.549 (0.320, 0.718) | 1 | N/A | N/A | N/A | N/A | 0.579 (0.442, 0.691) | 1 | N/A | 0.480 (0.323, 0.611) | N/A | N/A |
| Mg | 0.701 (0.623, 0.765) | 12 | 88.4 | 0.648 (0.521, 0.747) | 3 | 64.5 | 0.658 (0.503, 0.773) | 6 | 90.1 | 0.586 (0.338, 0.758) | 3 | 94.1 | 0.450 (0.328, 0.557) | 1 | N/A | N/A | N/A | N/A |
| Ca | 0.670 (0.629, 0.708) | 28 | 78.1 | 0.693 (0.601, 0.767) | 12 | 84.8 | 0.605 (0.505, 0.688) | 20 | 95.8 | 0.616 (0.464, 0.733) | 8 | 94.8 | 0.518 (0.426, 0.600) | 4 | 43.1 | 0.481 (0.393, 0.560) | 3 | N/A |
| Fe | 0.660 (0.598, 0.713) | 24 | 87.5 | 0.625 (0.553, 0.687) | 12 | 61.6 | 0.631 (0.510, 0.727) | 11 | 96.5 | 0.498 (0.343, 0.626) | 4 | 89.9 | 0.531 (0.393, 0.645) | 4 | 73.5 | 0.432 (0.340, 0.516) | 3 | N/A |
| I | N/A | N/A | N/A | N/A | N/A | N/A | N/A | N/A | N/A | N/A | N/A | N/A | N/A | N/A | N/A | N/A | N/A | N/A |
| Zn | 0.615 (0.566, 0.660) | 20 | 71.4 | 0.623 (0.523, 0.705) | 8 | 64.4 | 0.545 (0.466, 0.616) | 4 | 63.3 | 0.589 (0.534, 0.638) | 2 | 5.1 | 0.549 (0.462, 0.626) | 2 | N/A | 0.394 (0.290, 0.488) | 2 | N/A |
| Cu | 0.676 (0.610, 0.732) | 3 | N/A | N/A | N/A | N/A | 0.649 (0.602, 0.692) | 1 | N/A | N/A | N/A | N/A | N/A | N/A | N/A | N/A | N/A | N/A |
| K | 0.712 (0.636, 0.774) | 14 | 90.1 | 0.676 (0.494, 0.800) | 4 | 87.1 | 0.612 (0.468, 0.724) | 11 | 96.7 | 0.585 (0.279, 0.783) | 3 | 96.9 | N/A | N/A | N/A | N/A | N/A | N/A |
| P | 0.658 (0.544, 0.749) | 13 | 91.9 | 0.684 (0.624, 0.737) | 6 | N/A | 0.533 (0.373, 0.662) | 9 | 91.5 | 0.552 (0.334, 0.714) | 3 | 94 | 0.489 (0.373, 0.591) | 1 | N/A | N/A | N/A | N/A |
| Na | 0.666 (0.608, 0.718) | 14 | 79.8 | 0.692 (0.485, 0.825) | 4 | 90.3 | 0.633 (0.149, 0.872) | 10 | 99.2 | 0.674 (0.218, 0.888) | 3 | 99 | 0.599 (0.466, 0.706) | 1 | N/A | 0.549 (0.406, 0.667) | 1 | N/A |
| Mn | N/A | N/A | N/A | N/A | N/A | N/A | N/A | N/A | N/A | N/A | N/A | N/A | N/A | N/A | N/A | N/A | N/A | N/A |

* CI, confidence interval; N/A: not available
