# Supplementary material for: A meta-analysis of the reproducibility of food frequency questionnaires in nutritional epidemiological studies
Source: Int J Behav Nutr Phys Act. 2021 Jan 11;18:12. doi: 10.1186/s12966-020-01078-4 (PMC7802360; doi:10.1186/s12966-020-01078-4)
Supplement: Supplementary file 4 — Additional file 4 Supplemental Table 3. Pooled spearman correlation coefficients for energy and nutrients stratified by age. [file 12966_2020_1078_MOESM4_ESM.docx]

**Supplemental Table 3**. Pooled spearman correlation coefficients for energy and nutrients stratified by age*

| Nutrient | Adult (18-50) | | | | | | the elderly (> 50) | | | | | | adolescent (< 18) | | | | | |
| --- | --- | --- | --- | --- | --- | --- | --- | --- | --- | --- | --- | --- | --- | --- | --- | --- | --- | --- |
|  | Crude | | | Energy-adjusted | | | Crude | | | Energy-adjusted | | | Crude | | | Energy-adjusted | | |
|  | SCC (95% CI) | N | *I^2^* | SCC (95% CI) | N | *I^2^* | SCC (95% CI) | N | *I^2^* | SCC (95% CI) | N | *I^2^* | SCC (95% CI) | N | *I^2^* | SCC (95% CI) | N | *I^2^* |
| Energy | 0.673 (0.642, 0.703) | 62 | 84.8 | N/A | N/A | N/A | 0.648 (0.609, 0.683) | 33 | 78.9 | N/A | N/A | N/A | 0.500 (0.353, 0.623) | 12 | 87.7 | N/A | N/A | N/A |
| Protein | 0.639 (0.610, 0.666) | 62 | 78.8 | 0.575 (0.524, 0.622) | 41 | 85.2 | 0.601 (0.567, 0.634) | 33 | 68.3 | 0.550 (0.505, 0.592) | 19 | 48 | 0.456 (0.287, 0.597) | 12 | 90.3 | 0.426 (0.357, 0.492) | 4 | N/A |
| Fat | 0.648 (0.621, 0.673) | 60 | 75.2 | 0.555 (0.509, 0.599) | 37 | 80.2 | 0.624 (0.588, 0.657) | 32 | 72.7 | 0.579 (0.497, 0.652) | 17 | 83.7 | 0.465 (0.341, 0.572) | 13 | 83.3 | 0.353 (0.230, 0.466) | 2 | N/A |
| Plant fat | 0.550 (0.459, 0.629) | 6 | 63.6 | N/A | N/A | N/A | 0.492 (0.390, 0.582) | 1 | NA | N/A | N/A | N/A | N/A | N/A | N/A | N/A | N/A | N/A |
| Animal fat | 0.698 (0.663, 0.731) | 4 | N/A | N/A | N/A | N/A | 0.683 (0.609, 0.745) | 1 | NA | N/A | N/A | N/A | N/A | N/A | N/A | N/A | N/A | N/A |
| MUFA | 0.639 (0.599, 0.677) | 38 | 84.3 | 0.639 (0.599, 0.677) | 30 | 69.5 | 0.586 (0.544, 0.624) | 20 | 98.5 | 0.586 (0.544, 0.624) | 17 | 61.1 | 0.503 (0.379, 0.610) | 3 | 44.2 | 0.503 (0.379, 0.610) | 3 | 36.7 |
| PUFA | 0.630 (0.593, 0.664) | 34 | 70.1 | 0.527 (0.456, 0.590) | 22 | 82.5 | 0.565 (0.525, 0.602) | 21 | 64 | 0.539 (0.458, 0.612) | 7 | 61.1 | 0.401 (0.123, 0.621) | 3 | 83.2 | 0.383 (0.253, 0.500) | 2 | 13.4 |
| n-3 PUFA | 0.647 (0.572, 0.711) | 5 | 56 | 0.477 (0.402, 0.546) | 4 | 27.8 | 0.575 (0.539, 0.610) | 2 | 24 | 0.449 (0.245, 0.615) | 1 | 75.7 | N/A | N/A | N/A | N/A | N/A | N/A |
| n-6 PUFA | 0.590 (0.513, 0.657) | 4 | 48.2 | 0.421 (0.317, 0.516) | 4 | 56.2 | 0.594 (0.566, 0.620) | 2 | N/A | 0.493 (0.322, 0.634) | 1 | 69.2 | N/A | N/A | N/A | N/A | N/A | N/A |
| SFA | 0.649 (0.616, 0.681) | 40 | 74.5 | 0.560 (0.503, 0.613) | 25 | 81.4 | 0.622 (0.583, 0.658) | 22 | 71.2 | 0.609 (0.524, 0.682) | 10 | 79.6 | 0.353 (0.183, 0.502) | 4 | 62.3 | 0.408 (0.266, 0.532) | 2 | 29.1 |
| Linoleic acid | 0.629 (0.540, 0.704) | 6 | 85.2 | 0.570 (0.435, 0.681) | 6 | 91 | 0.605 (0.533, 0.667) | 4 | 57.9 | 0.581 (0.469, 0.675) | 1 | NA | N/A | N/A | N/A | 0.597 (0.526, 0.659) | 2 | N/A |
| Linolenic acid | 0.697 (0.579, 0.786) | 3 | 85 | 0.704 (0.273, 0.900) | 2 | 96.8 | 0.592 (0.503, 0.668) | 1 | NA | N/A | N/A | N/A | N/A | N/A | N/A | 0.579 (0.462, 0.677) | 2 | 59.6 |
| EPA | 0.785 (0.579, 0.896) | 3 | 87 | N/A | N/A | N/A | N/A | N/A | N/A | N/A | N/A | N/A | N/A | N/A | N/A | N/A | N/A | N/A |
| DHA | 0.749 (0.616, 0.840) | 3 | 67.4 | N/A | N/A | N/A | N/A | N/A | N/A | N/A | N/A | N/A | N/A | N/A | N/A | N/A | N/A | N/A |
| Trans-fat | 0.705 (0.588, 0.792) | 4 | 72 | N/A | N/A | N/A | 0.339 (0.210, 0.455) | 1 | NA | N/A | N/A | N/A | 0.480 (0.259, 0.652) | 1 | NA | N/A | N/A | N/A |
| Cholesterol | 0.645 (0.606, 0.680) | 40 | 78.2 | 0.546 (0.476, 0.610) | 23 | 84.3 | 0.573 (0.529, 0.615) | 24 | 77.4 | 0.548 (0.462, 0.623) | 11 | 78.8 | 0.536 (0.119, 0.792) | 3 | 91.9 | 0.656 (0.588, 0.715) | 3 | 32.8 |
| Lipid | 0.516 (0.379, 0.632) | 2 | N/A | 0.626 (-0.06, 0.911) | 2 | 93.8 | 0.613 (0.499, 0.706) | 2 | N/A | 0.526 (0.329, 0.679) | 2 | 53.4 | 0.537 (0.460, 0.607) | 2 | N/A | N/A | N/A | N/A |
| Carbohydrate | 0.660 (0.625, 0.693) | 61 | 86.6 | 0.576 (0.526, 0.621) | 40 | 84.2 | 0.631 (0.588, 0.670) | 31 | 82.1 | 0.596 (0.525, 0.658) | 17 | 81.4 | 0.496 (0.337, 0.628) | 10 | 88.2 | 0.657 (0.571, 0.728) | 3 | 55.9 |
| Sucrose | 0.742 (0.696, 0.781) | 5 | 23.5 | N/A | N/A | N/A | 0.566 (0.219, 0.785) | 2 | 85.5 | N/A | N/A | N/A | N/A | N/A | N/A | N/A | N/A | N/A |
| Sugar | 0.711 (0.652, 0.762) | 5 | 60.8 | 0.721 (0.691, 0.750) | 2 | N/A | 0.724 (0.636, 0.794) | 4 | 69.1 | 0.669 (0.549, 0.762) | 2 | 27.2 | 0.506 (0.112, 0.762) | 2 | 86.8 | 0.296 (0.110, 0.462) | 1 | NA |
| Starch | 0.645 (0.599, 0.687) | 2 | N/A | N/A | N/A | N/A | 0.627 (0.563, 0.684) | 3 | N/A | N/A | N/A | N/A | N/A | N/A | N/A | N/A | N/A | N/A |
| Fiber | 0.652 (0.617, 0.684) | 54 | 79.3 | 0.626 (0.577, 0.670) | 35 | 82.8 | 0.665 (0.612, 0.711) | 24 | 87.5 | 0.611 (0.527, 0.683) | 14 | 83.7 | 0.469 (0.318, 0.597) | 10 | 85.7 | 0.615 (0.413, 0.759) | 4 | 91 |
| Soluble fiber | 0.644 (0.553, 0.720) | 10 | 76.5 | 0.591 (0.459, 0.698) | 8 | 81.6 | 0.698 (0.652, 0.738) | 4 | 65.8 | 0.599 (0.521, 0.669) | 2 | N/A | N/A | N/A | N/A | N/A | N/A | N/A |
| Insoluble fiber | 0.655 (0.584, 0.715) | 10 | 63 | 0.583 (0.457, 0.685) | 8 | 79.2 | 0.635 (0.561, 0.698) | 2 | N/A | 0.645 (0.596, 0.690) | 4 | N/A | N/A | N/A | N/A | N/A | N/A | N/A |
| Alcohol | 0.837 (0.796, 0.870) | 31 | 92.9 | 0.788 (0.726, 0.837) | 20 | 92.5 | 0.873 (0.827, 0.907) | 17 | 94 | 0.807 (0.745, 0.856) | 7 | 81.8 | N/A | N/A | N/A | N/A | N/A | N/A |
| Vitamin A | 0.639 (0.583, 0.689) | 30 | 89.2 | 0.599 (0.515, 0.672) | 15 | 86.9 | 0.574 (0.511, 0.630) | 8 | 76.8 | 0.512 (0.307, 0.672) | 5 | 91.7 | 0.489 (0.321, 0.627) | 4 | 76.1 | 0.258 (0.091, 0.410) | 2 | 38.2 |
| Retinol | 0.576 (0.534, 0.615) | 33 | 80.4 | 0.529 (0.471, 0.583) | 28 | 83.5 | 0.600 (0.532, 0.661) | 14 | 77.5 | 0.558 (0.463, 0.640) | 7 | 66 | 0.342 (0.053, 0.578) | 3 | 79.2 | 0.236 (0.148, 0.321) | 3 | N/A |
| Carotene | 0.654 (0.623, 0.684) | 53 | 80.4 | 0.613 (0.566, 0.656) | 34 | 82.2 | 0.614 (0.557, 0.665) | 30 | 88 | 0.573 (0.490, 0.646) | 19 | 83.4 | 0.426 (0.275, 0.558) | 10 | 83.7 | 0.538 (0.279, 0.724) | 4 | 93 |
| β-Carotene | 0.665 (0.632, 0.695) | 16 | 41.4 | 0.577 (0.510, 0.635) | 9 | 56.4 | 0.632 (0.534, 0.713) | 9 | 89.5 | 0.594 (0.344, 0.765) | 5 | 88.7 | 0.407 (0.200, 0.581) | 5 | 81.5 | 0.492 (0.338, 0.621) | 1 | NA |
| Vitamin E | 0.639 (0.577, 0.694) | 32 | 92.5 | 0.558 (0.472, 0.633) | 20 | 89.6 | 0.614 (0.550, 0.671) | 18 | 88.5 | 0.559 (0.454, 0.649) | 9 | 79.5 | 0.507 (0.238, 0.703) | 3 | 81.3 | 0.453 (0.292, 0.588) | 1 | NA |
| Vitamin K | 0.604 (0.505, 0.687) | 5 | 69.1 | 0.648 (0.560, 0.721) | 3 | N/A | 0.583 (0.156, 0.827) | 2 | 49.2 | 0.692 (0.104, 0.921) | 2 | 76.2 | N/A | N/A | N/A | N/A | N/A | N/A |
| Thiamin | 0.615 (0.585, 0.644) | 34 | 60 | 0.509 (0.447, 0.566) | 24 | 78.4 | 0.591 (0.537, 0.641) | 18 | 82.4 | 0.557 (0.473, 0.630) | 11 | 78.9 | 0.598 (0.364, 0.761) | 4 | 90 | 0.498 (0.283, 0.665) | 4 | 89.4 |
| Riboflavin | 0.652 (0.616, 0.684) | 34 | 77.7 | 0.590 (0.526, 0.649) | 24 | 86.2 | 0.640 (0.592, 0.684) | 17 | 80.6 | 0.589 (0.489, 0.674) | 8 | 81.5 | 0.511 (0.136, 0.758) | 4 | 93.8 | 0.469 (0.243, 0.647) | 3 | 87.4 |
| Niacin | 0.635 (0.575, 0.687) | 25 | 85.7 | 0.519 (0.435, 0.593) | 21 | 84.2 | 0.702 (0.492, 0.835) | 10 | 97.6 | 0.513 (0.368, 0.634) | 9 | 90.5 | 0.502 (0.228, 0.701) | 4 | 90.5 | 0.513 (0.331, 0.658) | 4 | 86.1 |
| Vitamin B6 | 0.633 (0.570, 0.689) | 20 | 77.2 | 0.557 (0.472, 0.631) | 15 | 77.7 | 0.633 (0.581, 0.681) | 5 | N/A | 0.551 (0.393, 0.676) | 4 | 80.5 | 0.283 (0.033, 0.499) | 2 | 68.2 | N/A | N/A | N/A |
| Folate | 0.646 (0.599, 0.687) | 32 | 82.8 | 0.627 (0.549, 0.694) | 18 | 84.8 | 0.587 (0.536, 0.634) | 14 | 69.8 | 0.596 (0.545, 0.643) | 7 | 15 | 0.385 (0.188, 0.551) | 3 | 70.7 | 0.268 (0.079, 0.438) | 1 | NA |
| Vitamin B12 | 0.679 (0.624, 0.727) | 18 | 75.9 | 0.583 (0.481, 0.670) | 14 | 87.9 | 0.560 (0.453, 0.652) | 8 | 71 | 0.537 (0.409, 0.644) | 7 | 76.1 | 0.385 (0.255, 0.501) | 2 | N/A | N/A | N/A | N/A |
| Carotene | 0.599 (0.539, 0.653) | 12 | 85.2 | 0.531 (0.376, 0.657) | 9 | 95.1 | 0.608 (0.530, 0.675) | 14 | 90.8 | 0.502 (0.401, 0.590) | 10 | 81.1 | N/A | N/A | N/A | 0.458 (0.373, 0.535) | 2 | N/A |
| β-Carotene | 0.621 (0.576, 0.663) | 29 | 73 | 0.562 (0.516, 0.603) | 20 | 50.9 | 0.616 (0.511, 0.703) | 7 | 73.2 | 0.545 (0.432, 0.641) | 7 | 71.2 | 0.510 (0.322, 0.660) | 3 | 73.9 | 0.433 (0.262, 0.577) | 1 | NA |
| Se | 0.664 (0.587, 0.729) | 12 | 85.8 | 0.606 (0.439, 0.731) | 8 | 90.3 | 0.590 (0.510, 0.660) | 2 | N/A | 0.465 (0.370, 0.550) | 2 | N/A | 0.571 (0.432, 0.684) | 1 | NA | 0.462 (0.303, 0.597) | 1 | NA |
| Mg | 0.711 (0.643, 0.768) | 21 | 90.2 | 0.671 (0.576, 0.748) | 12 | 84.8 | 0.597 (0.276, 0.798) | 5 | 89.6 | 0.538 (0.190, 0.766) | 4 | 88.6 | 0.476 (0.313, 0.612) | 4 | 70.3 | 0.563 (0.472, 0.642) | 3 | 45.2 |
| Ca | 0.656 (0.628, 0.682) | 49 | 72.2 | 0.598 (0.549, 0.644) | 33 | 82.9 | 0.608 (0.548, 0.661) | 27 | 87.6 | 0.567 (0.470, 0.650) | 18 | 87.6 | 0.463 (0.322, 0.584) | 12 | 86.6 | 0.581 (0.489, 0.659) | 4 | 57.5 |
| Fe | 0.627 (0.591, 0.661) | 42 | 79.5 | 0.568 (0.510, 0.621) | 28 | 83.4 | 0.671 (0.632, 0.706) | 22 | 71.9 | 0.610 (0.520, 0.686) | 15 | 83.3 | 0.426 (0.300, 0.536) | 12 | 81.8 | 0.447 (0.353, 0.533) | 4 | 45.6 |
| I | N/A | N/A | N/A | N/A | N/A | N/A | N/A | N/A | N/A | N/A | N/A | N/A | N/A | N/A | N/A | N/A | N/A | N/A |
| Zn | 0.634 (0.562, 0.697) | 20 | 87.9 | 0.599 (0.494, 0.687) | 13 | 84.2 | 0.613 (0.504, 0.703) | 3 | 63.2 | 0.502 (0.330, 0.641) | 1 | NA | 0.554 (0.428, 0.658) | 3 | 45.1 | 0.611 (0.367, 0.777) | 4 | 93.5 |
| Cu | 0.748 (0.620, 0.837) | 6 | 86.6 | 0.781 (0.674, 0.856) | 4 | 75.5 | N/A | N/A | N/A | N/A | N/A | N/A | N/A | N/A | N/A | 0.599 (0.480, 0.697) | 2 | 62.7 |
| K | 0.649 (0.605, 0.690) | 31 | 82.9 | 0.601 (0.542, 0.655) | 22 | 80.9 | 0.611 (0.562, 0.656) | 17 | 69.2 | 0.631 (0.580, 0.677) | 10 | 20.5 | 0.724 (0.591, 0.819) | 2 | 62.7 | 0.609 (0.513, 0.690) | 2 | 46.3 |
| P | 0.649 (0.606, 0.688) | 25 | 75.4 | 0.586 (0.513, 0.651) | 19 | 82.6 | 0.586 (0.474, 0.679) | 15 | 86.7 | 0.561 (0.434, 0.667) | 9 | 83.6 | 0.465 (-0.13, 0.814) | 3 | 95.8 | 0.575 (0.360, 0.732) | 2 | 86.2 |
| Na | 0.646 (0.603, 0.685) | 26 | 80.6 | 0.527 (0.445, 0.601) | 19 | 87.1 | 0.559 (0.469, 0.638) | 14 | 81 | 0.581 (0.422, 0.706) | 8 | 89.9 | 0.699 (0.469, 0.840) | 2 | 88.2 | 0.626 (0.553, 0.688) | 3 | 32.5 |
| Mn | 0.655 (0.596, 0.707) | 5 | N/A | N/A | N/A | N/A | N/A | N/A | N/A | N/A | N/A | N/A | N/A | N/A | N/A | N/A | N/A | N/A |

* CI, confidence interval; N/A: not available
