# Supplementary material for: A meta-analysis of the reproducibility of food frequency questionnaires in nutritional epidemiological studies
Source: Int J Behav Nutr Phys Act. 2021 Jan 11;18:12. doi: 10.1186/s12966-020-01078-4 (PMC7802360; doi:10.1186/s12966-020-01078-4)
Supplement: Supplementary file 5 — Additional file 5 Supplemental Table 4. Pooled intraclass correlation coefficients for energy and nutrients stratified by sex. [file 12966_2020_1078_MOESM5_ESM.docx]

**Supplemental Table 4**. Pooled intraclass correlation coefficients for energy and nutrients stratified by sex *

| Nutrient | Both | | | | | | Men | | | | | | Women | | | | | |
| --- | --- | --- | --- | --- | --- | --- | --- | --- | --- | --- | --- | --- | --- | --- | --- | --- | --- | --- |
|  | Crude | | | Energy-adjusted | | | Crude | | | Energy-adjusted | | | Crude | | | Energy-adjusted | | |
|  | SCC (95% CI) | N | *I^2^* | SCC (95% CI) | N | *I^2^* | SCC (95% CI) | N | *I^2^* | SCC (95% CI) | N | *I^2^* | SCC (95% CI) | N | *I^2^* | SCC (95% CI) | N | *I^2^* |
| Energy | 0.718 (0.625, 0.792) | 38 | 97.3 | N/A | N/A | N/A | 0.651 (0.517, 0.754) | 10 | 93 | N/A | N/A | N/A | 0.719 (0.656, 0.771) | 15 | 87.5 | N/A | N/A | N/A |
| Protein | 0.639 (0.588, 0.686) | 40 | 89.5 | 0.619 (0.536, 0.690) | 14 | 85.3 | 0.639 (0.588, 0.686) | 10 | 85.8 | 0.577 (0.443, 0.684) | 5 | 64.7 | 0.683 (0.621, 0.736) | 15 | 84.4 | 0.573 (0.482, 0.651) | 6 | 67.7 |
| Fat | 0.625 (0.574, 0.672) | 39 | 87 | 0.583 (0.477, 0.672) | 14 | 86.2 | 0.645 (0.489, 0.762) | 7 | 93.9 | 0.458 (0.313, 0.583) | 2 | 40.1 | 0.699 (0.627, 0.759) | 11 | 88.7 | 0.549 (0.346, 0.703) | 3 | 92.7 |
| Plant fat | 0.616 (0.494, 0.714) | 3 | 60.4 | N/A | N/A | N/A | 0.549 (0.346, 0.704) | 1 | N/A | N/A | N/A | N/A | 0.410 (0.178, 0.598) | 1 | N/A | N/A | N/A | N/A |
| Animal fat | N/A | N/A | N/A | N/A | N/A | N/A | N/A | N/A | N/A | N/A | N/A | N/A | N/A | N/A | N/A | N/A | N/A | N/A |
| MUFA | 0.639 (0.586, 0.687) | 31 | 82.9 | 0.653 (0.558, 0.732) | 12 | 85.3 | 0.597 (0.409, 0.736) | 4 | 80.8 | 0.450 (0.264, 0.603) | 2 | N/A | 0.671 (0.632, 0.707) | 8 | 44.4 | 0.614 (0.489, 0.714) | 4 | 76 |
| PUFA | 0.642 (0.537, 0.727) | 34 | 95.8 | 0.605 (0.491, 0.697) | 12 | 87.2 | 0.606 (0.397, 0.756) | 4 | 84.7 | 0.310 (0.106, 0.488) | 2 | N/A | 0.648 (0.594, 0.696) | 9 | 69.8 | 0.569 (0.431, 0.681) | 4 | 77.2 |
| n-3 PUFA | N/A | N/A | N/A | N/A | N/A | N/A | N/A | N/A | N/A | N/A | N/A | N/A | N/A | N/A | N/A | N/A | N/A | N/A |
| n-6 PUFA | N/A | N/A | N/A | N/A | N/A | N/A | N/A | N/A | N/A | N/A | N/A | N/A | N/A | N/A | N/A | N/A | N/A | N/A |
| SFA | 0.678 (0.557, 0.770) | 36 | 97.3 | 0.671 (0.573, 0.750) | 13 | 88 | 0.749 (0.689, 0.799) | 5 | 33.6 | 0.599 (0.445, 0.719) | 2 | N/A | 0.708 (0.632, 0.771) | 10 | 89 | 0.542 (0.359, 0.684) | 4 | 85.8 |
| Linoleic acid | 0.671 (0.369, 0.845) | 4 | 94.9 | 0.730 (0.663, 0.785) | 2 | 0.4 | N/A | N/A | N/A | N/A | N/A | N/A | 0.660 (0.613, 0.701) | 1 | N/A | 0.629 (0.580, 0.674) | 1 | N/A |
| Linolenic acid | 0.651 (0.375, 0.821) | 3 | 89.3 | N/A | N/A | N/A | N/A | N/A | N/A | N/A | N/A | N/A | 0.690 (0.646, 0.728) | 1 | N/A | N/A | N/A | N/A |
| EPA | N/A | N/A | N/A | N/A | N/A | N/A | N/A | N/A | N/A | N/A | N/A | N/A | N/A | N/A | N/A | N/A | N/A | N/A |
| DHA | N/A | N/A | N/A | N/A | N/A | N/A | N/A | N/A | N/A | N/A | N/A | N/A | N/A | N/A | N/A | N/A | N/A | N/A |
| Trans-fat | 0.628 (0.199, 0.855) | 2 | 88.4 | N/A | N/A | N/A | N/A | N/A | N/A | N/A | N/A | N/A | 0.590 (0.401, 0.731) | 2 | 82.7 | N/A | N/A | N/A |
| Cholesterol | 0.650 (0.581, 0.711) | 33 | 91.4 | 0.644 (0.560, 0.715) | 14 | 84.1 | 0.775 (0.735, 0.808) | 5 | 16.3 | 0.644 (0.560, 0.715) | 6 | 44.3 | 0.666 (0.582, 0.736) | 12 | 90 | 0.496 (0.329, 0.632) | 5 | 83.4 |
| Lipid | 0.800 (0.605, 0.904) | 2 | 92 | 0.720 (0.211, 0.921) | 2 | 97.3 | 0.649 (0.476, 0.775) | 1 | N/A | 0.610 (0.423, 0.746) | 1 | N/A | 0.450 (0.226, 0.629) | 1 | N/A | 0.570 (0.373, 0.717) | 1 | N/A |
| Carbohydrate | 0.671 (0.567, 0.754) | 41 | 97.6 | 0.671 (0.567, 0.754) | 41 | 97.6 | 0.632 (0.511, 0.729) | 8 | 90.2 | 0.632 (0.511, 0.729) | 8 | 90.2 | 0.720 (0.665, 0.768) | 15 | 83 | 0.720 (0.665, 0.768) | 15 | 83 |
| Sucrose | 0.603 (0.487, 0.697) | 3 | 67.9 | N/A | N/A | N/A | N/A | N/A | N/A | N/A | N/A | N/A | 0.709 (0.627, 0.776) | 1 | N/A | N/A | N/A | N/A |
| Sugar | 0.682 (0.537, 0.787) | 5 | 83.6 | N/A | N/A | N/A | 0.660 (0.393, 0.824) | 1 | N/A | N/A | N/A | N/A | 0.771 (0.651, 0.852) | 2 | 67.9 | N/A | N/A | N/A |
| Starch | 0.510 (0.264, 0.693) | 3 | 85.5 | N/A | N/A | N/A | N/A | N/A | N/A | N/A | N/A | N/A | N/A | N/A | N/A | N/A | N/A | N/A |
| Fiber | 0.674 (0.613, 0.727) | 35 | 90.8 | 0.705 (0.614, 0.777) | 14 | 91.4 | 0.723 (0.564, 0.831) | 5 | 91.9 | 0.654 (0.527, 0.752) | 2 | N/A | 0.692 (0.615, 0.755) | 14 | 91.7 | 0.560 (0.363, 0.709) | 5 | 92.4 |
| Soluble fiber | N/A | N/A | N/A | N/A | N/A | N/A | N/A | N/A | N/A | N/A | N/A | N/A | N/A | N/A | N/A | N/A | N/A | N/A |
| Insoluble fiber | N/A | N/A | N/A | N/A | N/A | N/A | N/A | N/A | N/A | N/A | N/A | N/A | N/A | N/A | N/A | N/A | N/A | N/A |
| Alcohol | 0.807 (0.752, 0.851) | 14 | 84.5 | 0.776 (0.688, 0.841) | 3 | 57.6 | 0.731 (0.632, 0.806) | 3 | N/A | 0.782 (0.654, 0.867) | 3 | 73.8 | 0.819 (0.648, 0.911) | 5 | 95 | 0.848 (0.709, 0.923) | 3 | 85.8 |
| Vitamin A | 0.636 (0.536, 0.719) | 18 | 94.6 | 0.611 (0.459, 0.728) | 10 | 93.5 | 0.655 (0.233, 0.869) | 3 | 95.7 | 0.459 (0.235, 0.638) | 1 | N/A | 0.679 (0.551, 0.776) | 8 | 92.3 | 0.579 (0.385, 0.724) | 1 | N/A |
| Retinol | 0.583 (0.484, 0.666) | 12 | 88.1 | 0.596 (0.464, 0.702) | 4 | 68.2 | 0.699 (0.573, 0.793) | 2 | N/A | 0.679 (0.547, 0.779) | 2 | N/A | 0.576 (0.462, 0.671) | 4 | 52 | 0.327 (0.211, 0.434) | 3 | N/A |
| Carotene | 0.561 (0.330, 0.728) | 4 | 95.9 | 0.456 (0.172, 0.671) | 3 | 91.9 | 0.667 (0.298, 0.863) | 2 | 92.9 | 0.619 (0.436, 0.753) | 1 | N/A | 0.699 (0.583, 0.788) | 3 | 81.7 | 0.579 (0.385, 0.724) | 1 | N/A |
| β-Carotene | 0.697 (0.624, 0.757) | 11 | 77.3 | 0.750 (0.628, 0.835) | 1 | N/A | 0.735 (0.681, 0.781) | 4 | 3.5 | 0.750 (0.640, 0.829) | 2 | N/A | 0.666 (0.554, 0.755) | 6 | 85.3 | 0.421 (0.012, 0.708) | 3 | 90.2 |
| Vitamin C | 0.685 (0.593, 0.759) | 32 | 96.8 | 0.698 (0.566, 0.795) | 14 | 95.5 | 0.588 (0.294, 0.780) | 5 | 94.2 | 0.344 (0.196, 0.476) | 3 | 12.9 | 0.669 (0.583, 0.740) | 12 | 90.8 | 0.552 (0.349, 0.705) | 5 | 90.7 |
| Vitamin D | 0.677 (0.400, 0.841) | 10 | 98.9 | 0.743 (0.319, 0.919) | 3 | 98.2 | 0.800 (0.774, 0.822) | 1 | N/A | N/A | N/A | N/A | 0.649 (0.527, 0.746) | 5 | 93.4 | 0.533 (0.164, 0.771) | 2 | 96.6 |
| Vitamin E | 0.657 (0.515, 0.764) | 24 | 98.1 | 0.622 (0.455, 0.746) | 11 | 95.4 | 0.675 (0.546, 0.773) | 3 | 70 | 0.649 (0.476, 0.775) | 1 | N/A | 0.703 (0.627, 0.766) | 9 | 88.2 | 0.525 (0.284, 0.703) | 3 | 91.3 |
| Vitamin K | 0.636 (0.329, 0.821) | 3 | 93 | N/A | N/A | N/A | N/A | N/A | N/A | N/A | N/A | N/A | 0.709 (0.669, 0.746) | 1 | N/A | N/A | N/A | N/A |
| Thiamin | 0.628 (0.567, 0.683) | 23 | 88.6 | 0.613 (0.469, 0.726) | 10 | 93.6 | 0.670 (0.630, 0.705) | 1 | N/A | N/A | N/A | N/A | 0.633 (0.570, 0.688) | 7 | 73.4 | 0.571 (0.281, 0.766) | 2 | 94.5 |
| Riboflavin | 0.644 (0.575, 0.704) | 19 | 90.5 | 0.641 (0.442, 0.779) | 7 | 95.8 | 0.800 (0.774, 0.822) | 1 | N/A | N/A | N/A | N/A | 0.700 (0.639, 0.752) | 8 | 79.7 | 0.566 (0.315, 0.742) | 3 | 92.4 |
| Niacin | 0.670 (0.589, 0.737) | 16 | 91.8 | 0.586 (0.443, 0.700) | 7 | 89.1 | N/A | N/A | N/A | 0.800 (0.743, 0.845) | 1 | N/A | 0.648 (0.614, 0.681) | 6 | N/A | 0.542 (0.345, 0.693) | 2 | 88.2 |
| Vitamin B6 | 0.744 (0.496, 0.880) | 11 | 98.5 | 0.733 (0.499, 0.867) | 4 | 95.7 | N/A | N/A | N/A | N/A | N/A | N/A | 0.586 (0.471, 0.681) | 2 | 73 | 0.619 (0.569, 0.665) | 1 | N/A |
| Folate | 0.622 (0.555, 0.681) | 21 | 87.5 | 0.598 (0.443, 0.719) | 5 | 81.1 | 0.779 (0.752, 0.805) | 1 | N/A | N/A | N/A | N/A | 0.665 (0.499, 0.783) | 3 | 95.3 | 0.599 (0.547, 0.647) | 1 | N/A |
| Vitamin B12 | 0.699 (0.489, 0.833) | 11 | 97.8 | 0.734 (0.524, 0.860) | 5 | 95.6 | N/A | N/A | N/A | N/A | N/A | N/A | 0.546 (0.434, 0.641) | 2 | 71.5 | 0.532 (0.389, 0.650) | 2 | 81.1 |
| Se | 0.673 (0.621, 0.719) | 8 | 58.4 | 0.586 (0.429, 0.709) | 4 | 78.7 | N/A | 1 | N/A | N/A | N/A | N/A | 0.642 (0.439, 0.782) | 3 | 82.9 | N/A | N/A | N/A |
| Mg | 0.684 (0.603, 0.752) | 12 | 88.6 | 0.656 (0.567, 0.729) | 4 | 49.1 | N/A | N/A | N/A | N/A | N/A | N/A | 0.660 (0.535, 0.757) | 7 | 88.8 | 0.536 (0.141, 0.784) | 2 | 97 |
| Ca | 0.630 (0.582, 0.675) | 35 | 87.2 | 0.678 (0.586, 0.753) | 14 | 91.1 | 0.709 (0.581, 0.804) | 6 | 90.4 | 0.691 (0.595, 0.766) | 4 | 39.5 | 0.602 (0.481, 0.700) | 13 | 94.6 | 0.502 (0.333, 0.641) | 7 | 91.1 |
| Fe | 0.619 (0.560, 0.673) | 26 | 87.9 | 0.580 (0.476, 0.667) | 12 | 85.9 | 0.684 (0.473, 0.821) | 4 | 93.9 | 0.551 (0.207, 0.773) | 2 | 72.7 | 0.675 (0.583, 0.749) | 9 | 90.7 | 0.533 (0.463, 0.596) | 5 | 27.4 |
| I | N/A | N/A | N/A | N/A | N/A | N/A | N/A | N/A | N/A | N/A | N/A | N/A | N/A | N/A | N/A | N/A | N/A | N/A |
| Zn | 0.590 (0.527, 0.648) | 14 | 79.5 | 0.591 (0.462, 0.696) | 6 | 78.6 | 0.489 (0.397, 0.570) | 3 | N/A | 0.679 (0.547, 0.779) | 2 | N/A | 0.613 (0.580, 0.644) | 9 | N/A | 0.497 (0.341, 0.626) | 4 | 76.8 |
| Cu | N/A | N/A | N/A | N/A | N/A | N/A | N/A | N/A | N/A | N/A | N/A | N/A | 0.658 (0.620, 0.693) | 4 | N/A | N/A | N/A | N/A |
| K | 0.626 (0.542, 0.697) | 17 | 90.3 | 0.630 (0.428, 0.772) | 6 | 93.5 | 0.839 (0.819, 0.858) | 1 | N/A | 0.679 (0.635, 0.719) | 1 | N/A | 0.736 (0.679, 0.784) | 7 | 77.4 | N/A | N/A | N/A |
| P | 0.559 (0.430, 0.665) | 13 | 93.3 | 0.646 (0.529, 0.740) | 3 | 67.1 | 0.660 (0.521, 0.764) | 2 | N/A | 0.709 (0.586, 0.800) | 2 | N/A | 0.658 (0.568, 0.733) | 8 | 75.2 | 0.594 (0.378, 0.749) | 4 | 91 |
| Na | 0.661 (0.449, 0.802) | 19 | 98.7 | 0.668 (0.393, 0.833) | 7 | 97.4 | N/A | N/A | N/A | N/A | N/A | N/A | 0.653 (0.578, 0.718) | 6 | 58.5 | 0.679 (0.635, 0.719) | 1 | N/A |
| Mn | N/A | N/A | N/A | N/A | N/A | N/A | N/A | N/A | N/A | N/A | N/A | N/A | N/A | N/A | N/A | N/A | N/A | N/A |

* CI, confidence interval; N/A: not available
