# Supplementary material for: A meta-analysis of the reproducibility of food frequency questionnaires in nutritional epidemiological studies
Source: Int J Behav Nutr Phys Act. 2021 Jan 11;18:12. doi: 10.1186/s12966-020-01078-4 (PMC7802360; doi:10.1186/s12966-020-01078-4)
Supplement: Supplementary file 6 — Additional file 6 Supplemental Table 5. Pooled spearman correlation coefficients for energy and nutrients stratified by sex. [file 12966_2020_1078_MOESM6_ESM.docx]

**Supplemental Table 5.** Pooled spearman correlation coefficients for energy and nutrients stratified by sex

| Nutrient | Both | | | | | | Men | | | | | | Women | | | | | |
| --- | --- | --- | --- | --- | --- | --- | --- | --- | --- | --- | --- | --- | --- | --- | --- | --- | --- | --- |
|  | Crude | | | Energy-adjusted | | | Crude | | | Energy-adjusted | | | Crude | | | Energy-adjusted | | |
|  | SCC (95% CI) | N | *I^2^* | SCC (95% CI) | N | *I^2^* | SCC (95% CI) | N | *I^2^* | SCC (95% CI) | N | *I^2^* | SCC (95% CI) | N | *I^2^* | SCC (95% CI) | N | *I^2^* |
| Energy | 0.644 (0.605, 0.679) | 60 | 87.7 | N/A | N/A | N/A | 0.646 (0.592, 0.693) | 21 | 76.6 | N/A | N/A | N/A | 0.616 (0.579, 0.650) | 39 | 74.4 | N/A | N/A | N/A |
| Protein | 0.602 (0.562, 0.639) | 59 | 86.8 | 0.565 (0.511, 0.615) | 30 | 84.1 | 0.610 (0.555, 0.659) | 21 | 74.7 | 0.560 (0.463, 0.644) | 13 | 80.3 | 0.587 (0.555, 0.618) | 40 | 63.2 | 0.527 (0.475, 0.576) | 25 | 69.7 |
| Fat | 0.622 (0.587, 0.654) | 60 | 84.2 | 0.554 (0.498, 0.605) | 27 | 82.9 | 0.614 (0.558, 0.664) | 20 | 74.9 | 0.494 (0.406, 0.573) | 11 | 68.5 | 0.604 (0.575, 0.631) | 38 | 53.8 | 0.571 (0.505, 0.630) | 22 | 81.5 |
| Plant fat | 0.548 (0.468, 0.619) | 6 | 62 | N/A | N/A | N/A | 0.374 (0.274, 0.466) | 1 | N/A | N/A | N/A | N/A | 0.502 (0.370, 0.614) | 1 | N/A | N/A | N/A | N/A |
| Animal fat | 0.693 (0.661, 0.722) | 4 | 0 | N/A | N/A | N/A | 0.652 (0.583, 0.711) | 1 | N/A | N/A | N/A | N/A | 0.683 (0.585, 0.760) | 1 | N/A | N/A | N/A | N/A |
| MUFA | 0.636 (0.590, 0.677) | 38 | 97.3 | 0.597 (0.519, 0.665) | 17 | 83.4 | 0.546 (0.474, 0.611) | 10 | 93 | 0.484 (0.352, 0.597) | 6 | 75.2 | 0.596 (0.559, 0.631) | 15 | 87.5 | 0.506 (0.417, 0.585) | 9 | 70.5 |
| PUFA | 0.606 (0.558, 0.649) | 33 | 80.6 | 0.553 (0.483, 0.616) | 19 | 81.5 | 0.571 (0.540, 0.601) | 10 | 0.7 | 0.496 (0.329, 0.633) | 5 | 79.7 | 0.586 (0.549, 0.621) | 16 | 44.2 | 0.452 (0.382, 0.517) | 7 | 34.8 |
| n-3 PUFA | 0.699 (0.544, 0.809) | 1 | N/A | 0.450 (0.223, 0.630) | 1 | N/A | 0.577 (0.521, 0.629) | 5 | 29.2 | 0.474 (0.364, 0.572) | 4 | 46.2 | 0.653 (0.568, 0.723) | 5 | 74.8 | 0.472 (0.352, 0.577) | 4 | 56.9 |
| n-6 PUFA | 0.570 (0.371, 0.718) | 1 | N/A | 0.499 (0.283, 0.667) | 1 | N/A | 0.589 (0.546, 0.629) | 5 | 8.3 | 0.434 (0.222, 0.607) | 4 | 83.1 | 0.604 (0.541, 0.660) | 5 | 48 | 0.434 (0.355, 0.507) | 4 | 0 |
| SFA | 0.629 (0.585, 0.669) | 35 | 80 | 0.603 (0.543, 0.657) | 19 | 78.8 | 0.628 (0.562, 0.686) | 11 | 77 | 0.550 (0.413, 0.662) | 6 | 80.1 | 0.616 (0.579, 0.651) | 21 | 65 | 0.516 (0.432, 0.591) | 12 | 80.9 |
| Linoleic acid | 0.682 (0.626, 0.731) | 4 | 59.6 | 0.643 (0.550, 0.720) | 5 | 81.1 | 0.567 (0.506, 0.623) | 2 | 0 | N/A | N/A | N/A | 0.564 (0.492, 0.627) | 5 | 62.1 | 0.469 (0.360, 0.564) | 4 | 69 |
| Linolenic acid | 0.684 (0.576, 0.769) | 3 | 86.3 | 0.642 (0.486, 0.759) | 1 | N/A | 0.571 (0.492, 0.642) | 1 | N/A | N/A | N/A | N/A | 0.611 (0.499, 0.703) | 1 | N/A | N/A | N/A | N/A |
| EPA | 0.785 (0.579, 0.896) | 3 | 87 | N/A | N/A | N/A | N/A | N/A | N/A | N/A | N/A | N/A | N/A | N/A | N/A | N/A | N/A | N/A |
| DHA | 0.749 (0.616, 0.840) | 3 | 67.4 | N/A | N/A | N/A | N/A | N/A | N/A | N/A | N/A | N/A | N/A | N/A | N/A | N/A | N/A | N/A |
| Trans-fat | 0.646 (0.451, 0.782) | 4 | 83.9 | N/A | N/A | N/A | N/A | N/A | N/A | N/A | N/A | N/A | 0.557 (0.077, 0.827) | 2 | 95.1 | N/A | N/A | N/A |
| Cholesterol | 0.630 (0.582, 0.673) | 33 | 81.6 | 0.620 (0.549, 0.683) | 17 | 84.2 | 0.605 (0.527, 0.672) | 12 | 84.8 | 0.545 (0.367, 0.684) | 6 | 87.6 | 0.599 (0.560, 0.635) | 23 | 67.2 | 0.480 (0.424, 0.532) | 14 | 56.6 |
| Lipid | 0.532 (0.466, 0.592) | 4 | 0 | 0.626 (-0.06, 0.911) | 2 | 93.8 | 0.592 (0.412, 0.726) | 1 | N/A | 0.611 (0.439, 0.741) | 1 | N/A | 0.632 (0.475, 0.750) | 1 | N/A | 0.433 (0.231, 0.599) | 1 | N/A |
| Carbohydrate | 0.613 (0.568, 0.655) | 57 | 89.9 | 0.586 (0.529, 0.636) | 30 | 86.1 | 0.640 (0.581, 0.693) | 21 | 80.9 | 0.570 (0.487, 0.644) | 13 | 74.5 | 0.613 (0.573, 0.650) | 37 | 78 | 0.566 (0.499, 0.627) | 21 | 82.3 |
| Sucrose | 0.759 (0.658, 0.834) | 1 | N/A | N/A | N/A | N/A | 0.748 (0.594, 0.849) | 2 | 83.1 | N/A | N/A | N/A | 0.670 (0.557, 0.759) | 4 | 71.2 | N/A | N/A | N/A |
| Sugar | 0.685 (0.613, 0.746) | 9 | 82.1 | 0.622 (0.435, 0.757) | 3 | 94.1 | 0.621 (0.439, 0.755) | 1 | N/A | 0.601 (0.412, 0.741) | 1 | N/A | 0.765 (0.664, 0.838) | 1 | N/A | 0.713 (0.595, 0.801) | 1 | N/A |
| Starch | 0.642 (0.602, 0.678) | 2 | 0 | N/A | N/A | N/A | 0.672 (0.612, 0.724) | 2 | 0 | N/A | N/A | N/A | 0.587 (0.496, 0.665) | 2 | 0 | N/A | N/A | N/A |
| Fiber | 0.624 (0.575, 0.669) | 49 | 88.6 | 0.631 (0.571, 0.684) | 26 | 86.7 | 0.667 (0.617, 0.711) | 14 | 63.2 | 0.683 (0.621, 0.737) | 8 | 50 | 0.630 (0.588, 0.669) | 30 | 74.4 | 0.573 (0.510, 0.628) | 19 | 72.7 |
| Soluble fiber | 0.710 (0.492, 0.845) | 4 | 87.4 | 0.775 (0.439, 0.921) | 2 | 85.3 | 0.644 (0.524, 0.738) | 5 | 85 | 0.539 (0.332, 0.695) | 4 | 85.3 | 0.649 (0.583, 0.706) | 5 | 59.4 | 0.551 (0.483, 0.613) | 4 | 0 |
| Insoluble fiber | 0.693 (0.486, 0.826) | 4 | 85.3 | 0.727 (0.510, 0.857) | 2 | 64.4 | 0.636 (0.574, 0.690) | 4 | 0 | 0.594 (0.430, 0.720) | 5 | 85.9 | 0.632 (0.573, 0.685) | 4 | 0 | 0.573 (0.496, 0.641) | 5 | 43.1 |
| Alcohol | 0.841 (0.797, 0.877) | 22 | 93.2 | 0.777 (0.699, 0.836) | 12 | 92.5 | 0.872 (0.801, 0.919) | 12 | 95.3 | 0.823 (0.748, 0.877) | 7 | 84.8 | 0.838 (0.782, 0.881) | 15 | 91.2 | 0.789 (0.675, 0.866) | 8 | 92.5 |
| Vitamin A | 0.621 (0.563, 0.673) | 31 | 89.8 | 0.565 (0.465, 0.651) | 17 | 91.8 | 0.569 (0.532, 0.605) | 5 | 0 | 0.542 (0.468, 0.610) | 3 | 0 | 0.574 (0.516, 0.627) | 12 | 66.3 | 0.521 (0.446, 0.590) | 6 | 37.5 |
| Retinol | 0.562 (0.507, 0.612) | 25 | 86.2 | 0.499 (0.416, 0.575) | 17 | 88.1 | 0.581 (0.486, 0.661) | 11 | 80.7 | 0.515 (0.378, 0.630) | 9 | 83.7 | 0.575 (0.542, 0.605) | 19 | 26.2 | 0.529 (0.466, 0.586) | 16 | 71.6 |
| Vitamin C | 0.598 (0.553, 0.640) | 51 | 88.6 | 0.589 (0.524, 0.647) | 27 | 88.7 | 0.642 (0.585, 0.693) | 18 | 77.4 | 0.629 (0.571, 0.680) | 12 | 53 | 0.611 (0.565, 0.653) | 33 | 80.3 | 0.553 (0.487, 0.611) | 22 | 77.8 |
| Vitamin D | 0.605 (0.537, 0.665) | 16 | 84.3 | 0.568 (0.494, 0.635) | 7 | 59.6 | 0.538 (0.378, 0.667) | 5 | 78.5 | 0.430 (0.220, 0.601) | 1 | N/A | 0.607 (0.510, 0.690) | 13 | 85.5 | 0.594 (0.411, 0.730) | 7 | 87.7 |
| Vitamin E | 0.634 (0.552, 0.703) | 25 | 94.9 | 0.588 (0.483, 0.676) | 14 | 92 | 0.649 (0.572, 0.714) | 10 | 81.5 | 0.604 (0.498, 0.692) | 4 | 34.1 | 0.577 (0.526, 0.625) | 21 | 75.6 | 0.488 (0.416, 0.555) | 12 | 63.2 |
| Vitamin K | 0.602 (0.436, 0.728) | 3 | 82 | 0.699 (0.544, 0.809) | 1 | N/A | 0.649 (0.492, 0.766) | 1 | N/A | 0.670 (0.519, 0.780) | 1 | N/A | 0.560 (0.411, 0.680) | 3 | 1.5 | 0.645 (0.373, 0.815) | 3 | 60.3 |
| Thiamin | 0.618 (0.579, 0.653) | 34 | 80.3 | 0.543 (0.478, 0.602) | 21 | 83.8 | 0.616 (0.565, 0.664) | 9 | 55.6 | 0.472 (0.359, 0.572) | 8 | 75.1 | 0.577 (0.542, 0.610) | 18 | 32.8 | 0.489 (0.423, 0.551) | 14 | 61.3 |
| Riboflavin | 0.632 (0.588, 0.672) | 32 | 86 | 0.612 (0.544, 0.672) | 20 | 88.9 | 0.663 (0.605, 0.714) | 10 | 72.1 | 0.492 (0.361, 0.604) | 7 | 77.9 | 0.631 (0.596, 0.663) | 18 | 43.9 | 0.549 (0.502, 0.592) | 12 | 25.1 |
| Niacin | 0.641 (0.571, 0.701) | 23 | 90.9 | 0.556 (0.470, 0.632) | 19 | 88.9 | 0.708 (0.421, 0.867) | 8 | 97.7 | 0.466 (0.308, 0.599) | 9 | 88.4 | 0.583 (0.518, 0.642) | 12 | 64.4 | 0.380 (0.307, 0.449) | 10 | 41.3 |
| Vitamin B6 | 0.601 (0.489, 0.694) | 13 | 86.2 | 0.611 (0.473, 0.720) | 8 | 86.4 | 0.516 (0.304, 0.679) | 5 | 83.9 | 0.506 (0.419, 0.583) | 3 | 0 | 0.584 (0.507, 0.653) | 13 | 71.8 | 0.516 (0.423, 0.598) | 8 | 62.4 |
| Folate | 0.577 (0.515, 0.634) | 29 | 87.2 | 0.605 (0.482, 0.703) | 12 | 90.2 | 0.615 (0.542, 0.679) | 10 | 69.5 | 0.598 (0.515, 0.671) | 5 | 48.7 | 0.615 (0.553, 0.669) | 16 | 73.3 | 0.614 (0.539, 0.680) | 9 | 63.3 |
| Vitamin B12 | 0.658 (0.573, 0.729) | 14 | 85.1 | 0.616 (0.497, 0.712) | 9 | 88 | 0.492 (0.328, 0.628) | 5 | 73 | 0.467 (0.301, 0.606) | 3 | 66.6 | 0.605 (0.518, 0.679) | 13 | 76.1 | 0.559 (0.431, 0.665) | 9 | 82.3 |
| Carotene | 0.648 (0.573, 0.712) | 13 | 92.8 | 0.541 (0.398, 0.657) | 10 | 94.5 | 0.612 (0.568, 0.653) | 5 | 24.1 | 0.522 (0.443, 0.593) | 4 | 17.3 | 0.543 (0.470, 0.608) | 9 | 79.1 | 0.451 (0.368, 0.525) | 7 | 62.9 |
| β-Carotene | 0.573 (0.522, 0.621) | 14 | 54.4 | 0.550 (0.505, 0.593) | 8 | 8 | 0.639 (0.563, 0.705) | 12 | 73.9 | 0.560 (0.477, 0.632) | 10 | 65 | 0.610 (0.544, 0.668) | 17 | 76.4 | 0.533 (0.473, 0.588) | 14 | 57.3 |
| Se | 0.690 (0.583, 0.773) | 8 | 90 | 0.673 (0.439, 0.821) | 5 | 92.7 | 0.537 (0.457, 0.608) | 3 | 0 | 0.452 (0.334, 0.556) | 3 | 41 | 0.641 (0.583, 0.692) | 4 | 0 | 0.485 (0.402, 0.560) | 3 | 0 |
| Mg | 0.678 (0.596, 0.746) | 18 | 90.8 | 0.650 (0.566, 0.721) | 12 | 81.8 | 0.653 (0.388, 0.819) | 4 | 86.6 | 0.750 (0.627, 0.836) | 1 | N/A | 0.584 (0.444, 0.696) | 12 | 87.1 | 0.551 (0.328, 0.716) | 6 | 89.3 |
| Ca | 0.619 (0.581, 0.654) | 53 | 84.7 | 0.630 (0.575, 0.681) | 28 | 86.4 | 0.601 (0.527, 0.667) | 15 | 81.5 | 0.568 (0.485, 0.642) | 9 | 65.2 | 0.606 (0.558, 0.651) | 29 | 78.6 | 0.520 (0.458, 0.577) | 20 | 72.7 |
| Fe | 0.594 (0.551, 0.634) | 49 | 86.8 | 0.562 (0.502, 0.618) | 26 | 85.4 | 0.601 (0.534, 0.660) | 15 | 77.3 | 0.567 (0.462, 0.656) | 10 | 80.2 | 0.595 (0.537, 0.647) | 23 | 78.1 | 0.562 (0.482, 0.634) | 15 | 76.9 |
| I | N/A | N/A | N/A | N/A | N/A | N/A | N/A | N/A | N/A | N/A | N/A | N/A | N/A | N/A | N/A | N/A | N/A | N/A |
| Zn | 0.606 (0.535, 0.668) | 18 | 86.2 | 0.594 (0.473, 0.694) | 13 | 89.8 | 0.774 (0.630, 0.866) | 2 | 64.3 | 0.649 (0.492, 0.766) | 1 | N/A | 0.617 (0.501, 0.711) | 6 | 73.8 | 0.596 (0.464, 0.702) | 4 | 66.8 |
| Cu | 0.789 (0.563, 0.905) | 3 | 91.4 | 0.742 (0.564, 0.854) | 4 | 92 | 0.649 (0.492, 0.766) | 1 | N/A | 0.690 (0.545, 0.794) | 1 | N/A | 0.723 (0.426, 0.880) | 2 | 89.3 | 0.699 (0.565, 0.798) | 1 | N/A |
| K | 0.627 (0.574, 0.675) | 26 | 86.1 | 0.639 (0.591, 0.683) | 16 | 69.7 | 0.661 (0.618, 0.700) | 13 | 53.8 | 0.551 (0.447, 0.641) | 9 | 76.7 | 0.632 (0.590, 0.671) | 18 | 53.3 | 0.566 (0.491, 0.632) | 13 | 67 |
| P | 0.596 (0.532, 0.654) | 24 | 86.8 | 0.624 (0.562, 0.680) | 15 | 79.4 | 0.686 (0.623, 0.741) | 8 | 58 | 0.537 (0.404, 0.649) | 7 | 80.9 | 0.610 (0.527, 0.680) | 15 | 77.5 | 0.507 (0.417, 0.587) | 12 | 71.6 |
| N/A | 0.612 (0.555, 0.663) | 27 | 88.8 | 0.542 (0.446, 0.626) | 17 | 91.5 | 0.618 (0.580, 0.654) | 9 | 0 | 0.486 (0.385, 0.577) | 8 | 66.3 | 0.627 (0.568, 0.679) | 11 | 55.7 | 0.577 (0.519, 0.630) | 9 | 39.4 |
| Mn | 0.643 (0.533, 0.731) | 2 | 6.9 | N/A | N/A | N/A | 0.720 (0.586, 0.815) | 1 | N/A | N/A | N/A | N/A | 0.639 (0.545, 0.718) | 2 | 2.6 | N/A | N/A | N/A |
