# Supplementary material for: A meta-analysis of the reproducibility of food frequency questionnaires in nutritional epidemiological studies
Source: Int J Behav Nutr Phys Act. 2021 Jan 11;18:12. doi: 10.1186/s12966-020-01078-4 (PMC7802360; doi:10.1186/s12966-020-01078-4)
Supplement: Supplementary file 7 — Additional file 7 Supplemental Table 6. Pooled crude intraclass correlation coefficients for energy and nutrients stratified by regions. [file 12966_2020_1078_MOESM7_ESM.docx]

**Supplemental Table 6. Pooled crude intraclass correlation coefficients for energy and nutrients stratified by regions ***

|  | Africa | | | Oceania | | | Asia | | | Europe | | | America | | |
| --- | --- | --- | --- | --- | --- | --- | --- | --- | --- | --- | --- | --- | --- | --- | --- |
| Nutrient | ICC (95% CI) | N | *I^2^* | ICC (95% CI) | N | *I^2^* | ICC (95% CI) | N | *I^2^* | ICC (95% CI) | N | *I^2^* | ICC (95% CI) | N | *I^2^* |
| Energy | 0.678 (0.484, 0.808) | 4 | 88.1 | 0.735 (0.632, 0.812) | 7 | 80.9 | 0.683 (0.613, 0.741) | 17 | 93.7 | 0.736 (0.474, 0.878) | 15 | 98.5 | 0.700 (0.616, 0.767) | 18 | 90.7 |
| Carbohydrate | 0.667 (0.527, 0.771) | 4 | 78.2 | 0.733 (0.656, 0.795) | 9 | 80 | 0.616 (0.508, 0.705) | 16 | 96.4 | 0.759 (0.492, 0.895) | 14 | 98.7 | 0.642 (0.566, 0.707) | 19 | 88.4 |
| Protein | 0.653 (0.478, 0.779) | 4 | 84.6 | 0.706 (0.586, 0.795) | 7 | 83.2 | 0.653 (0.598, 0.701) | 16 | 87.7 | 0.640 (0.526, 0.731) | 16 | 91.8 | 0.632 (0.565, 0.691) | 20 | 85.9 |
| Fat | 0.645 (0.444, 0.784) | 4 | 87.4 | 0.707 (0.618, 0.778) | 7 | 71.2 | 0.630 (0.522, 0.718) | 14 | 96.4 | 0.635 (0.531, 0.721) | 11 | 86.3 | 0.632 (0.559, 0.695) | 19 | 85.4 |
| Plant fat | N/A | N/A | N/A | N/A | N/A | N/A | 0.575 (0.370, 0.727) | 3 | 77.4 | 0.519 (0.273, 0.702) | 1 | N/A | 0.570 (0.451, 0.669) | 1 | N/A |
| Animal fat | N/A | N/A | N/A | N/A | N/A | N/A | N/A | N/A | N/A | N/A | N/A | N/A | N/A | N/A | N/A |
| MUFA | 0.700 (0.527, 0.817) | 3 | 81.7 | 0.664 (0.584, 0.730) | 5 | 52.4 | 0.666 (0.602, 0.721) | 6 | 79.4 | 0.584 (0.458, 0.687) | 12 | 88.5 | 0.644 (0.588, 0.694) | 15 | 72 |
| PUFA | 0.676 (0.434, 0.827) | 3 | 88.7 | 0.677 (0.626, 0.722) | 5 | 0 | 0.677 (0.611, 0.734) | 6 | 81.9 | 0.641 (0.354, 0.817) | 13 | 98 | 0.603 (0.537, 0.660) | 18 | 79.6 |
| n-3 PUFA | N/A | N/A | N/A | N/A | N/A | N/A | N/A | N/A | N/A | N/A | N/A | N/A | N/A | N/A | N/A |
| n-6 PUFA | N/A | N/A | N/A | N/A | N/A | N/A | N/A | N/A | N/A | N/A | N/A | N/A | N/A | N/A | N/A |
| SFA | 0.641 (0.413, 0.793) | 4 | 89.8 | 0.741 (0.626, 0.825) | 7 | 85 | 0.725 (0.650, 0.787) | 6 | 89.3 | 0.689 (0.331, 0.873) | 13 | 98.8 | 0.653 (0.594, 0.704) | 19 | 80.5 |
| Linoleic acid | N/A | N/A | N/A | N/A | N/A | N/A | N/A | N/A | N/A | 0.594 (-0.07, 0.894) | 2 | 98 | 0.687 (0.624, 0.742) | 3 | 24.8 |
| Linolenic acid | N/A | N/A | N/A | N/A | N/A | N/A | N/A | N/A | N/A | 0.420 (0.294, 0.531) | 1 | N/A | 0.698 (0.660, 0.733) | 3 | 0 |
| EPA | N/A | N/A | N/A | N/A | N/A | N/A | N/A | N/A | N/A | N/A | N/A | N/A | N/A | N/A | N/A |
| DHA | N/A | N/A | N/A | N/A | N/A | N/A | N/A | N/A | N/A | N/A | N/A | N/A | N/A | N/A | N/A |
| Lipid | N/A | N/A | N/A | N/A | N/A | N/A | 0.557 (0.331, 0.723) | 2 | 59.5 | 0.859 (0.829, 0.885) | 1 | N/A | 0.709 (0.596, 0.795) | 1 | N/A |
| Trans-fat | N/A | N/A | N/A | N/A | N/A | N/A | N/A | N/A | N/A | 0.670 (0.573, 0.748) | 1 | N/A | 0.579 (0.368, 0.733) | 3 | 79.9 |
| Cholesterol | 0.690 (0.574, 0.778) | 1 | N/A | 0.681 (0.631, 0.726) | 5 | 0 | 0.662 (0.579, 0.731) | 11 | 90.7 | 0.658 (0.486, 0.781) | 12 | 94.9 | 0.645 (0.573, 0.706) | 19 | 86.1 |
| Sucrose | N/A | N/A | N/A | 0.639 (0.494, 0.750) | 2 | 68.6 | N/A | N/A | N/A | 0.529 (0.418, 0.625) | 1 | N/A | 0.709 (0.627, 0.776) | 1 | N/A |
| Sugar | N/A | N/A | N/A | 0.727 (0.597, 0.820) | 5 | 75.6 | N/A | N/A | N/A | 0.651 (0.319, 0.841) | 2 | 89 | 0.730 (0.691, 0.764) | 1 | N/A |
| Starch | N/A | N/A | N/A | 0.601 (0.393, 0.750) | 2 | 67.9 | N/A | N/A | N/A | 0.310 (0.174, 0.434) | 1 | N/A | N/A | N/A | N/A |
| Fiber | 0.611 (0.336, 0.790) | 4 | 92 | 0.697 (0.632, 0.752) | 7 | 46 | 0.712 (0.624, 0.782) | 13 | 94.6 | 0.706 (0.575, 0.802) | 11 | 93.7 | 0.652 (0.592, 0.705) | 19 | 83.9 |
| Soluble fiber | N/A | N/A | N/A | N/A | N/A | N/A | N/A | N/A | N/A | N/A | N/A | N/A | N/A | N/A | N/A |
| Insoluble fiber | N/A | N/A | N/A | N/A | N/A | N/A | N/A | N/A | N/A | N/A | N/A | N/A | N/A | N/A | N/A |
| Alcohol | 0.639 (0.491, 0.752) | 1 | N/A | 0.830 (0.762, 0.880) | 7 | 79.7 | 0.820 (0.736, 0.879) | 1 | N/A | 0.753 (0.676, 0.815) | 9 | 76.2 | 0.861 (0.768, 0.918) | 4 | 89.5 |
| Vitamin A | 0.699 (0.381, 0.869) | 3 | 93.8 | 0.676 (0.594, 0.745) | 2 | 0 | 0.596 (0.439, 0.718) | 11 | 97.6 | 0.651 (0.429, 0.799) | 4 | 95.3 | 0.592 (0.503, 0.668) | 7 | 61.8 |
| Retinol | 0.369 (0.166, 0.543) | 1 | N/A | 0.667 (0.573, 0.744) | 2 | 0 | 0.584 (0.388, 0.729) | 4 | 94.4 | 0.562 (0.461, 0.648) | 8 | 61.2 | 0.675 (0.583, 0.749) | 3 | 31.5 |
| Carotene | N/A | N/A | N/A | N/A | N/A | N/A | 0.626 (0.480, 0.739) | 8 | 97.5 | 0.670 (0.573, 0.748) | 1 | N/A | N/A | N/A | N/A |
| β-Carotene | 0.590 (0.427, 0.715) | 1 | N/A | 0.670 (0.618, 0.715) | 5 | 0 | 0.839 (0.781, 0.883) | 1 | N/A | 0.627 (0.484, 0.737) | 7 | 79.1 | 0.703 (0.666, 0.738) | 5 | 24.4 |
| Vitamin C | 0.629 (0.163, 0.865) | 3 | 96 | 0.759 (0.661, 0.832) | 5 | 79.8 | 0.658 (0.544, 0.749) | 15 | 97.3 | 0.667 (0.416, 0.823) | 12 | 97.6 | 0.632 (0.562, 0.692) | 12 | 78.3 |
| Vitamin D | N/A | N/A | N/A | 0.649 (0.482, 0.771) | 1 | N/A | 0.724 (0.549, 0.838) | 3 | 98.7 | 0.818 (0.267, 0.966) | 4 | 99.4 | 0.563 (0.478, 0.638) | 8 | 77.3 |
| Vitamin E | 0.703 (0.508, 0.829) | 3 | 85.3 | 0.659 (0.550, 0.746) | 4 | 68.7 | 0.632 (0.515, 0.725) | 14 | 96.9 | 0.796 (0.335, 0.949) | 5 | 99.2 | 0.596 (0.502, 0.677) | 8 | 79.6 |
| Vitamin K | N/A | N/A | N/A | N/A | N/A | N/A | 0.400 (0.358, 0.439) | 1 | N/A | N/A | N/A | N/A | 0.713 (0.676, 0.746) | 3 | 0 |
| Thiamin | 0.829 (0.752, 0.884) | 1 | N/A | 0.649 (0.514, 0.752) | 4 | 78.2 | 0.642 (0.580, 0.697) | 11 | 89.2 | 0.585 (0.418, 0.714) | 6 | 91.7 | 0.603 (0.499, 0.689) | 9 | 83.8 |
| Riboflavin | N/A | N/A | N/A | 0.690 (0.633, 0.738) | 5 | 21 | 0.694 (0.610, 0.762) | 10 | 95.2 | 0.731 (0.557, 0.843) | 4 | 94.7 | 0.573 (0.471, 0.660) | 9 | 80.2 |
| Niacin | N/A | N/A | N/A | 0.645 (0.554, 0.720) | 4 | 54.2 | 0.634 (0.500, 0.739) | 7 | 93.3 | 0.744 (0.599, 0.841) | 4 | 89.2 | 0.664 (0.584, 0.731) | 7 | 74.6 |
| Vitamin B6 | N/A | N/A | N/A | 0.679 (0.616, 0.733) | 3 | 0 | 0.510 (0.334, 0.651) | 1 | N/A | 0.848 (0.308, 0.974) | 4 | 99.3 | 0.639 (0.547, 0.715) | 5 | 78.4 |
| Folate | 0.421 (0.301, 0.527) | 2 | N/A | 0.741 (0.673, 0.796) | 3 | 34.5 | 0.717 (0.654, 0.770) | 5 | 91.2 | 0.575 (0.338, 0.743) | 4 | 87.3 | 0.614 (0.517, 0.696) | 11 | 86.2 |
| Vitamin B12 | N/A | N/A | N/A | 0.635 (0.558, 0.701) | 2 | 0 | 0.529 (0.358, 0.666) | 1 | N/A | 0.802 (0.169, 0.966) | 3 | 99.3 | 0.634 (0.533, 0.717) | 7 | 86.3 |
| Se | 0.736 (0.566, 0.846) | 2 | 77.2 | 0.636 (0.540, 0.717) | 2 | 33.9 | 0.656 (0.465, 0.788) | 3 | 88 | 0.670 (0.573, 0.748) | 1 | N/A | 0.629 (0.508, 0.726) | 3 | 50.7 |
| Mg | 0.669 (0.335, 0.853) | 2 | 90.3 | 0.649 (0.590, 0.700) | 4 | 0 | 0.648 (0.539, 0.735) | 5 | 73.6 | 0.753 (0.414, 0.908) | 3 | 96.1 | 0.676 (0.500, 0.799) | 5 | 93 |
| Ca | 0.654 (0.490, 0.774) | 4 | 82.9 | 0.627 (0.529, 0.709) | 5 | 63.3 | 0.682 (0.611, 0.742) | 14 | 93.7 | 0.582 (0.504, 0.650) | 10 | 66.5 | 0.621 (0.533, 0.695) | 19 | 91.2 |
| Fe | 0.716 (0.548, 0.829) | 3 | 82.2 | 0.645 (0.531, 0.736) | 4 | 70.1 | 0.668 (0.525, 0.774) | 10 | 97.9 | 0.539 (0.445, 0.621) | 8 | 68 | 0.646 (0.569, 0.712) | 14 | 85.4 |
| I | N/A | N/A | N/A | N/A | N/A | N/A | N/A | N/A | N/A | N/A | N/A | N/A | N/A | N/A | N/A |
| Zn | 0.487 (0.244, 0.673) | 2 | 76.3 | 0.560 (0.486, 0.627) | 3 | 0 | 0.573 (0.489, 0.646) | 6 | 74.8 | 0.626 (0.553, 0.688) | 5 | 0 | 0.634 (0.559, 0.698) | 10 | 80.6 |
| Cu | N/A | N/A | N/A | N/A | N/A | N/A | 0.682 (0.587, 0.758) | 2 | 0 | 0.670 (0.573, 0.748) | 1 | N/A | 0.649 (0.602, 0.692) | 1 | N/A |
| K | 0.560 (0.412, 0.678) | 1 | N/A | 0.694 (0.611, 0.762) | 3 | 42.6 | 0.728 (0.564, 0.837) | 6 | 98.4 | 0.602 (0.303, 0.793) | 5 | 95.4 | 0.671 (0.586, 0.741) | 10 | 82 |
| P | N/A | N/A | N/A | 0.639 (0.509, 0.741) | 1 | N/A | 0.685 (0.416, 0.844) | 5 | 96.8 | 0.526 (0.448, 0.596) | 7 | 19.5 | 0.597 (0.480, 0.694) | 10 | 89 |
| Na | 0.499 (0.340, 0.630) | 1 | N/A | 0.712 (0.650, 0.764) | 4 | 24.5 | 0.520 (0.360, 0.651) | 5 | 86.6 | 0.796 (-0.15, 0.981) | 4 | 99.5 | 0.625 (0.523, 0.710) | 11 | 87.1 |
| Mn | N/A | N/A | N/A | N/A | N/A | N/A | N/A | N/A | N/A | N/A | N/A | N/A | N/A | N/A | N/A |

* CI, confidence interval; N/A: not available
