# Supplementary material for: A meta-analysis of the reproducibility of food frequency questionnaires in nutritional epidemiological studies
Source: Int J Behav Nutr Phys Act. 2021 Jan 11;18:12. doi: 10.1186/s12966-020-01078-4 (PMC7802360; doi:10.1186/s12966-020-01078-4)
Supplement: Supplementary file 8 — Additional file 8 Supplemental Table 7. Pooled energy-adjusted intraclass correlation coefficients for energy and nutrients stratified by regions. [file 12966_2020_1078_MOESM8_ESM.docx]

**Supplemental Table 7. Pooled energy-adjusted intraclass correlation coefficients for energy and nutrients stratified by regions***

| Nutrient | Africa | | | Oceania | | | Asia | | | Europe | | | America | | |
| --- | --- | --- | --- | --- | --- | --- | --- | --- | --- | --- | --- | --- | --- | --- | --- |
|  | ICC (95% CI) | N | *I^2^* | ICC (95% CI) | N | *I^2^* | ICC (95% CI) | N | *I^2^* | ICC (95% CI) | N | *I^2^* | ICC (95% CI) | N | *I^2^* |
| Energy | N/A | N/A | N/A | N/A | N/A | N/A | N/A | N/A | N/A | N/A | N/A | N/A | N/A | N/A | N/A |
| Carbohydrate | 0.649 (0.476, 0.773) | 2 | 68.5 | N/A | N/A | N/A | 0.548 (0.416, 0.657) | 7 | 84 | 0.836 (0.791, 0.872) | 4 | 36.1 | 0.614 (0.528, 0.688) | 1 | N/A |
| Protein | 0.697 (0.572, 0.790) | 2 | 53.8 | N/A | N/A | N/A | 0.493 (0.425, 0.556) | 7 | 36.8 | 0.698 (0.609, 0.770) | 6 | 66.8 | 0.591 (0.523, 0.652) | 10 | 69.2 |
| Fat | 0.610 (0.416, 0.750) | 2 | 71.1 | N/A | N/A | N/A | 0.484 (0.288, 0.641) | 5 | 90 | 0.730 (0.648, 0.795) | 1 | N/A | 0.571 (0.472, 0.656) | 11 | 82.4 |
| Plant fat | N/A | N/A | N/A | N/A | N/A | N/A | N/A | N/A | N/A | N/A | N/A | N/A | N/A | N/A | N/A |
| Animal fat | N/A | N/A | N/A | N/A | N/A | N/A | N/A | N/A | N/A | N/A | N/A | N/A | N/A | N/A | N/A |
| MUFA | 0.711 (0.598, 0.796) | 2 | 48.5 | N/A | N/A | N/A | 0.537 (0.074, 0.809) | 2 | 91.8 | 0.671 (0.542, 0.769) | 6 | 81 | 0.592 (0.498, 0.671) | 8 | 74.3 |
| PUFA | 0.715 (0.615, 0.793) | 2 | 38.1 | N/A | N/A | N/A | 0.527 (0.176, 0.758) | 2 | 86.5 | 0.594 (0.401, 0.736) | 6 | 88.3 | 0.513 (0.405, 0.606) | 8 | 75.7 |
| n-3 PUFA | N/A | N/A | N/A | N/A | N/A | N/A | N/A | N/A | N/A | N/A | N/A | N/A | N/A | N/A | N/A |
| n-6 PUFA | N/A | N/A | N/A | N/A | N/A | N/A | N/A | N/A | N/A | N/A | N/A | N/A | N/A | N/A | N/A |
| SFA | 0.641 (0.413, 0.793) | 4 | 89.8 | 0.741 (0.626, 0.825) | 7 | 85 | 0.725 (0.650, 0.787) | 6 | 89.3 | 0.689 (0.331, 0.873) | 13 | 98.8 | 0.653 (0.594, 0.704) | 19 | 80.5 |
| Linoleic acid | N/A | N/A | N/A | N/A | N/A | N/A | N/A | N/A | N/A | 0.750 (0.673, 0.810) | 1 | N/A | 0.635 (0.588, 0.677) | 2 | 0 |
| Linolenic acid | N/A | N/A | N/A | N/A | N/A | N/A | N/A | N/A | N/A | N/A | N/A | N/A | N/A | N/A | N/A |
| EPA | N/A | N/A | N/A | N/A | N/A | N/A | N/A | N/A | N/A | N/A | N/A | N/A | N/A | N/A | N/A |
| DHA | N/A | N/A | N/A | N/A | N/A | N/A | N/A | N/A | N/A | N/A | N/A | N/A | N/A | N/A | N/A |
| Lipid | N/A | N/A | N/A | N/A | N/A | N/A | 0.590 (0.459, 0.695) | 2 | 0 | 0.849 (0.817, 0.876) | 1 | N/A | 0.499 (0.336, 0.633) | 1 | N/A |
| Trans-fat | N/A | N/A | N/A | N/A | N/A | N/A | N/A | N/A | N/A | N/A | N/A | N/A | N/A | N/A | N/A |
| Cholesterol | 0.539 (0.388, 0.662) | 1 | N/A | N/A | N/A | N/A | 0.489 (0.401, 0.566) | 5 | 29 | 0.704 (0.624, 0.769) | 7 | 73.4 | 0.623 (0.545, 0.690) | 12 | 78.1 |
| Sucrose | N/A | N/A | N/A | N/A | N/A | N/A | N/A | N/A | N/A | N/A | N/A | N/A | N/A | N/A | N/A |
| Sugar | N/A | N/A | N/A | N/A | N/A | N/A | N/A | N/A | N/A | N/A | N/A | N/A | N/A | N/A | N/A |
| Starch | N/A | N/A | N/A | N/A | N/A | N/A | N/A | N/A | N/A | N/A | N/A | N/A | N/A | N/A | N/A |
| Fiber | 0.669 (0.288, 0.866) | 2 | 92.2 | N/A | N/A | N/A | 0.571 (0.420, 0.692) | 7 | 88.4 | 0.781 (0.666, 0.859) | 4 | 84.2 | 0.687 (0.585, 0.768) | 8 | 87.9 |
| Soluble fiber | N/A | N/A | N/A | N/A | N/A | N/A | N/A | N/A | N/A | N/A | N/A | N/A | N/A | N/A | N/A |
| Insoluble fiber | N/A | N/A | N/A | N/A | N/A | N/A | N/A | N/A | N/A | N/A | N/A | N/A | N/A | N/A | N/A |
| Alcohol | N/A | N/A | N/A | N/A | N/A | N/A | 0.800 (0.707, 0.865) | 1 | N/A | 0.791 (0.721, 0.845) | 5 | 51.2 | 0.828 (0.641, 0.922) | 3 | 94.8 |
| Vitamin A | 0.756 (0.488, 0.894) | 2 | 90.1 | N/A | N/A | N/A | 0.460 (0.294, 0.599) | 5 | 77.2 | 0.704 (0.202, 0.913) | 2 | 98 | 0.583 (0.340, 0.753) | 3 | 86.6 |
| Retinol | N/A | N/A | N/A | N/A | N/A | N/A | 0.479 (0.325, 0.608) | 3 | 74 | 0.517 (0.271, 0.699) | 4 | 72.2 | 0.652 (0.401, 0.811) | 2 | 73.3 |
| Carotene | N/A | N/A | N/A | N/A | N/A | N/A | 0.512 (0.328, 0.658) | 5 | 86.2 | N/A | N/A | N/A | N/A | N/A | N/A |
| β-Carotene | N/A | N/A | N/A | N/A | N/A | N/A | N/A | N/A | N/A | 0.544 (0.197, 0.771) | 4 | 85.8 | 0.690 (0.594, 0.765) | 2 | 51.1 |
| Vitamin C | 0.672 (0.258, 0.878) | 2 | 93.3 | N/A | N/A | N/A | 0.527 (0.331, 0.678) | 8 | 93.6 | 0.716 (0.506, 0.845) | 6 | 93.5 | 0.665 (0.531, 0.766) | 6 | 88.6 |
| Vitamin D | N/A | N/A | N/A | N/A | N/A | N/A | N/A | N/A | N/A | 0.827 (0.458, 0.953) | 2 | 98.1 | 0.514 (0.259, 0.702) | 3 | 93.9 |
| Vitamin E | 0.617 (0.226, 0.837) | 2 | 91.5 | N/A | N/A | N/A | 0.529 (0.362, 0.662) | 7 | 89.2 | 0.820 (0.666, 0.907) | 2 | 92.9 | 0.573 (0.379, 0.718) | 4 | 89.4 |
| Vitamin K | N/A | N/A | N/A | N/A | N/A | N/A | N/A | N/A | N/A | N/A | N/A | N/A | N/A | N/A | N/A |
| Thiamin | 0.750 (0.642, 0.828) | 1 | N/A | 0.527 (0.331, 0.680) | 5 | 91.2 | N/A | N/A | N/A | 0.762 (0.267, 0.939) | 2 | 98.3 | 0.566 (0.428, 0.678) | 4 | 81.1 |
| Riboflavin | N/A | N/A | N/A | 0.435 (0.294, 0.558) | 4 | 77.2 | N/A | N/A | N/A | 0.833 (0.777, 0.876) | 2 | 66.5 | 0.642 (0.550, 0.720) | 4 | 63.1 |
| Niacin | N/A | N/A | N/A | 0.570 (0.343, 0.734) | 5 | 94.5 | N/A | N/A | N/A | 0.739 (0.661, 0.802) | 1 | N/A | 0.607 (0.494, 0.699) | 4 | 75.5 |
| Vitamin B6 | N/A | N/A | N/A | 0.459 (0.274, 0.611) | 1 | N/A | N/A | N/A | N/A | 0.859 (0.829, 0.884) | 2 | 26 | 0.615 (0.567, 0.657) | 2 | 0 |
| Folate | 0.420 (0.248, 0.566) | 1 | N/A | N/A | N/A | N/A | 0.450 (0.263, 0.604) | 1 | N/A | N/A | N/A | N/A | 0.658 (0.561, 0.738) | 4 | 69.9 |
| Vitamin B12 | N/A | N/A | N/A | N/A | N/A | N/A | 0.539 (0.370, 0.674) | 1 | N/A | 0.822 (0.501, 0.944) | 2 | 97.6 | 0.614 (0.489, 0.714) | 4 | 83.8 |
| Se | 0.621 (0.243, 0.835) | 2 | 91.1 | N/A | N/A | N/A | N/A | N/A | N/A | N/A | N/A | N/A | 0.551 (0.399, 0.674) | 2 | 52.3 |
| Mg | 0.649 (0.410, 0.805) | 2 | 82.2 | N/A | N/A | N/A | 0.679 (0.551, 0.777) | 1 | N/A | N/A | N/A | N/A | 0.575 (0.331, 0.747) | 3 | 94 |
| Ca | 0.643 (0.210, 0.865) | 2 | 93.2 | N/A | N/A | N/A | 0.628 (0.492, 0.734) | 8 | 91.1 | 0.619 (0.537, 0.691) | 5 | 27.2 | 0.676 (0.505, 0.796) | 8 | 95.1 |
| Fe | 0.698 (0.385, 0.866) | 2 | 90.2 | N/A | N/A | N/A | 0.490 (0.294, 0.646) | 4 | 86.2 | 0.492 (0.390, 0.581) | 5 | 27.7 | 0.593 (0.514, 0.662) | 8 | 66.5 |
| I | N/A | N/A | N/A | N/A | N/A | N/A | N/A | N/A | N/A | N/A | N/A | N/A | N/A | N/A | N/A |
| Zn | 0.459 (0.294, 0.598) | 1 | N/A | N/A | N/A | N/A | 0.436 (0.278, 0.571) | 2 | 51 | 0.597 (0.480, 0.693) | 4 | 12.5 | 0.626 (0.516, 0.716) | 5 | 78.6 |
| Cu | N/A | N/A | N/A | N/A | N/A | N/A | N/A | N/A | N/A | N/A | N/A | N/A | N/A | N/A | N/A |
| K | 0.400 (0.226, 0.549) | 1 | N/A | N/A | N/A | N/A | 0.709 (0.590, 0.799) | 1 | N/A | 0.300 (0.200, 0.393) | 1 | N/A | 0.716 (0.660, 0.763) | 4 | 42.4 |
| P | N/A | N/A | N/A | N/A | N/A | N/A | 0.579 (0.481, 0.664) | 1 | N/A | 0.686 (0.596, 0.760) | 4 | N/A | 0.613 (0.427, 0.749) | 4 | 92.2 |
| Na | 0.670 (0.549, 0.763) | 1 | N/A | N/A | N/A | N/A | 0.200 (0.066, 0.326) | 1 | N/A | 0.890 (0.865, 0.910) | 1 | N/A | 0.669 (0.530, 0.773) | 5 | 88.9 |
| Mn | N/A | N/A | N/A | N/A | N/A | N/A | N/A | N/A | N/A | N/A | N/A | N/A | N/A | N/A | N/A |

* CI, confidence interval; N/A: not available
