# Supplementary material for: A meta-analysis of the reproducibility of food frequency questionnaires in nutritional epidemiological studies
Source: Int J Behav Nutr Phys Act. 2021 Jan 11;18:12. doi: 10.1186/s12966-020-01078-4 (PMC7802360; doi:10.1186/s12966-020-01078-4)
Supplement: Supplementary file 9 — Additional file 9 Supplemental Table 8. Pooled crude spearman correlation coefficients for energy and nutrients stratified by regions. [file 12966_2020_1078_MOESM9_ESM.docx]

**Supplemental Table 8. Pooled crude spearman correlation coefficients for energy and nutrients stratified by regions ***

| Nutrient | Africa | | | Oceania | | | Asia | | | Europe | | | America | | |
| --- | --- | --- | --- | --- | --- | --- | --- | --- | --- | --- | --- | --- | --- | --- | --- |
|  | SCC (95% CI) | N | I^2^ | SCC (95% CI) | N | I^2^ | SCC (95% CI) | N | I^2^ | SCC (95% CI) | N | I^2^ | SCC (95% CI) | N | I^2^ |
| Energy | 0.486 (0.303, 0.486) | 6 | 86.2 | 0.720 (0.555, 0.720) | 4 | 90.1 | 0.633 (0.594, 0.633) | 36 | 83.3 | 0.721 (0.685, 0.721) | 36 | 77.6 | 0.581 (0.521, 0.581) | 24 | 81.8 |
| Protein | 0.452 (0.278, 0.452) | 6 | 84 | 0.632 (0.537, 0.632) | 4 | 62 | 0.633 (0.599, 0.633) | 37 | 79 | 0.670 (0.634, 0.670) | 35 | 70.5 | 0.505 (0.452, 0.505) | 24 | 72.1 |
| Fat | 0.532 (0.372, 0.532) | 6 | 84.2 | 0.683 (0.548, 0.683) | 4 | 83.5 | 0.616 (0.584, 0.616) | 36 | 72.3 | 0.676 (0.637, 0.676) | 34 | 75.4 | 0.566 (0.510, 0.566) | 24 | 77.8 |
| Plant fat | N/A | N/A | N/A | N/A | N/A | N/A | 0.730 (0.585, 0.730) | 1 | N/A | 0.487 (0.437, 0.487) | 3 | 0 | 0.576 (0.347, 0.576) | 2 | 70.3 |
| Animal fat | N/A | N/A | N/A | N/A | N/A | N/A | 0.690 (0.530, 0.690) | 1 | N/A | 0.683 (0.646, 0.683) | 2 | 0 | 0.750 (0.673, 0.750) | 1 | N/A |
| MUFA | 0.632 (0.533, 0.632) | 4 | 88.1 | 0.680 (0.555, 0.680) | 7 | 80.9 | 0.603 (0.553, 0.603) | 17 | 93.7 | 0.624 (0.566, 0.624) | 15 | 98.5 | 0.573 (0.516, 0.573) | 18 | 90.7 |
| PUFA | 0.585 (0.470, 0.585) | 3 | 43.5 | 0.660 (0.498, 0.660) | 4 | 86.9 | 0.616 (0.573, 0.616) | 15 | 60.6 | 0.626 (0.579, 0.626) | 19 | 71.8 | 0.510 (0.446, 0.510) | 16 | 67.2 |
| n-3 PUFA | N/A | N/A | N/A | N/A | N/A | N/A | 0.619 (0.573, 0.619) | 6 | 59.8 | N/A | N/A | N/A | N/A | N/A | N/A |
| n-6 PUFA | N/A | N/A | N/A | N/A | N/A | N/A | 0.594 (0.563, 0.594) | 6 | 18.3 | N/A | N/A | N/A | N/A | N/A | N/A |
| SFA | 0.631 (0.536, 0.631) | 3 | 32.1 | 0.665 (0.532, 0.665) | 4 | 81.9 | 0.652 (0.606, 0.652) | 15 | 70.4 | 0.629 (0.583, 0.629) | 21 | 71.5 | 0.590 (0.529, 0.590) | 22 | 82.7 |
| Linoleic acid | N/A | N/A | N/A | N/A | N/A | N/A | 0.789 (0.704, 0.789) | 1 | N/A | 0.659 (0.624, 0.659) | 3 | 0 | 0.534 (0.491, 0.534) | 5 | 8.4 |
| Linolenic acid | N/A | N/A | N/A | N/A | N/A | N/A | 0.809 (0.731, 0.809) | 1 | N/A | 0.621 (0.580, 0.621) | 2 | 0 | N/A | 1 | N/A |
| EPA | N/A | N/A | N/A | N/A | N/A | N/A | 0.789 (0.704, 0.789) | 1 | N/A | 0.775 (0.236, 0.775) | 2 | 93.2 | N/A | N/A | N/A |
| DHA | N/A | N/A | N/A | N/A | N/A | N/A | 0.789 (0.704, 0.789) | 1 | N/A | 0.709 (0.378, 0.709) | 2 | 82.1 | N/A | N/A | N/A |
| Trans-fat | N/A | N/A | N/A | N/A | N/A | N/A | N/A | N/A | N/A | 0.524 (0.360, 0.524) | 2 | 0 | 0.645 (0.385, 0.645) | 4 | 94.4 |
| Cholesterol | 0.632 (0.501, 0.632) | 1 | N/A | 0.679 (0.625, 0.679) | 3 | 0 | 0.633 (0.573, 0.633) | 20 | 85.5 | 0.621 (0.573, 0.621) | 21 | 70.4 | 0.577 (0.514, 0.577) | 21 | 82 |
| Carbohydrate | 0.510 (0.275, 0.510) | 6 | 91.7 | 0.758 (0.655, 0.758) | 3 | 76.2 | 0.603 (0.554, 0.603) | 36 | 88.3 | 0.709 (0.669, 0.709) | 33 | 80.2 | 0.588 (0.532, 0.588) | 23 | 80.1 |
| Sucrose | N/A | N/A | N/A | N/A | N/A | N/A | N/A | N/A | N/A | 0.738 (0.688, 0.738) | 5 | 40.4 | 0.570 (0.207, 0.570) | 2 | 85.8 |
| Sugar | N/A | N/A | N/A | 0.685 (0.476, 0.685) | 4 | 92.4 | N/A | N/A | N/A | 0.691 (0.650, 0.691) | 7 | 29.5 | N/A | N/A | N/A |
| Starch | N/A | N/A | N/A | N/A | N/A | N/A | N/A | N/A | N/A | 0.641 (0.604, 0.641) | 4 | 0 | N/A | N/A | N/A |
| Fiber | 0.573 (0.326, 0.573) | 4 | 90.3 | 0.697 (0.519, 0.697) | 4 | 90.5 | 0.642 (0.590, 0.642) | 26 | 86.2 | 0.696 (0.657, 0.696) | 31 | 73.6 | 0.545 (0.476, 0.545) | 22 | 82 |
| Soluble fiber | 0.462 (0.297, 0.462) | 1 | N/A | N/A | N/A | N/A | 0.648 (0.595, 0.648) | 11 | 73.5 | 0.825 (0.754, 0.825) | 2 | 0 | N/A | N/A | N/A |
| Insoluble fiber | 0.462 (0.297, 0.462) | 1 | N/A | N/A | N/A | N/A | 0.637 (0.597, 0.637) | 9 | 0 | 0.796 (0.692, 0.796) | 2 | 29.8 | N/A | N/A | N/A |
| Lipid | N/A | N/A | N/A | N/A | N/A | N/A | 0.537 (0.460, 0.537) | 2 | 0 | 0.589 (0.483, 0.589) | 3 | 0 | 0.532 (0.374, 0.532) | 1 | N/A |
| Alcohol | 0.715 (0.645, 0.715) | 2 | 0 | 0.907 (0.799, 0.907) | 3 | 94.2 | 0.806 (0.698, 0.806) | 8 | 93.1 | 0.857 (0.819, 0.857) | 28 | 93.5 | 0.872 (0.781, 0.872) | 6 | 94.1 |
| Vitamin A | 0.638 (0.321, 0.638) | 3 | 93.3 | 0.514 (0.399, 0.514) | 3 | 38.8 | 0.554 (0.501, 0.554) | 15 | 80.3 | 0.756 (0.647, 0.756) | 7 | 90.7 | 0.612 (0.521, 0.612) | 14 | 86.1 |
| Retinol | 0.410 (0.211, 0.410) | 1 | N/A | 0.559 (0.289, 0.559) | 4 | 93 | 0.584 (0.533, 0.584) | 22 | 78.1 | 0.615 (0.566, 0.615) | 15 | 65.4 | 0.463 (0.333, 0.463) | 7 | 83.2 |
| Carotene | N/A | N/A | N/A | 0.718 (0.564, 0.718) | 2 | 83.6 | 0.583 (0.515, 0.583) | 17 | 90.4 | 0.654 (0.569, 0.654) | 4 | 85.2 | 0.542 (0.471, 0.542) | 2 | 39.3 |
| β-Carotene | 0.570 (0.402, 0.570) | 1 | N/A | 0.528 (0.411, 0.528) | 2 | 0 | 0.586 (0.543, 0.586) | 16 | 50.4 | 0.676 (0.601, 0.676) | 13 | 75.5 | 0.566 (0.424, 0.566) | 7 | 83.4 |
| Vitamin C | 0.561 (0.245, 0.561) | 4 | 93 | 0.688 (0.545, 0.688) | 4 | 85.5 | 0.610 (0.558, 0.610) | 31 | 88.8 | 0.658 (0.621, 0.658) | 29 | 64.2 | 0.593 (0.521, 0.593) | 24 | 87 |
| Vitamin D | 0.346 (0.047, 0.346) | 2 | 78.7 | N/A | N/A | N/A | 0.661 (0.638, 0.661) | 8 | 19.3 | 0.681 (0.612, 0.681) | 11 | 71.9 | 0.552 (0.409, 0.552) | 9 | 87.5 |
| Vitamin E | 0.691 (0.624, 0.691) | 3 | 0 | N/A | N/A | N/A | 0.620 (0.518, 0.620) | 15 | 95.8 | 0.638 (0.589, 0.638) | 19 | 73.8 | 0.596 (0.496, 0.596) | 15 | 91.2 |
| Vitamin K | N/A | N/A | N/A | N/A | N/A | N/A | 0.656 (0.629, 0.656) | 4 | 0 | 0.583 (0.156, 0.583) | 2 | 49.2 | 0.413 (0.231, 0.413) | 1 | N/A |
| Thiamin | 0.693 (0.567, 0.693) | 1 | N/A | 0.678 (0.495, 0.678) | 4 | 90.1 | 0.604 (0.570, 0.604) | 27 | 71.6 | 0.629 (0.570, 0.629) | 10 | 67.2 | 0.552 (0.485, 0.552) | 13 | 58.8 |
| Riboflavin | N/A | N/A | N/A | 0.677 (0.531, 0.677) | 4 | 85.5 | 0.643 (0.606, 0.643) | 29 | 79.5 | 0.693 (0.648, 0.693) | 9 | 65.3 | 0.555 (0.444, 0.555) | 12 | 82.6 |
| Niacin | N/A | N/A | N/A | 0.594 (0.387, 0.594) | 2 | 68.8 | 0.646 (0.548, 0.646) | 25 | 95.9 | 0.804 (0.489, 0.804) | 3 | 94.4 | 0.579 (0.483, 0.579) | 9 | 73 |
| Vitamin B6 | 0.283 (0.033, 0.283) | 2 | 68.2 | N/A | N/A | N/A | 0.619 (0.547, 0.619) | 10 | 61 | 0.724 (0.642, 0.724) | 5 | 63.7 | 0.588 (0.496, 0.588) | 10 | 75.7 |
| Folate | 0.371 (0.180, 0.371) | 3 | 68.8 | 0.624 (0.195, 0.624) | 2 | 91.6 | 0.612 (0.561, 0.612) | 19 | 83.4 | 0.685 (0.612, 0.685) | 12 | 75.9 | 0.595 (0.521, 0.595) | 13 | 75.7 |
| Vitamin B12 | 0.385 (0.255, 0.385) | 2 | 0 | N/A | N/A | N/A | 0.611 (0.541, 0.611) | 8 | 48.6 | 0.748 (0.660, 0.748) | 8 | 81 | 0.594 (0.500, 0.594) | 10 | 77.4 |
| Se | 0.682 (0.598, 0.682) | 2 | 0 | N/A | N/A | N/A | 0.644 (0.546, 0.644) | 8 | 88.2 | 0.838 (0.739, 0.838) | 1 | N/A | 0.584 (0.516, 0.584) | 4 | 0 |
| Mg | 0.475 (0.312, 0.475) | 4 | 72.7 | 0.687 (0.117, 0.687) | 2 | 95.5 | 0.717 (0.616, 0.717) | 11 | 90.3 | 0.759 (0.595, 0.759) | 6 | 85.9 | 0.599 (0.422, 0.599) | 7 | 91.6 |
| Ca | 0.522 (0.341, 0.522) | 5 | 82.9 | 0.652 (0.532, 0.652) | 4 | 76.9 | 0.660 (0.623, 0.660) | 31 | 80.7 | 0.654 (0.610, 0.654) | 24 | 71.1 | 0.544 (0.467, 0.544) | 23 | 86.1 |
| Fe | 0.534 (0.303, 0.534) | 5 | 89.4 | 0.687 (0.515, 0.687) | 4 | 89.4 | 0.630 (0.584, 0.630) | 27 | 85.7 | 0.679 (0.640, 0.679) | 22 | 63.9 | 0.481 (0.411, 0.481) | 17 | 67.9 |
| Zn | 0.521 (0.367, 0.521) | 1 | N/A | 0.638 (0.539, 0.638) | 4 | 65.2 | 0.611 (0.494, 0.611) | 11 | 91.2 | 0.720 (0.574, 0.720) | 2 | 61.5 | 0.613 (0.530, 0.613) | 8 | 64.7 |
| Cu | N/A | N/A | N/A | N/A | N/A | N/A | 0.731 (0.469, 0.731) | 3 | 91.1 | 0.838 (0.739, 0.838) | 1 | N/A | 0.719 (0.408, 0.719) | 2 | 90.9 |
| K | 0.542 (0.391, 0.542) | 1 | N/A | 0.713 (0.575, 0.713) | 1 | N/A | 0.649 (0.610, 0.649) | 27 | 80.5 | 0.656 (0.548, 0.656) | 10 | 86.1 | 0.584 (0.509, 0.584) | 10 | 64.3 |
| P | N/A | N/A | N/A | 0.734 (0.603, 0.734) | 1 | N/A | 0.665 (0.621, 0.665) | 25 | 79.9 | 0.568 (0.332, 0.568) | 5 | 78.2 | 0.515 (0.391, 0.515) | 12 | 84.4 |
| Na | 0.723 (0.618, 0.723) | 1 | N/A | 0.744 (0.617, 0.744) | 1 | N/A | 0.636 (0.571, 0.636) | 22 | 88.2 | 0.613 (0.559, 0.613) | 10 | 58.5 | 0.544 (0.449, 0.544) | 7 | 59.6 |
| Mn | N/A | N/A | N/A | N/A | N/A | N/A | 0.664 (0.586, 0.664) | 3 | 7.2 | 0.703 (0.542, 0.703) | 1 | N/A | 0.601 (0.455, 0.601) | 1 | N/A |

* CI, confidence interval; N/A: not available
