# Supplementary material for: A meta-analysis of the reproducibility of food frequency questionnaires in nutritional epidemiological studies
Source: Int J Behav Nutr Phys Act. 2021 Jan 11;18:12. doi: 10.1186/s12966-020-01078-4 (PMC7802360; doi:10.1186/s12966-020-01078-4)
Supplement: Supplementary file 10 — Additional file 10 Supplemental Table 9. Pooled energy-adjusted spearman correlation coefficients for energy and nutrients stratified by regions. [file 12966_2020_1078_MOESM10_ESM.docx]

**Supplemental Table 9. Pooled energy-adjusted spearman correlation coefficients for energy and nutrients stratified by regions***

| Nutrient | Africa | | | Oceania | | | Asia | | | Europe | | | America | | |
| --- | --- | --- | --- | --- | --- | --- | --- | --- | --- | --- | --- | --- | --- | --- | --- |
|  | SCC (95% CI) | N | I^2^ | SCC (95% CI) | N | I^2^ | SCC (95% CI) | N | I^2^ | SCC (95% CI) | N | I^2^ | SCC (95% CI) | N | I^2^ |
| Energy | N/A | N/A | N/A | N/A | N/A | N/A | N/A | N/A | N/A | N/A | N/A | N/A | N/A | N/A | N/A |
| Protein | 0.585 (0.476, 0.676) | 27 | 79.3 | 0.346 (0.163, 0.553) | 19 | 74.2 | 0.534 (0.476, 0.587) | 15 | 69.1 | 0.661 (0.662, 0.779) | 19 | 74.2 | 0.476 (0.413, 0.535) | 15 | 69.1 |
| Fat | 0.545 (0.438, 0.643) | 23 | 81.5 | 0.296 (0.115, 0.462) | 16 | 80 | 0.532 (0.464, 0.593) | 14 | 77.3 | 0.653 (0.587, 0.799) | 16 | 80 | 0.499 (0.426, 0.567) | 14 | 77.3 |
| Plant fat | N/A | N/A | N/A | N/A | N/A | N/A | N/A | N/A | N/A | N/A | N/A | N/A | N/A | N/A | N/A |
| Animal fat | N/A | N/A | N/A | N/A | N/A | N/A | N/A | N/A | N/A | N/A | N/A | N/A | N/A | N/A | N/A |
| MUFA | 0.598 (0.483, 0.681) | 11 | 87.2 | 0.316 (0.132, 0.481) | 7 | 80 | 0.551 (0.424, 0.657) | 11 | 67 | 0.621 (0.513, 0.799) | 7 | 80 | 0.519 (0.444, 0.587) | 11 | 67 |
| PUFA | 0.563 (0.452, 0.658) | 11 | 84 | 0.316 (0.132, 0.481) | 9 | 62.2 | 0.518 (0.411, 0.619) | 8 | 54.9 | 0.675 (0.547, 0.661) | 9 | 62.2 | 0.428 (0.343, 0.557) | 8 | 54.9 |
| n-3 PUFA | N/A | 5 | 36.9 | N/A | N/A | N/A | 0.469 (0.428, 0.532) | N/A | N/A | N/A | N/A | N/A | N/A | N/A | N/A |
| n-6 PUFA | N/A | 5 | 57.4 | N/A | N/A | N/A | 0.441 (0.355, 0.519) | N/A | N/A | N/A | N/A | N/A | N/A | N/A | N/A |
| SFA | 0.611 (0.579, 0.698) | 11 | 83.9 | 0.336 (0.153, 0.496) | 9 | 65.9 | 0.611 (0.497, 0.687) | 14 | 84.5 | 0.582 (0.516, 0.642) | 9 | 65.9 | 0.537 (0.451, 0.613) | 14 | 84.5 |
| Linoleic acid | N/A | 3 | 88.6 | N/A | 2 | 0 | 0.684 (0.594, 0.851) | 4 | 69 | 0.589 (0.534, 0.638) | 2 | 0 | 0.469 (0.369, 0.564) | 4 | 69 |
| Linolenic acid | N/A | 3 | 91.8 | N/A | 1 | N/A | 0.681 (0.468, 0.819) | N/A | N/A | 0.521 (0.451, 0.585) | 1 | N/A | N/A | N/A | N/A |
| EPA | N/A | N/A | N/A | N/A | N/A | N/A | N/A | N/A | N/A | N/A | N/A | N/A | N/A | N/A | N/A |
| DHA | N/A | N/A | N/A | N/A | N/A | N/A | N/A | N/A | N/A | N/A | N/A | N/A | N/A | N/A | N/A |
| Trans-fat | N/A | N/A | N/A | N/A | N/A | N/A | N/A | N/A | N/A | N/A | N/A | N/A | N/A | N/A | N/A |
| Cholesterol | N/A | 16 | 86.5 | N/A | 8 | 74.3 | 0.568 (0.476, 0.648) | 13 | 65.7 | 0.648 (0.565, 0.719) | 8 | 74.3 | 0.477 (0.414, 0.536) | 13 | 65.7 |
| Lipid | N/A | N/A | N/A | N/A | 3 | 81.4 | N/A | 1 | N/A | 0.639 (0.379, 0.858) | 3 | 81.4 | 0.326 (0.139, 0.491) | 1 | N/A |
| Carbohydrate | 0.575 (0.464, 0.668) | 27 | 86.3 | N/A | 17 | 78.8 | 0.567 (0.499, 0.629) | 14 | 67.8 | 0.676 (0.619, 0.725) | 17 | 78.8 | 0.527 (0.439, 0.561) | 14 | 67.8 |
| Sucrose | N/A | N/A | N/A | N/A | N/A | N/A | N/A | N/A | N/A | N/A | N/A | N/A | N/A | N/A | N/A |
| Sugar | N/A | N/A | N/A | 0.296 (0.115, 0.462) | 4 | 0 | N/A | N/A | N/A | 0.715 (0.686, 0.742) | 4 | 0 | N/A | N/A | N/A |
| Starch | N/A | N/A | N/A | N/A | N/A | N/A | N/A | N/A | N/A | N/A | N/A | N/A | N/A | N/A | N/A |
| Fiber | 0.576 (0.445, 0.683) | 17 | 86.4 | 0.296 (0.115, 0.462) | 18 | 67.1 | 0.628 (0.548, 0.697) | 15 | 82.2 | 0.666 (0.616, 0.718) | 18 | 67.1 | 0.585 (0.512, 0.659) | 15 | 82.2 |
| Soluble fiber | N/A | 9 | 66.2 | N/A | 1 | N/A | 0.555 (0.471, 0.629) | N/A | N/A | 0.869 (0.756, 0.932) | 1 | N/A | N/A | N/A | N/A |
| Insoluble fiber | N/A | 9 | 72.2 | N/A | 1 | N/A | 0.565 (0.473, 0.644) | 2 | 0 | 0.899 (0.653, 0.933) | 1 | N/A | 0.667 (0.611, 0.724) | 2 | 0 |
| Alcohol | 0.692 (0.555, 0.788) | 8 | 88.5 | N/A | 13 | 93.8 | 0.763 (0.667, 0.833) | 5 | 76 | 0.896 (0.731, 0.866) | 13 | 93.8 | 0.813 (0.751, 0.861) | 5 | 76 |
| Vitamin A | 0.713 (0.594, 0.822) | 9 | 92.9 | 0.172 (-0.29, 0.353) | 3 | 0 | 0.535 (0.331, 0.644) | 8 | 64.1 | 0.689 (0.659, 0.724) | 3 | 0 | 0.548 (0.464, 0.622) | 8 | 64.1 |
| Retinol | 0.438 (0.234, 0.591) | 21 | 85.7 | 0.191 (-0.99, 0.374) | 8 | 36.7 | 0.496 (0.414, 0.579) | 7 | 79.6 | 0.613 (0.565, 0.656) | 8 | 36.7 | 0.472 (0.357, 0.573) | 7 | 79.6 |
| Carotene | 0.652 (0.514, 0.757) | 24 | 86.2 | 0.326 (0.143, 0.488) | 17 | 60.6 | 0.573 (0.498, 0.634) | 14 | 67.8 | 0.673 (0.628, 0.714) | 17 | 60.6 | 0.562 (0.521, 0.616) | 14 | 67.8 |
| β-Carotene | N/A | 3 | 0 | N/A | 6 | 72.6 | 0.513 (0.444, 0.675) | 6 | 76.2 | 0.661 (0.559, 0.743) | 6 | 72.6 | 0.471 (0.342, 0.583) | 6 | 76.2 |
| Vitamin E | 0.631 (0.524, 0.718) | 7 | 93.2 | N/A | 14 | 80 | 0.618 (0.391, 0.752) | 7 | 75.7 | 0.575 (0.499, 0.642) | 14 | 80 | 0.429 (0.322, 0.525) | 7 | 75.7 |
| Vitamin K | N/A | 3 | 0 | N/A | 2 | 76.2 | 0.648 (0.568, 0.721) | N/A | N/A | 0.692 (0.146, 0.921) | 2 | 76.2 | N/A | N/A | N/A |
| Thiamin | 0.581 (0.426, 0.739) | 22 | 73.7 | 0.212 (0.899, 0.379) | 4 | 81.6 | 0.511 (0.452, 0.563) | 11 | 76.3 | 0.687 (0.579, 0.771) | 4 | 81.6 | 0.493 (0.396, 0.579) | 11 | 76.3 |
| Riboflavin | N/A | 22 | 79.7 | 0.212 (0.899, 0.379) | 5 | 86.6 | 0.555 (0.494, 0.611) | 7 | 47.3 | 0.725 (0.639, 0.793) | 5 | 86.6 | 0.568 (0.492, 0.636) | 7 | 47.3 |
| Niacin | N/A | 23 | 89.5 | 0.346 (0.163, 0.553) | 2 | 84.3 | 0.497 (0.478, 0.577) | 8 | 34.4 | 0.675 (0.453, 0.836) | 2 | 84.3 | 0.548 (0.482, 0.681) | 8 | 34.4 |
| Vitamin B6 | N/A | 9 | 59.5 | N/A | 3 | 64.4 | 0.564 (0.421, 0.581) | 7 | 74.1 | 0.722 (0.619, 0.849) | 3 | 64.4 | 0.521 (0.398, 0.625) | 7 | 74.1 |
| Folate | N/A | 11 | 88.4 | 0.268 (0.798, 0.438) | 6 | 14.7 | 0.637 (0.475, 0.769) | 8 | 43.5 | 0.687 (0.643, 0.727) | 6 | 14.7 | 0.586 (0.523, 0.644) | 8 | 43.5 |
| Vitamin B12 | N/A | 7 | 37.6 | N/A | 6 | 85.5 | 0.473 (0.396, 0.544) | 8 | 68.6 | 0.747 (0.633, 0.829) | 6 | 85.5 | 0.553 (0.469, 0.591) | 8 | 68.6 |
| Carotene | N/A | 15 | 88 | N/A | 2 | 89.1 | 0.499 (0.393, 0.592) | 4 | 83.1 | 0.661 (0.541, 0.753) | 2 | 89.1 | 0.453 (0.322, 0.567) | 4 | 83.1 |
| β-Carotene | 0.561 (0.391, 0.692) | 12 | 30.4 | 0.433 (0.262, 0.577) | 10 | 64.4 | 0.521 (0.474, 0.564) | 4 | 63.1 | 0.615 (0.535, 0.684) | 10 | 64.4 | 0.539 (0.399, 0.654) | 4 | 63.1 |
| Se | 0.581 (0.426, 0.739) | 7 | 90 | N/A | 1 | N/A | 0.551 (0.381, 0.685) | 2 | 0 | 0.827 (0.723, 0.895) | 1 | N/A | 0.449 (0.333, 0.551) | 2 | 0 |
| Mg | 0.571 (0.413, 0.696) | 8 | 83.5 | 0.473 (0.386, 0.614) | 4 | 3.6 | 0.695 (0.595, 0.774) | 5 | 89.8 | 0.696 (0.615, 0.762) | 4 | 3.6 | 0.499 (0.288, 0.664) | 5 | 89.8 |
| Ca | 0.616 (0.513, 0.718) | 24 | 86.8 | 0.482 (0.319, 0.617) | 12 | 62.3 | 0.654 (0.529, 0.662) | 16 | 81.4 | 0.636 (0.577, 0.689) | 12 | 62.3 | 0.528 (0.452, 0.596) | 16 | 81.4 |
| Fe | 0.631 (0.531, 0.714) | 22 | 83.9 | 0.277 (0.897, 0.446) | 12 | 76.2 | 0.537 (0.466, 0.618) | 10 | 72.2 | 0.676 (0.681, 0.734) | 12 | 76.2 | 0.528 (0.442, 0.652) | 10 | 72.2 |
| I | N/A | N/A | N/A | N/A | N/A | N/A | N/A | N/A | 0 | N/A | N/A | N/A | N/A | N/A | N/A |
| Zn | N/A | 9 | 87.9 | 0.384 (0.269, 0.537) | 2 | 17.7 | 0.651 (0.528, 0.747) | 6 | 54.9 | 0.723 (0.633, 0.794) | 2 | 17.7 | 0.482 (0.388, 0.572) | 6 | 54.9 |
| Cu | N/A | 5 | 87.3 | N/A | 1 | N/A | 0.739 (0.568, 0.819) | 1 | N/A | 0.827 (0.723, 0.895) | 1 | N/A | N/A | 1 | N/A |
| K | N/A | 21 | 80.1 | N/A | 6 | 0 | 0.596 (0.535, 0.652) | 7 | 27.7 | 0.679 (0.636, 0.717) | 6 | 0 | 0.598 (0.531, 0.644) | 7 | 27.7 |
| P | N/A | 1 | N/A | N/A | 21 | 79.7 | 0.574 (0.513, 0.629) | 3 | 37.5 | 0.696 (0.564, 0.821) | 21 | 79.7 | 0.563 (0.374, 0.774) | 3 | 37.5 |
| N/A | N/A | N/A | N/A | N/A | 21 | 88.7 | N/A | 5 | 77.9 | 0.545 (0.452, 0.618) | 21 | 88.7 | 0.648 (0.558, 0.723) | 5 | 77.9 |
| Mn | N/A | N/A | N/A | N/A | N/A | N/A | N/A | N/A | N/A | N/A | N/A | N/A | N/A | N/A | N/A |

*CI, confidence interval; N/A: not available
