# Supplementary material for: A meta-analysis of the reproducibility of food frequency questionnaires in nutritional epidemiological studies
Source: Int J Behav Nutr Phys Act. 2021 Jan 11;18:12. doi: 10.1186/s12966-020-01078-4 (PMC7802360; doi:10.1186/s12966-020-01078-4)
Supplement: Supplementary file 11 — Additional file 11 Supplemental Table 10. Pooled intraclass correlation coefficients for energy and nutrients stratified by sample size. [file 12966_2020_1078_MOESM11_ESM.docx]

**Supplemental Table 10. Pooled intraclass correlation coefficients for energy and nutrients stratified by sample size ***

| Nutrient | ≤ 112 | | | | | | ＞ 112 | | | | | |
| --- | --- | --- | --- | --- | --- | --- | --- | --- | --- | --- | --- | --- |
|  | Crude | | | Energy-adjusted | | | Crude | | | Energy-adjusted | | |
|  | ICC (95% CI) | N | *I^2^* | ICC (95% CI) | N | *I^2^* | ICC (95% CI) | N | *I^2^* | ICC (95% CI) | N | *I^2^* |
| Energy | 0.723 (0.669, 0.768) | 33 | 82 | N/A | N/A | N/A | 0.690 (0.592, 0.768) | 28 | 98.1 | N/A | N/A | N/A |
| Protein | 0.678 (0.624, 0.725) | 32 | 79.4 | 0.615 (0.550, 0.672) | 15 | 63.1 | 0.621 (0.569, 0.668) | 31 | 92 | 0.581 (0.483, 0.664) | 10 | 89.3 |
| Fat | 0.687 (0.627, 0.739) | 28 | 80.7 | 0.572 (0.456, 0.669) | 12 | 82.1 | 0.601 (0.533, 0.661) | 27 | 94.4 | 0.553 (0.424, 0.659) | 7 | 91.4 |
| Plant fat | 0.519 (0.273, 0.702) | 1 | N/A | N/A | N/A | N/A | 0.579 (0.449, 0.684) | 4 | 68.1 | N/A | N/A | N/A |
| Animal fat | N/A | N/A | N/A | N/A | N/A | N/A | N/A | N/A | N/A | N/A | N/A | N/A |
| MUFA | 0.652 (0.600, 0.698) | 23 | 63.9 | 0.611 (0.528, 0.683) | 14 | 71.2 | 0.629 (0.573, 0.679) | 18 | 88.3 | 0.675 (0.526, 0.784) | 4 | 94.2 |
| PUFA | 0.638 (0.581, 0.688) | 23 | 67.5 | 0.531 (0.441, 0.611) | 14 | 68.2 | 0.639 (0.532, 0.726) | 22 | 97.1 | 0.672 (0.529, 0.778) | 4 | 93.7 |
| n-3 PUFA | N/A | N/A | N/A | N/A | N/A | N/A | N/A | N/A | N/A | N/A | N/A | N/A |
| n-6 PUFA | N/A | N/A | N/A | N/A | N/A | N/A | N/A | N/A | N/A | N/A | N/A | N/A |
| SFA | 0.689 (0.632, 0.739) | 27 | 78.5 | 0.610 (0.520, 0.687) | 15 | 77.7 | 0.681 (0.550, 0.779) | 22 | 98.3 | 0.717 (0.581, 0.814) | 4 | 94.3 |
| Linoleic acid | 0.742 (0.649, 0.813) | 2 | 0 | 0.679 (0.533, 0.786) | 1 | N/A | 0.617 (0.332, 0.799) | 3 | 96.4 | 0.689 (0.551, 0.790) | 2 | 85.2 |
| Linolenic acid | 0.742 (0.649, 0.813) | 2 | 0 | N/A | N/A | N/A | 0.573 (0.254, 0.779) | 2 | 95.6 | N/A | N/A | N/A |
| EPA | N/A | N/A | N/A | N/A | N/A | N/A | N/A | N/A | N/A | N/A | N/A | N/A |
| DHA | N/A | N/A | N/A | N/A | N/A | N/A | N/A | N/A | N/A | N/A | N/A | N/A |
| Trans-fat | 0.628 (0.199, 0.855) | 2 | 88.4 | N/A | N/A | N/A | 0.590 (0.401, 0.731) | 2 | 82.7 | N/A | N/A | N/A |
| Cholesterol | 0.678 (0.627, 0.724) | 24 | 68.7 | 0.619 (0.552, 0.679) | 16 | 64.6 | 0.634 (0.559, 0.699) | 24 | 94.5 | 0.610 (0.505, 0.697) | 9 | 91 |
| Lipid | 0.709 (0.596, 0.795) | 1 | N/A | 0.499 (0.336, 0.633) | 1 | N/A | 0.696 (0.320, 0.882) | 2 | 95.1 | 0.707 (0.410, 0.869) | 3 | 93.2 |
| Carbohydrate | 0.700 (0.642, 0.750) | 31 | 82.6 | 0.618 (0.537, 0.687) | 13 | 71.8 | 0.657 (0.552, 0.742) | 31 | 98.3 | 0.657 (0.538, 0.751) | 10 | 94.5 |
| Sucrose | N/A | N/A | N/A | N/A | N/A | N/A | 0.633 (0.533, 0.715) | 4 | 73.1 | N/A | N/A | N/A |
| Sugar | 0.735 (0.632, 0.812) | 6 | 70.7 | N/A | N/A | N/A | 0.635 (0.373, 0.803) | 2 | 94.8 | N/A | N/A | N/A |
| Starch | 0.601 (0.393, 0.750) | 2 | 67.9 | N/A | N/A | N/A | 0.310 (0.174, 0.434) | 1 | N/A | N/A | N/A | N/A |
| Fiber | 0.694 (0.634, 0.745) | 27 | 82.1 | 0.693 (0.615, 0.758) | 12 | 78.1 | 0.672 (0.607, 0.729) | 27 | 94.8 | 0.641 (0.499, 0.749) | 9 | 95.4 |
| Soluble fiber | N/A | N/A | N/A | N/A | N/A | N/A | N/A | N/A | N/A | N/A | N/A | N/A |
| Insoluble fiber | N/A | N/A | N/A | N/A | N/A | N/A | N/A | N/A | N/A | N/A | N/A | N/A |
| Alcohol | 0.818 (0.771, 0.856) | 16 | 74.3 | 0.770 (0.727, 0.807) | 7 | 2.3 | 0.764 (0.598, 0.867) | 6 | 96.7 | 0.884 (0.811, 0.930) | 2 | 92.4 |
| Vitamin A | 0.632 (0.555, 0.699) | 14 | 76.8 | 0.625 (0.482, 0.735) | 7 | 86.4 | 0.613 (0.483, 0.716) | 13 | 97.5 | 0.558 (0.276, 0.751) | 5 | 95.6 |
| Retinol | 0.595 (0.509, 0.669) | 10 | 57.3 | 0.577 (0.446, 0.685) | 7 | 66.5 | 0.584 (0.463, 0.684) | 8 | 91.6 | 0.420 (0.263, 0.555) | 2 | 67.1 |
| Carotene | 0.529 (0.363, 0.663) | 1 | N/A | 0.480 (0.304, 0.624) | 1 | N/A | 0.642 (0.503, 0.749) | 8 | 97.4 | 0.521 (0.286, 0.697) | 4 | 89.7 |
| β-Carotene | 0.654 (0.570, 0.724) | 11 | 66.6 | 0.596 (0.335, 0.773) | 5 | 85.2 | 0.697 (0.638, 0.747) | 8 | 77.9 | 0.660 (0.613, 0.701) | 1 | N/A |
| Vitamin C | 0.661 (0.590, 0.722) | 24 | 83.8 | 0.645 (0.546, 0.727) | 14 | 84.4 | 0.669 (0.568, 0.750) | 23 | 97.9 | 0.617 (0.383, 0.777) | 8 | 97.8 |
| Vitamin D | 0.594 (0.515, 0.664) | 4 | 0 | 0.469 (0.311, 0.603) | 1 | N/A | 0.703 (0.553, 0.809) | 12 | 98.8 | 0.709 (0.404, 0.872) | 3 | 98.5 |
| Vitamin E | 0.652 (0.561, 0.727) | 15 | 84.7 | 0.559 (0.406, 0.681) | 7 | 84.8 | 0.672 (0.544, 0.770) | 19 | 98.5 | 0.641 (0.467, 0.767) | 8 | 96.4 |
| Vitamin K | 0.732 (0.636, 0.805) | 2 | 0 | N/A | N/A | N/A | 0.574 (0.197, 0.803) | 2 | 99 | N/A | N/A | N/A |
| Thiamin | 0.652 (0.560, 0.728) | 12 | 81.7 | 0.608 (0.446, 0.731) | 6 | 86.6 | 0.619 (0.566, 0.667) | 19 | 89.7 | 0.605 (0.428, 0.738) | 6 | 95.9 |
| Riboflavin | 0.646 (0.597, 0.691) | 11 | 26.2 | 0.549 (0.420, 0.657) | 4 | 58.1 | 0.676 (0.609, 0.733) | 17 | 94.7 | 0.660 (0.480, 0.786) | 6 | 96.7 |
| Niacin | 0.698 (0.610, 0.768) | 11 | 80.4 | 0.581 (0.426, 0.703) | 4 | 76.7 | 0.639 (0.558, 0.707) | 11 | 92 | 0.619 (0.472, 0.733) | 6 | 93.9 |
| Vitamin B6 | 0.696 (0.564, 0.792) | 5 | 75.2 | 0.526 (0.400, 0.633) | 2 | 18 | 0.737 (0.447, 0.887) | 8 | 99 | 0.797 (0.581, 0.908) | 3 | 97.8 |
| Folate | 0.660 (0.535, 0.757) | 11 | 87.9 | 0.598 (0.443, 0.719) | 5 | 81.1 | 0.624 (0.557, 0.683) | 14 | 92.2 | 0.599 (0.547, 0.647) | 1 | N/A |
| Vitamin B12 | 0.685 (0.484, 0.818) | 4 | 87.1 | 0.656 (0.492, 0.774) | 3 | 76.1 | 0.675 (0.445, 0.821) | 9 | 98.4 | 0.702 (0.411, 0.863) | 4 | 98.3 |
| Se | 0.661 (0.555, 0.746) | 7 | 76.6 | 0.586 (0.429, 0.709) | 4 | 78.7 | 0.676 (0.632, 0.715) | 4 | 36.1 | N/A | N/A | N/A |
| Mg | 0.732 (0.657, 0.793) | 12 | 78.1 | 0.656 (0.567, 0.729) | 4 | 49.1 | 0.577 (0.479, 0.661) | 7 | 90.8 | 0.536 (0.141, 0.784) | 2 | 97 |
| Ca | 0.676 (0.619, 0.727) | 24 | 77.6 | 0.687 (0.591, 0.764) | 14 | 85.6 | 0.600 (0.531, 0.661) | 28 | 94.8 | 0.571 (0.443, 0.676) | 9 | 93.5 |
| Fe | 0.663 (0.600, 0.718) | 21 | 76.9 | 0.605 (0.535, 0.667) | 15 | 66.5 | 0.615 (0.519, 0.696) | 18 | 96.8 | 0.435 (0.304, 0.550) | 4 | 86.1 |
| I | N/A | N/A | N/A | N/A | N/A | N/A | N/A | N/A | N/A | N/A | N/A | N/A |
| Zn | 0.621 (0.551, 0.682) | 16 | 71.1 | 0.592 (0.501, 0.670) | 10 | 66.5 | 0.568 (0.524, 0.610) | 10 | 65.4 | 0.499 (0.244, 0.688) | 2 | 91.9 |
| Cu | 0.682 (0.587, 0.758) | 2 | 0 | N/A | N/A | N/A | 0.653 (0.611, 0.692) | 2 | 0 | N/A | N/A | N/A |
| K | 0.743 (0.677, 0.798) | 12 | 75.8 | 0.682 (0.546, 0.783) | 5 | 83.1 | 0.597 (0.469, 0.701) | 13 | 97.4 | 0.516 (0.060, 0.793) | 2 | 98.3 |
| P | 0.693 (0.649, 0.731) | 13 | 21.1 | 0.684 (0.624, 0.737) | 6 | 0 | 0.489 (0.344, 0.611) | 10 | 95 | 0.552 (0.334, 0.714) | 3 | 94 |
| N/A | 0.658 (0.581, 0.724) | 13 | 74.5 | 0.666 (0.493, 0.789) | 5 | 88.8 | 0.650 (0.369, 0.822) | 12 | 99.2 | 0.674 (0.218, 0.888) | 3 | 99 |
| Mn | N/A | N/A | N/A | N/A | N/A | N/A | N/A | N/A | N/A | N/A | N/A | N/A |

* CI, confidence interval; N/A: not available
