# Supplementary material for: A meta-analysis of the reproducibility of food frequency questionnaires in nutritional epidemiological studies
Source: Int J Behav Nutr Phys Act. 2021 Jan 11;18:12. doi: 10.1186/s12966-020-01078-4 (PMC7802360; doi:10.1186/s12966-020-01078-4)
Supplement: Supplementary file 12 — Additional file 12 Supplemental Table 11. Pooled spearman correlation coefficients for energy and nutrients stratified by sample size. [file 12966_2020_1078_MOESM12_ESM.docx]

**Supplemental Table 11. Pooled spearman correlation coefficients for energy and nutrients stratified by sample size**

| Nutrient | ≤ 112 | | | | | | ＞ 112 | | | | | |
| --- | --- | --- | --- | --- | --- | --- | --- | --- | --- | --- | --- | --- |
|  | Crude | | | Energy-adjusted | | | Crude | | | Energy-adjusted | | |
|  | SCC (95% CI) | N | *I^2^* | SCC (95% CI) | N | *I^2^* | SCC (95% CI) | N | *I^2^* | SCC (95% CI) | N | *I^2^* |
| Energy | 0.658 (0.604, 0.707) | 47 | 85 | N/A | N/A | N/A | 0.638 (0.609, 0.665) | 61 | 84.5 | N/A | N/A | N/A |
| Protein | 0.601 (0.543, 0.654) | 47 | 83.9 | 0.549 (0.508, 0.586) | 40 | 78.3 | 0.605 (0.579, 0.631) | 61 | 79.5 | 0.572 (0.488, 0.645) | 24 | 83.4 |
| Fat | 0.626 (0.573, 0.672) | 46 | 80.7 | 0.543 (0.499, 0.583) | 35 | 79.2 | 0.616 (0.590, 0.640) | 60 | 77.6 | 0.583 (0.492, 0.661) | 21 | 84.2 |
| Plant fat | 0.675 (0.579, 0.752) | 3 | 0 | N/A | N/A | N/A | 0.482 (0.434, 0.527) | 3 | 0 | N/A | N/A | N/A |
| Animal fat | 0.690 (0.530, 0.802) | 1 | N/A | N/A | N/A | N/A | 0.695 (0.657, 0.730) | 3 | 23.2 | N/A | N/A | N/A |
| MUFA | 0.650 (0.603, 0.692) | 33 | 82 | 0.535 (0.466, 0.597) | 17 | 80.5 | 0.583 (0.546, 0.616) | 28 | 98.1 | 0.572 (0.470, 0.659) | 15 | 83.2 |
| PUFA | 0.624 (0.556, 0.683) | 23 | 79.8 | 0.499 (0.434, 0.559) | 17 | 77.8 | 0.580 (0.549, 0.609) | 34 | 66 | 0.554 (0.450, 0.644) | 14 | 82.4 |
| n-3 PUFA | 0.699 (0.544, 0.809) | 1 | N/A | 0.472 (0.398, 0.540) | 4 | 44.6 | 0.613 (0.567, 0.656) | 5 | 60.4 | 0.450 (0.223, 0.630) | 1 | N/A |
| n-6 PUFA | 0.570 (0.371, 0.718) | 1 | N/A | 0.434 (0.341, 0.520) | 4 | 62.1 | 0.596 (0.562, 0.627) | 5 | 26 | 0.499 (0.283, 0.667) | 1 | N/A |
| SFA | 0.648 (0.591, 0.699) | 26 | 77.4 | 0.537 (0.480, 0.588) | 21 | 81 | 0.612 (0.581, 0.641) | 39 | 76.1 | 0.611 (0.523, 0.687) | 16 | 81.9 |
| Linoleic acid | 0.731 (0.568, 0.838) | 2 | 73.3 | 0.536 (0.458, 0.605) | 6 | 77.5 | 0.586 (0.526, 0.642) | 7 | 76.9 | 0.658 (0.349, 0.838) | 3 | 92.2 |
| Linolenic acid | 0.809 (0.731, 0.867) | 1 | N/A | 0.555 (0.481, 0.621) | 3 | 48 | 0.621 (0.580, 0.660) | 2 | 0 | 0.829 (0.759, 0.881) | 1 | N/A |
| EPA | 0.785 (0.579, 0.896) | 3 | 87 | N/A | N/A | N/A | N/A | N/A | N/A | N/A | N/A | N/A |
| DHA | 0.749 (0.616, 0.840) | 3 | 67.4 | N/A | N/A | N/A | N/A | N/A | N/A | N/A | N/A | N/A |
| Trans-fat | 0.620 (0.505, 0.713) | 4 | 48.6 | N/A | N/A | N/A | 0.619 (-0.00, 0.896) | 2 | 98 | N/A | N/A | N/A |
| Cholesterol | 0.652 (0.584, 0.711) | 24 | 80.9 | 0.535 (0.486, 0.581) | 24 | 76.6 | 0.595 (0.562, 0.626) | 42 | 78.1 | 0.600 (0.467, 0.706) | 13 | 88.2 |
| Lipid | 0.516 (0.379, 0.632) | 2 | 0 | 0.526 (0.329, 0.679) | 2 | 53.4 | 0.560 (0.496, 0.617) | 4 | 0 | 0.626 (-0.06, 0.911) | 2 | 93.8 |
| Carbohydrate | 0.648 (0.590, 0.698) | 42 | 84.7 | 0.592 (0.548, 0.633) | 40 | 84.3 | 0.623 (0.590, 0.654) | 61 | 87.6 | 0.571 (0.483, 0.647) | 20 | 82.7 |
| Sucrose | 0.611 (0.131, 0.858) | 2 | 90.3 | N/A | N/A | N/A | 0.725 (0.676, 0.768) | 5 | 41.9 | N/A | N/A | N/A |
| Sugar | 0.659 (0.443, 0.802) | 5 | 88 | 0.715 (0.686, 0.742) | 4 | 0 | 0.703 (0.653, 0.746) | 6 | 62.6 | 0.296 (0.110, 0.462) | 1 | N/A |
| starch | N/A | N/A | N/A | N/A | N/A | N/A | 0.641 (0.604, 0.675) | 4 | 0 | N/A | N/A | N/A |
| Fiber | 0.621 (0.564, 0.673) | 43 | 81.7 | 0.617 (0.564, 0.666) | 29 | 87.5 | 0.647 (0.611, 0.679) | 46 | 86.4 | 0.627 (0.564, 0.683) | 24 | 73.4 |
| Soluble fiber | 0.710 (0.492, 0.845) | 4 | 87.4 | 0.546 (0.455, 0.626) | 8 | 69 | 0.648 (0.592, 0.697) | 10 | 75.8 | 0.775 (0.439, 0.921) | 2 | 85.3 |
| Insoluble fiber | 0.693 (0.486, 0.826) | 4 | 85.3 | 0.583 (0.501, 0.654) | 10 | 74.9 | 0.634 (0.592, 0.672) | 8 | 0 | 0.727 (0.510, 0.857) | 2 | 64.4 |
| Alcohol | 0.841 (0.785, 0.883) | 14 | 85.8 | 0.804 (0.750, 0.848) | 20 | 92.7 | 0.855 (0.818, 0.884) | 33 | 94.8 | 0.754 (0.682, 0.812) | 7 | 68.2 |
| Vitamin A | 0.647 (0.564, 0.717) | 23 | 86.5 | 0.517 (0.402, 0.616) | 10 | 91.3 | 0.577 (0.527, 0.622) | 19 | 87 | 0.586 (0.444, 0.698) | 12 | 89 |
| Retinol | 0.558 (0.439, 0.658) | 14 | 85.6 | 0.496 (0.440, 0.548) | 27 | 82.1 | 0.577 (0.541, 0.611) | 35 | 78.1 | 0.555 (0.404, 0.677) | 11 | 88.6 |
| Carotene | 0.606 (0.542, 0.662) | 37 | 82.4 | 0.601 (0.552, 0.646) | 37 | 86.7 | 0.632 (0.599, 0.663) | 55 | 86.9 | 0.584 (0.508, 0.650) | 20 | 73.5 |
| β-Carotene | 0.611 (0.513, 0.694) | 16 | 81.1 | 0.517 (0.391, 0.624) | 8 | 88.2 | 0.628 (0.577, 0.675) | 14 | 85.5 | 0.616 (0.498, 0.712) | 7 | 61.6 |
| Vitamin E | 0.611 (0.507, 0.699) | 20 | 88.5 | 0.521 (0.445, 0.589) | 18 | 86.4 | 0.633 (0.583, 0.680) | 32 | 92.8 | 0.605 (0.474, 0.709) | 12 | 87.6 |
| Vitamin K | 0.568 (0.355, 0.725) | 4 | 62.6 | 0.625 (0.515, 0.714) | 2 | 0 | 0.651 (0.611, 0.687) | 3 | 8.6 | 0.697 (0.471, 0.836) | 3 | 52.5 |
| Thiamin | 0.615 (0.555, 0.668) | 21 | 69.4 | 0.532 (0.483, 0.578) | 25 | 76.6 | 0.603 (0.571, 0.633) | 34 | 76.9 | 0.502 (0.379, 0.606) | 14 | 84.1 |
| Riboflavin | 0.647 (0.560, 0.720) | 19 | 84.8 | 0.576 (0.522, 0.624) | 24 | 83 | 0.637 (0.607, 0.665) | 35 | 78.8 | 0.592 (0.434, 0.715) | 11 | 89.7 |
| Niacin | 0.630 (0.527, 0.716) | 17 | 86.9 | 0.484 (0.403, 0.557) | 21 | 87.1 | 0.652 (0.553, 0.733) | 22 | 96.1 | 0.571 (0.458, 0.666) | 13 | 83.9 |
| Vitamin B6 | 0.629 (0.528, 0.713) | 15 | 83.5 | 0.532 (0.440, 0.613) | 11 | 79.9 | 0.587 (0.524, 0.644) | 12 | 68.7 | 0.590 (0.466, 0.691) | 8 | 74.2 |
| Folate | 0.631 (0.542, 0.706) | 23 | 87.3 | 0.603 (0.537, 0.662) | 14 | 78.8 | 0.604 (0.570, 0.636) | 26 | 71.5 | 0.607 (0.477, 0.711) | 12 | 86.4 |
| Vitamin B12 | 0.669 (0.583, 0.739) | 16 | 79.1 | 0.528 (0.410, 0.629) | 12 | 90.8 | 0.597 (0.512, 0.672) | 12 | 86.2 | 0.644 (0.511, 0.746) | 9 | 79.4 |
| Carotene | 0.678 (0.485, 0.808) | 4 | 87.4 | 0.483 (0.399, 0.558) | 18 | 89.6 | 0.592 (0.542, 0.638) | 21 | 89.2 | 0.663 (0.270, 0.866) | 3 | 94.3 |
| β-Carotene | 0.562 (0.492, 0.626) | 11 | 47.2 | 0.557 (0.508, 0.602) | 21 | 62.2 | 0.630 (0.583, 0.672) | 28 | 76.4 | 0.546 (0.466, 0.616) | 7 | 34.2 |
| Se | 0.700 (0.579, 0.791) | 8 | 87.7 | 0.471 (0.408, 0.529) | 6 | 8.9 | 0.595 (0.562, 0.626) | 7 | 8.4 | 0.673 (0.439, 0.821) | 5 | 92.7 |
| Mg | 0.695 (0.610, 0.764) | 21 | 87.9 | 0.590 (0.455, 0.699) | 8 | 89.8 | 0.604 (0.495, 0.695) | 9 | 89.5 | 0.659 (0.547, 0.748) | 11 | 82.2 |
| Ca | 0.617 (0.557, 0.671) | 41 | 83.6 | 0.551 (0.502, 0.597) | 33 | 84 | 0.622 (0.591, 0.651) | 48 | 82.6 | 0.645 (0.567, 0.710) | 22 | 82.3 |
| Fe | 0.575 (0.507, 0.637) | 36 | 83.4 | 0.554 (0.506, 0.599) | 30 | 80 | 0.627 (0.592, 0.660) | 41 | 85.2 | 0.603 (0.492, 0.694) | 17 | 86.8 |
| I | N/A | N/A | N/A | N/A | N/A | N/A | N/A | N/A | N/A | N/A | N/A | N/A |
| Zn | 0.625 (0.529, 0.705) | 15 | 84.6 | 0.655 (0.551, 0.739) | 7 | 83.5 | 0.616 (0.541, 0.682) | 11 | 84.5 | 0.555 (0.413, 0.671) | 11 | 87.1 |
| Cu | 0.796 (0.657, 0.883) | 4 | 87.4 | 0.638 (0.559, 0.705) | 4 | 45.3 | 0.619 (0.508, 0.710) | 2 | 0 | 0.849 (0.799, 0.887) | 2 | 0 |
| K | 0.666 (0.579, 0.739) | 18 | 83.3 | 0.592 (0.546, 0.635) | 22 | 71.2 | 0.623 (0.589, 0.655) | 31 | 77.3 | 0.640 (0.542, 0.721) | 12 | 75.8 |
| P | 0.632 (0.527, 0.717) | 17 | 83.1 | 0.613 (0.562, 0.660) | 26 | 83.9 | 0.613 (0.562, 0.660) | 26 | 83.9 | 0.635 (0.492, 0.743) | 17 | 83.1 |
| Na | 0.646 (0.560, 0.718) | 15 | 78.8 | 0.555 (0.492, 0.612) | 21 | 84 | 0.611 (0.561, 0.657) | 26 | 86.3 | 0.537 (0.326, 0.697) | 9 | 91.4 |
| Mn | 0.625 (0.544, 0.694) | 3 | 0 | N/A | N/A | N/A | 0.704 (0.612, 0.777) | 2 | 0 | N/A | N/A | N/A |
