# Supplementary material for: A meta-analysis of the reproducibility of food frequency questionnaires in nutritional epidemiological studies
Source: Int J Behav Nutr Phys Act. 2021 Jan 11;18:12. doi: 10.1186/s12966-020-01078-4 (PMC7802360; doi:10.1186/s12966-020-01078-4)
Supplement: Supplementary file 13 — Additional file 13 Supplemental Table 12. Pooled intraclass correlation coefficient for energy and nutrients stratified by time interval (6 months as cut-point). [file 12966_2020_1078_MOESM13_ESM.docx]

**Supplemental Table 12. Pooled intraclass correlation coefficient for energy and nutrients stratified by time interval (6 months as cut-point) ***

| Nutrient | ≤ 6 months | | | | | | > 6 months | | | | | |
| --- | --- | --- | --- | --- | --- | --- | --- | --- | --- | --- | --- | --- |
|  | Crude | | | Energy-adjusted | | | Crude | | | Energy-adjusted | | |
|  | ICC (95% CI) | N | *I^2^* | ICC (95% CI) | N | *I^2^* | ICC (95% CI) | N | *I^2^* | ICC (95% CI) | N | *I^2^* |
| Energy | 0.750 (0.701, 0.792) | 27 | 87.2 | N/A | N/A | N/A | 0.669 (0.566, 0.750) | 34 | 97.6 | N/A | N/A | N/A |
| Protein | 0.676 (0.627, 0.720) | 28 | 83.3 | 0.559 (0.451, 0.650) | 7 | 69.8 | 0.624 (0.570, 0.673) | 35 | 90.7 | 0.616 (0.551, 0.673) | 18 | 82.6 |
| Fat | 0.667 (0.606, 0.720) | 29 | 88.4 | 0.534 (0.391, 0.652) | 9 | 81.2 | 0.619 (0.556, 0.676) | 26 | 91.7 | 0.586 (0.484, 0.671) | 10 | 89.3 |
| MUFA | 0.637 (0.570, 0.696) | 18 | 78.5 | 0.581 (0.411, 0.712) | 6 | 80.1 | 0.646 (0.603, 0.687) | 23 | 80.2 | 0.650 (0.570, 0.719) | 12 | 84.6 |
| PUFA | 0.661 (0.584, 0.725) | 18 | 84.9 | 0.588 (0.426, 0.714) | 6 | 78.6 | 0.624 (0.527, 0.706) | 27 | 96.2 | 0.564 (0.458, 0.656) | 12 | 88 |
| SFA | 0.696 (0.633, 0.750) | 22 | 84.1 | 0.638 (0.472, 0.761) | 7 | 86.3 | 0.675 (0.554, 0.767) | 27 | 97.9 | 0.641 (0.551, 0.717) | 12 | 87.1 |
| Linoleic acid | 0.537 (-0.01, 0.839) | 2 | 93.3 | N/A | N/A | N/A | 0.732 (0.626, 0.812) | 3 | 81.5 | 0.685 (0.591, 0.760) | 3 | 71 |
| Linolenic acid | 0.608 (0.169, 0.846) | 2 | 91.1 | 0.630 (0.527, 0.714) | 2 | 47.8 | 0.694 (0.653, 0.730) | 2 | 0 | N/A | N/A | N/A |
| Trans-fat | 0.706 (0.596, 0.790) | 2 | 36.6 | N/A | N/A | N/A | 0.485 (0.390, 0.570) | 2 | 0 | 0.473 (0.360, 0.574) | 1 | N/A |
| Cholesterol | 0.666 (0.590, 0.731) | 21 | 87.4 | 0.594 (0.490, 0.681) | 8 | 65.7 | 0.650 (0.586, 0.705) | 27 | 92.1 | 0.626 (0.555, 0.688) | 17 | 85.2 |
| Lipid | N/A | N/A | N/A | N/A | N/A | N/A | 0.701 (0.459, 0.846) | 4 | 93.4 | 0.662 (0.370, 0.835) | 4 | 94.5 |
| Carbohydrate | 0.684 (0.617, 0.741) | 29 | 91 | 0.608 (0.486, 0.707) | 8 | 79 | 0.673 (0.574, 0.753) | 33 | 97.6 | 0.654 (0.562, 0.731) | 15 | 92.1 |
| Sucrose | 0.529 (0.418, 0.625) | 1 | N/A | N/A | N/A | N/A | 0.666 (0.575, 0.740) | 3 | 59.6 | 0.679 (0.607, 0.741) | 1 | N/A |
| Sugar | 0.715 (0.547, 0.828) | 5 | 85.7 | N/A | N/A | N/A | 0.700 (0.593, 0.783) | 3 | 71.4 | 0.779 (0.747, 0.808) | 1 | N/A |
| Starch | 0.518 (0.057, 0.797) | 2 | 92.3 | N/A | N/A | N/A | 0.510 (0.351, 0.640) | 1 | N/A | N/A | N/A | N/A |
| Fiber | 0.701 (0.637, 0.755) | 25 | 87.7 | 0.639 (0.488, 0.752) | 6 | 85 | 0.667 (0.604, 0.723) | 29 | 93.6 | 0.682 (0.591, 0.756) | 15 | 92.6 |
| Alcohol | 0.822 (0.749, 0.876) | 9 | 84.3 | 0.800 (0.707, 0.865) | 1 | N/A | 0.788 (0.705, 0.849) | 13 | 91.9 | 0.803 (0.717, 0.865) | 8 | 89.5 |
| Vitamin A | 0.634 (0.515, 0.729) | 14 | 94 | 0.604 (0.317, 0.790) | 4 | 92.7 | 0.613 (0.509, 0.700) | 13 | 94.2 | 0.595 (0.424, 0.724) | 8 | 93 |
| Retinol | 0.601 (0.432, 0.730) | 5 | 91.6 | 0.529 (0.300, 0.701) | 1 | N/A | 0.586 (0.508, 0.654) | 13 | 70.2 | 0.538 (0.409, 0.646) | 8 | 77.2 |
| Carotene | 0.588 (0.407, 0.724) | 2 | 88.3 | N/A | N/A | N/A | 0.644 (0.504, 0.750) | 7 | 95.5 | 0.512 (0.328, 0.658) | 5 | 86.2 |
| β-Carotene | 0.765 (0.651, 0.845) | 5 | 82.9 | N/A | N/A | N/A | 0.649 (0.603, 0.690) | 14 | 56.1 | 0.613 (0.456, 0.733) | 6 | 81.9 |
| Vitamin C | 0.679 (0.602, 0.744) | 21 | 92.2 | 0.649 (0.468, 0.777) | 7 | 91.2 | 0.653 (0.555, 0.732) | 26 | 96.7 | 0.627 (0.484, 0.738) | 15 | 95.9 |
| Vitamin D | 0.576 (0.529, 0.619) | 9 | 37.5 | 0.469 (0.311, 0.603) | 1 | N/A | 0.776 (0.586, 0.885) | 7 | 99.1 | 0.709 (0.404, 0.872) | 3 | 98.5 |
| Vitamin E | 0.667 (0.565, 0.748) | 14 | 93.5 | 0.523 (0.321, 0.679) | 4 | 83.5 | 0.661 (0.535, 0.759) | 20 | 97.8 | 0.632 (0.489, 0.742) | 11 | 95.4 |
| Vitamin K | 0.590 (0.141, 0.838) | 2 | 92.7 | N/A | N/A | N/A | 0.710 (0.672, 0.745) | 2 | 0 | N/A | N/A | N/A |
| Thiamin | 0.636 (0.567, 0.697) | 14 | 84.5 | 0.647 (0.419, 0.799) | 4 | 90.4 | 0.626 (0.568, 0.678) | 17 | 87.7 | 0.586 (0.439, 0.702) | 8 | 94.5 |
| Riboflavin | 0.635 (0.577, 0.686) | 12 | 73.3 | 0.390 (0.194, 0.555) | 1 | N/A | 0.687 (0.623, 0.741) | 16 | 91.9 | 0.639 (0.502, 0.746) | 9 | 94.8 |
| Niacin | 0.653 (0.566, 0.725) | 12 | 87.9 | 0.449 (0.329, 0.554) | 2 | 0 | 0.682 (0.616, 0.739) | 10 | 83.2 | 0.635 (0.521, 0.727) | 8 | 91.8 |
| Vitamin B6 | 0.616 (0.476, 0.725) | 5 | 74.2 | 0.459 (0.274, 0.611) | 1 | N/A | 0.769 (0.508, 0.901) | 8 | 99 | 0.756 (0.559, 0.872) | 4 | 97 |
| Folate | 0.616 (0.528, 0.691) | 11 | 86.7 | 0.433 (0.309, 0.542) | 2 | 0 | 0.651 (0.573, 0.718) | 14 | 92 | 0.658 (0.561, 0.738) | 4 | 69.9 |
| Vitamin B12 | 0.577 (0.455, 0.678) | 4 | 67.1 | 0.539 (0.370, 0.674) | 1 | N/A | 0.713 (0.495, 0.847) | 9 | 98.3 | 0.702 (0.501, 0.832) | 6 | 97.2 |
| Se | 0.660 (0.587, 0.723) | 8 | 75.6 | 0.576 (0.346, 0.741) | 3 | 85.3 | 0.662 (0.583, 0.730) | 3 | 37.5 | 0.619 (0.482, 0.727) | 1 | N/A |
| Mg | 0.703 (0.616, 0.774) | 12 | 89.1 | 0.649 (0.410, 0.805) | 2 | 82.2 | 0.631 (0.521, 0.721) | 7 | 87.9 | 0.601 (0.423, 0.735) | 4 | 91.3 |
| Ca | 0.653 (0.598, 0.703) | 20 | 82.9 | 0.648 (0.442, 0.789) | 6 | 91.6 | 0.622 (0.552, 0.683) | 32 | 94 | 0.641 (0.553, 0.714) | 17 | 91.2 |
| Fe | 0.643 (0.562, 0.711) | 15 | 89.3 | 0.607 (0.479, 0.710) | 7 | 80.4 | 0.638 (0.563, 0.701) | 24 | 93.5 | 0.535 (0.455, 0.607) | 12 | 76.6 |
| Zn | 0.583 (0.519, 0.642) | 10 | 72.5 | 0.444 (0.330, 0.546) | 2 | 0 | 0.603 (0.549, 0.653) | 16 | 68.3 | 0.598 (0.514, 0.671) | 10 | 72.4 |
| Cu | 0.676 (0.610, 0.732) | 3 | 0 | N/A | N/A | N/A | 0.649 (0.602, 0.692) | 1 | N/A | N/A | N/A | N/A |
| K | 0.722 (0.630, 0.793) | 12 | 91 | 0.633 (0.111, 0.881) | 2 | 95.2 | 0.623 (0.502, 0.719) | 13 | 96.2 | 0.639 (0.455, 0.771) | 5 | 94.5 |
| P | 0.623 (0.483, 0.731) | 8 | 91.3 | N/A | N/A | N/A | 0.595 (0.498, 0.677) | 15 | 87.3 | 0.635 (0.544, 0.711) | 9 | 80.7 |
| Na | 0.643 (0.580, 0.698) | 13 | 74.6 | 0.725 (0.466, 0.870) | 3 | 92.7 | 0.664 (0.318, 0.854) | 12 | 99.1 | 0.633 (0.322, 0.820) | 5 | 98.1 |

* CI, confidence interval; N/A: not available
