# Supplementary material for: A meta-analysis of the reproducibility of food frequency questionnaires in nutritional epidemiological studies
Source: Int J Behav Nutr Phys Act. 2021 Jan 11;18:12. doi: 10.1186/s12966-020-01078-4 (PMC7802360; doi:10.1186/s12966-020-01078-4)
Supplement: Supplementary file 14 — Additional file 14 Supplemental Table 13. Pooled spearman correlation coefficient for energy and nutrients stratified by time interval (6 months as cut-point). [file 12966_2020_1078_MOESM14_ESM.docx]

**Supplemental Table 13. Pooled spearman correlation coefficient for energy and nutrients stratified by time interval (6 months as cut-point) ***

| Nutrient | ≤ 6 months | | | | | | > 6 months | | | | | |
| --- | --- | --- | --- | --- | --- | --- | --- | --- | --- | --- | --- | --- |
|  | Crude | | | Energy-adjusted | | | Crude | | | Energy-adjusted | | |
|  | SCC (95% CI) | N | *I^2^* | SCC (95% CI) | N | *I^2^* | SCC (95% CI) | N | *I^2^* | SCC (95% CI) | N | *I^2^* |
| Energy | 0.691 (0.650, 0.727) | 49 | 86.9 | N/A | N/A | N/A | 0.616 (0.586, 0.645) | 59 | 79.6 | N/A | N/A | N/A |
| Protein | 0.641 (0.599, 0.679) | 49 | 84.6 | 0.600 (0.528, 0.664) | 25 | 86.9 | 0.586 (0.556, 0.615) | 59 | 75.8 | 0.532 (0.493, 0.567) | 39 | 69 |
| Fat | 0.665 (0.629, 0.698) | 50 | 81.5 | 0.599 (0.516, 0.672) | 21 | 87.9 | 0.586 (0.560, 0.612) | 56 | 68.5 | 0.530 (0.489, 0.569) | 35 | 71.3 |
| Plant fat | 0.605 (0.513, 0.683) | 5 | 44.7 | N/A | N/A | N/A | 0.464 (0.406, 0.518) | 3 | 0 | N/A | N/A | N/A |
| Animal fat | 0.723 (0.679, 0.762) | 4 | 0.7 | N/A | N/A | N/A | 0.670 (0.624, 0.711) | 2 | 0 | 0.374 (0.221, 0.510) | 1 | N/A |
| MUFA | 0.670 (0.627, 0.709) | 27 | 87.2 | 0.613 (0.499, 0.705) | 11 | 87.3 | 0.568 (0.534, 0.599) | 34 | 97.6 | 0.519 (0.462, 0.572) | 21 | 69.8 |
| PUFA | 0.653 (0.602, 0.699) | 24 | 77.2 | 0.602 (0.504, 0.684) | 11 | 82.5 | 0.562 (0.533, 0.589) | 35 | 54 | 0.476 (0.421, 0.529) | 20 | 66.3 |
| n-3 PUFA | 0.699 (0.544, 0.809) | 1 | N/A | 0.450 (0.223, 0.630) | 1 | N/A | 0.613 (0.567, 0.656) | 5 | 60.4 | 0.472 (0.398, 0.540) | 4 | 44.6 |
| n-6 PUFA | 0.570 (0.371, 0.718) | 1 | N/A | 0.499 (0.283, 0.667) | 1 | N/A | 0.596 (0.562, 0.627) | 5 | 26 | 0.434 (0.341, 0.520) | 4 | 62.1 |
| SFA | 0.680 (0.638, 0.718) | 26 | 75.1 | 0.618 (0.523, 0.697) | 13 | 86.5 | 0.592 (0.558, 0.624) | 41 | 75.1 | 0.542 (0.488, 0.591) | 24 | 76.8 |
| Linoleic acid | 0.649 (0.529, 0.744) | 5 | 83.4 | 0.594 (0.439, 0.715) | 6 | 90.1 | 0.601 (0.541, 0.654) | 6 | 68.7 | 0.546 (0.455, 0.625) | 3 | 73.1 |
| Linolenic acid | 0.695 (0.544, 0.802) | 3 | 82.9 | 0.681 (0.468, 0.819) | 3 | 91.8 | 0.619 (0.568, 0.665) | 2 | 0 | 0.521 (0.451, 0.585) | 1 | N/A |
| Trans-fat | 0.667 (0.532, 0.769) | 5 | 78.8 | 0.789 (0.619, 0.889) | 1 | N/A | 0.339 (0.210, 0.455) | 1 | N/A | 0.296 (0.166, 0.417) | 1 | N/A |
| Cholesterol | 0.668 (0.618, 0.712) | 26 | 78.5 | 0.679 (0.596, 0.747) | 12 | 84.3 | 0.580 (0.545, 0.613) | 42 | 76.6 | 0.492 (0.442, 0.541) | 25 | 72.3 |
| Lipid | 0.532 (0.458, 0.599) | 3 | 0 | 0.820 (0.669, 0.905) | 1 | N/A | 0.581 (0.491, 0.659) | 3 | 0 | 0.459 (0.276, 0.610) | 3 | 63.7 |
| Carbohydrate | 0.675 (0.630, 0.716) | 45 | 88.2 | 0.639 (0.570, 0.699) | 22 | 86.5 | 0.607 (0.572, 0.639) | 58 | 83.9 | 0.554 (0.509, 0.596) | 38 | 79.5 |
| Sucrose | 0.656 (0.452, 0.794) | 3 | 81.6 | 0.632 (0.513, 0.726) | 1 | N/A | 0.730 (0.667, 0.782) | 4 | 56.3 | N/A | N/A | N/A |
| Sugar | 0.690 (0.597, 0.764) | 9 | 83.2 | 0.608 (0.397, 0.757) | 4 | 90.7 | 0.685 (0.643, 0.723) | 2 | 0 | 0.723 (0.677, 0.764) | 1 | N/A |
| starch | 0.628 (0.497, 0.731) | 4 | 74.2 | 0.606 (0.553, 0.654) | 2 | 0 | 0.636 (0.587, 0.681) | 3 | 0 | N/A | N/A | N/A |
| Fiber | 0.675 (0.627, 0.717) | 40 | 84.9 | 0.663 (0.594, 0.723) | 21 | 86.3 | 0.610 (0.572, 0.646) | 49 | 83.5 | 0.593 (0.546, 0.636) | 32 | 78.6 |
| Soluble fiber | 0.710 (0.492, 0.845) | 4 | 87.4 | 0.775 (0.439, 0.921) | 2 | 85.3 | 0.648 (0.592, 0.697) | 10 | 75.8 | 0.546 (0.455, 0.626) | 8 | 69 |
| Insoluble fiber | 0.693 (0.486, 0.826) | 4 | 85.3 | 0.727 (0.510, 0.857) | 2 | 64.4 | 0.634 (0.592, 0.672) | 8 | 0 | 0.583 (0.501, 0.654) | 10 | 74.9 |
| Alcohol | 0.860 (0.822, 0.890) | 24 | 90.7 | 0.809 (0.733, 0.865) | 11 | 90.2 | 0.840 (0.792, 0.878) | 25 | 94.6 | 0.781 (0.721, 0.830) | 16 | 90.2 |
| Vitamin A | 0.673 (0.605, 0.731) | 23 | 91.1 | 0.650 (0.495, 0.765) | 8 | 92.3 | 0.544 (0.505, 0.580) | 19 | 60.6 | 0.495 (0.406, 0.573) | 14 | 83.4 |
| Retinol | 0.605 (0.544, 0.660) | 19 | 84.6 | 0.537 (0.411, 0.642) | 14 | 92 | 0.551 (0.510, 0.590) | 32 | 71 | 0.501 (0.453, 0.546) | 24 | 68 |
| Vitamin C | 0.649 (0.606, 0.687) | 42 | 84.3 | 0.637 (0.571, 0.695) | 22 | 83.7 | 0.602 (0.562, 0.639) | 52 | 85.8 | 0.570 (0.521, 0.615) | 35 | 81.2 |
| Vitamin D | 0.621 (0.560, 0.676) | 18 | 81.3 | 0.610 (0.546, 0.667) | 7 | 39.7 | 0.611 (0.537, 0.677) | 12 | 85 | 0.524 (0.392, 0.635) | 8 | 82 |
| Vitamin E | 0.633 (0.557, 0.700) | 25 | 92.8 | 0.611 (0.500, 0.701) | 13 | 90.1 | 0.611 (0.562, 0.655) | 29 | 85.9 | 0.507 (0.437, 0.571) | 17 | 77.8 |
| Vitamin K | 0.618 (0.479, 0.728) | 4 | 73.8 | 0.753 (0.572, 0.864) | 2 | 36.2 | 0.583 (0.471, 0.677) | 3 | 0 | 0.610 (0.503, 0.699) | 3 | 0 |
| Thiamin | 0.648 (0.599, 0.692) | 23 | 82.1 | 0.586 (0.501, 0.659) | 13 | 83 | 0.581 (0.549, 0.611) | 34 | 64.3 | 0.489 (0.435, 0.540) | 26 | 73.6 |
| Riboflavin | 0.673 (0.615, 0.724) | 22 | 88.3 | 0.650 (0.537, 0.740) | 11 | 90.9 | 0.618 (0.584, 0.648) | 34 | 71.6 | 0.548 (0.499, 0.594) | 24 | 73 |
| Niacin | 0.654 (0.545, 0.742) | 14 | 92.4 | 0.611 (0.499, 0.703) | 11 | 86.2 | 0.636 (0.537, 0.718) | 25 | 94.6 | 0.469 (0.394, 0.537) | 23 | 83.9 |
| Vitamin B6 | 0.646 (0.532, 0.737) | 12 | 86.2 | 0.665 (0.580, 0.735) | 6 | 53.2 | 0.583 (0.524, 0.636) | 15 | 64.2 | 0.501 (0.415, 0.577) | 13 | 75.7 |
| Folate | 0.641 (0.579, 0.695) | 24 | 87.1 | 0.676 (0.575, 0.756) | 10 | 86.1 | 0.589 (0.548, 0.628) | 25 | 69.7 | 0.557 (0.494, 0.615) | 16 | 68.2 |
| Vitamin B12 | 0.684 (0.601, 0.753) | 13 | 83.1 | 0.695 (0.569, 0.789) | 7 | 86 | 0.585 (0.519, 0.645) | 15 | 70.8 | 0.498 (0.433, 0.557) | 14 | 60.5 |
| Carotene | 0.682 (0.606, 0.746) | 8 | 89.8 | 0.601 (0.378, 0.759) | 5 | 96.3 | 0.565 (0.503, 0.622) | 19 | 88 | 0.478 (0.401, 0.547) | 16 | 82.1 |
| β-Carotene | 0.621 (0.559, 0.677) | 16 | 67.9 | 0.610 (0.536, 0.675) | 10 | 58.1 | 0.607 (0.555, 0.654) | 23 | 74.7 | 0.524 (0.479, 0.566) | 18 | 45.3 |
| Se | 0.702 (0.599, 0.782) | 8 | 89.8 | 0.719 (0.471, 0.862) | 4 | 92.9 | 0.583 (0.533, 0.630) | 7 | 9.7 | 0.466 (0.411, 0.519) | 7 | 0 |
| Mg | 0.698 (0.618, 0.763) | 20 | 90.8 | 0.664 (0.573, 0.738) | 11 | 81.2 | 0.606 (0.469, 0.714) | 10 | 88.4 | 0.577 (0.413, 0.705) | 8 | 89.3 |
| Ca | 0.661 (0.621, 0.697) | 41 | 82.9 | 0.642 (0.577, 0.698) | 21 | 83.4 | 0.588 (0.549, 0.624) | 48 | 82.3 | 0.551 (0.499, 0.601) | 34 | 82.8 |
| Fe | 0.632 (0.579, 0.679) | 37 | 87.9 | 0.637 (0.546, 0.713) | 17 | 88.9 | 0.599 (0.564, 0.633) | 40 | 76 | 0.534 (0.486, 0.578) | 30 | 73.2 |
| I | 0.828 (0.724, 0.894) | 2 | 19.8 | 0.744 (0.600, 0.841) | 1 | N/A | N/A | N/A | N/A | N/A | N/A | N/A |
| Zn | 0.638 (0.558, 0.706) | 15 | 88.3 | 0.644 (0.511, 0.746) | 10 | 90.5 | 0.601 (0.509, 0.680) | 11 | 79 | 0.526 (0.458, 0.588) | 8 | 32 |
| Cu | 0.796 (0.657, 0.883) | 4 | 87.4 | 0.742 (0.564, 0.854) | 4 | 92 | 0.619 (0.508, 0.710) | 2 | 0 | 0.695 (0.601, 0.770) | 2 | 0 |
| K | 0.694 (0.622, 0.755) | 19 | 89 | 0.673 (0.590, 0.741) | 11 | 76.1 | 0.608 (0.574, 0.639) | 32 | 67.6 | 0.579 (0.532, 0.621) | 23 | 68.3 |
| P | 0.716 (0.637, 0.781) | 12 | 84.8 | 0.661 (0.526, 0.764) | 8 | 86.8 | 0.581 (0.523, 0.633) | 31 | 81.6 | 0.547 (0.486, 0.603) | 22 | 77.7 |
| Na | 0.666 (0.608, 0.716) | 19 | 83.6 | 0.635 (0.532, 0.720) | 11 | 88.2 | 0.581 (0.532, 0.626) | 24 | 73.1 | 0.498 (0.434, 0.557) | 19 | 76 |
| Mn | 0.625 (0.544, 0.694) | 3 | 0 | 0.703 (0.542, 0.814) | 1 | N/A | 0.704 (0.612, 0.777) | 2 | N/A | 0.724 (0.638, 0.793) | 1 | N/A |

* CI, confidence interval; N/A: not available
