# Supplementary material for: A meta-analysis of the reproducibility of food frequency questionnaires in nutritional epidemiological studies
Source: Int J Behav Nutr Phys Act. 2021 Jan 11;18:12. doi: 10.1186/s12966-020-01078-4 (PMC7802360; doi:10.1186/s12966-020-01078-4)
Supplement: Supplementary file 15 — Additional file 15 Supplemental Table 14. Pooled intraclass correlation coefficient for energy and nutrients stratified by time interval (12 months as cut-point). [file 12966_2020_1078_MOESM15_ESM.docx]

**Supplemental Table 14.** **Pooled intraclass correlation coefficient for energy and nutrients stratified by time interval (12 months as cut-point) ***

| Nutrient | < 12 months | | | | | | ≥ 12 months | | | | | |
| --- | --- | --- | --- | --- | --- | --- | --- | --- | --- | --- | --- | --- |
|  | Crude | | | Energy-adjusted | | | Crude | | | Energy-adjusted | | |
|  | ICC (95% CI) | N | *I^2^* | ICC (95% CI) | N | *I^2^* | ICC (95% CI) | N | *I^2^* | ICC (95% CI) | N | *I^2^* |
| Energy | 0.758 (0.669, 0.826) | 39 | 97.4 | N/A | N/A | N/A | 0.642 (0.577, 0.699) | 22 | 92 | N/A | N/A | N/A |
| Protein | 0.679 (0.631, 0.721) | 40 | 87.5 | 0.573 (0.452, 0.672) | 14 | 89.3 | 0.613 (0.555, 0.664) | 23 | 88.8 | 0.619 (0.570, 0.664) | 11 | 53.5 |
| Fat | 0.650 (0.597, 0.697) | 37 | 87.3 | 0.492 (0.379, 0.590) | 12 | 80.7 | 0.635 (0.569, 0.692) | 18 | 91.2 | 0.654 (0.588, 0.711) | 7 | 72.7 |
| MUFA | 0.649 (0.588, 0.703) | 26 | 83.7 | 0.625 (0.470, 0.743) | 10 | 89.1 | 0.635 (0.590, 0.676) | 15 | 74.8 | 0.631 (0.553, 0.698) | 8 | 74.5 |
| PUFA | 0.682 (0.551, 0.780) | 27 | 96.5 | 0.599 (0.426, 0.730) | 10 | 90.4 | 0.590 (0.534, 0.642) | 18 | 84.2 | 0.554 (0.462, 0.635) | 8 | 76.5 |
| SFA | 0.732 (0.597, 0.826) | 31 | 97.6 | 0.656 (0.516, 0.762) | 11 | 89.9 | 0.637 (0.574, 0.692) | 18 | 89.6 | 0.625 (0.531, 0.704) | 8 | 82 |
| Linoleic acid | 0.537 (-0.01, 0.839) | 2 | 93.3 | N/A | N/A | N/A | 0.732 (0.626, 0.812) | 3 | 81.5 | 0.685 (0.591, 0.760) | 3 | 71 |
| Linolenic acid | 0.608 (0.169, 0.846) | 2 | 91.1 | N/A | N/A | N/A | 0.694 (0.653, 0.730) | 2 | 0 | 0.630 (0.527, 0.714) | 2 | 47.8 |
| Trans-fat | 0.706 (0.596, 0.790) | 2 | 36.6 | N/A | N/A | N/A | 0.485 (0.390, 0.570) | 2 | 0 | 0.473 (0.360, 0.574) | 1 | N/A |
| Cholesterol | 0.699 (0.631, 0.757) | 32 | 91.2 | 0.620 (0.510, 0.710) | 14 | 86.2 | 0.626 (0.557, 0.685) | 16 | 90.2 | 0.618 (0.548, 0.679) | 11 | 78 |
| Lipid | 0.859 (0.829, 0.885) | 1 | N/A | 0.849 (0.817, 0.876) | 1 | N/A | 0.619 (0.452, 0.744) | 3 | 66.7 | 0.551 (0.451, 0.637) | 3 | 0 |
| Carbohydrate | 0.702 (0.593, 0.786) | 41 | 97.7 | 0.616 (0.466, 0.731) | 14 | 93.6 | 0.652 (0.594, 0.704) | 21 | 91.1 | 0.669 (0.603, 0.726) | 9 | 77.3 |
| Sucrose | 0.603 (0.487, 0.697) | 3 | 67.9 | N/A | N/A | N/A | 0.709 (0.627, 0.776) | 1 | N/A | 0.679 (0.607, 0.741) | 1 | N/A |
| Sugar | 0.715 (0.547, 0.828) | 5 | 85.7 | N/A | N/A | N/A | 0.700 (0.593, 0.783) | 3 | 71.4 | 0.779 (0.747, 0.808) | 1 | N/A |
| Starch | 0.518 (0.057, 0.797) | 2 | 92.3 | N/A | N/A | N/A | 0.510 (0.351, 0.640) | 1 | N/A | N/A | N/A | N/A |
| Fiber | 0.693 (0.632, 0.746) | 36 | 90.4 | 0.612 (0.469, 0.723) | 12 | 92.5 | 0.671 (0.603, 0.729) | 18 | 92.9 | 0.718 (0.635, 0.784) | 9 | 88 |
| Alcohol | 0.814 (0.756, 0.860) | 13 | 82 | 0.800 (0.707, 0.865) | 3 | N/A | 0.788 (0.688, 0.859) | 9 | 92.5 | 0.803 (0.717, 0.865) | 6 | 89.5 |
| Vitamin A | 0.654 (0.562, 0.731) | 20 | 94.3 | 0.559 (0.379, 0.697) | 8 | 92.4 | 0.645 (0.536, 0.733) | 7 | 93.1 | 0.650 (0.464, 0.781) | 4 | 88.2 |
| Retinol | 0.605 (0.464, 0.715) | 9 | 91 | 0.414 (0.214, 0.581) | 4 | 52.7 | 0.582 (0.493, 0.658) | 9 | 72.6 | 0.568 (0.442, 0.673) | 5 | 70.9 |
| Vitamin C | 0.698 (0.603, 0.773) | 32 | 96.7 | 0.636 (0.431, 0.779) | 14 | 96.7 | 0.635 (0.552, 0.705) | 15 | 93.6 | 0.635 (0.514, 0.731) | 8 | 90.7 |
| Vitamin D | 0.676 (0.380, 0.846) | 11 | 98.9 | 0.770 (0.024, 0.965) | 2 | 98.8 | 0.681 (0.584, 0.759) | 5 | 94.8 | 0.586 (0.374, 0.739) | 3 | 93.6 |
| Vitamin E | 0.700 (0.549, 0.807) | 21 | 98.3 | 0.549 (0.248, 0.753) | 7 | 97.2 | 0.634 (0.567, 0.693) | 13 | 89.1 | 0.653 (0.569, 0.723) | 7 | 79.7 |
| Vitamin K | 0.590 (0.141, 0.838) | 2 | 92.7 | N/A | N/A | N/A | 0.710 (0.672, 0.745) | 2 | 0 | 0.693 (0.652, 0.729) | 2 | 0 |
| Thiamin | 0.622 (0.573, 0.667) | 23 | 80.2 | 0.551 (0.419, 0.661) | 8 | 88.6 | 0.643 (0.569, 0.707) | 8 | 89.7 | 0.696 (0.505, 0.822) | 4 | 95.2 |
| Riboflavin | 0.655 (0.602, 0.703) | 20 | 83.6 | 0.551 (0.256, 0.753) | 5 | 96.4 | 0.686 (0.600, 0.756) | 8 | 93.4 | 0.681 (0.543, 0.783) | 5 | 91.2 |
| Niacin | 0.642 (0.578, 0.698) | 17 | 85.2 | 0.475 (0.328, 0.599) | 5 | 80.1 | 0.732 (0.635, 0.806) | 5 | 88 | 0.702 (0.621, 0.769) | 5 | 83.1 |
| Vitamin B6 | 0.745 (0.360, 0.913) | 8 | 98.8 | 0.726 (0.101, 0.940) | 2 | 97.9 | 0.678 (0.568, 0.764) | 5 | 89.2 | 0.701 (0.492, 0.833) | 3 | 94 |
| Folate | 0.636 (0.568, 0.695) | 17 | 83.5 | 0.476 (0.379, 0.562) | 3 | 0 | 0.636 (0.537, 0.718) | 8 | 93.8 | 0.687 (0.564, 0.781) | 3 | 77.7 |
| Vitamin B12 | 0.695 (0.391, 0.862) | 8 | 98.2 | 0.734 (0.316, 0.913) | 3 | 97.3 | 0.643 (0.511, 0.745) | 5 | 91.3 | 0.633 (0.510, 0.731) | 4 | 86.6 |
| Carotene | 0.501 (0.311, 0.652) | 5 | 91.9 | 0.229 (0.097, 0.353) | 1 | N/A | 0.704 (0.630, 0.766) | 4 | 86.5 | 0.586 (0.519, 0.646) | 3 | 0 |
| β-Carotene | 0.756 (0.679, 0.817) | 10 | 81.5 | N/A | N/A | N/A | 0.644 (0.589, 0.694) | 9 | 62.3 | 0.613 (0.456, 0.733) | 4 | 81.9 |
| Se | 0.656 (0.596, 0.708) | 10 | 71.9 | 0.576 (0.346, 0.741) | 3 | 85.3 | 0.720 (0.610, 0.802) | 1 | N/A | 0.619 (0.482, 0.727) | 1 | N/A |
| Mg | 0.694 (0.620, 0.756) | 14 | 87.5 | 0.649 (0.410, 0.805) | 2 | 82.2 | 0.623 (0.457, 0.746) | 5 | 91.9 | 0.601 (0.423, 0.735) | 4 | 91.3 |
| Ca | 0.641 (0.593, 0.685) | 32 | 83.6 | 0.619 (0.476, 0.730) | 13 | 92.7 | 0.625 (0.548, 0.691) | 20 | 94.2 | 0.650 (0.568, 0.720) | 10 | 88 |
| Fe | 0.626 (0.567, 0.678) | 26 | 86.3 | 0.555 (0.446, 0.648) | 13 | 86.2 | 0.653 (0.569, 0.723) | 13 | 93.4 | 0.583 (0.518, 0.641) | 6 | 33.4 |
| Zn | 0.577 (0.530, 0.621) | 17 | 61.9 | 0.497 (0.334, 0.632) | 6 | 78.5 | 0.618 (0.544, 0.682) | 9 | 75.8 | 0.613 (0.539, 0.678) | 6 | 52.5 |
| Cu | 0.676 (0.610, 0.732) | 3 | N/A | N/A | N/A | N/A | 0.649 (0.602, 0.692) | 1 | N/A | 0.690 (0.646, 0.728) | 1 | N/A |
| K | 0.687 (0.611, 0.752) | 19 | 90.8 | 0.590 (0.288, 0.785) | 4 | 95 | 0.642 (0.522, 0.737) | 6 | 95.2 | 0.685 (0.647, 0.720) | 3 | 0 |
| P | 0.633 (0.502, 0.735) | 13 | 91.6 | 0.750 (0.643, 0.828) | 1 | N/A | 0.586 (0.481, 0.674) | 10 | 88.1 | 0.618 (0.518, 0.700) | 8 | 80.8 |
| Na | 0.692 (0.489, 0.823) | 20 | 98.7 | 0.683 (0.368, 0.857) | 6 | 97.8 | 0.534 (0.340, 0.685) | 5 | 91.9 | 0.642 (0.530, 0.732) | 2 | 63.9 |

* CI, confidence interval; N/A: not available
