# Supplementary material for: A meta-analysis of the reproducibility of food frequency questionnaires in nutritional epidemiological studies
Source: Int J Behav Nutr Phys Act. 2021 Jan 11;18:12. doi: 10.1186/s12966-020-01078-4 (PMC7802360; doi:10.1186/s12966-020-01078-4)
Supplement: Supplementary file 16 — Additional file 16 Supplemental Table 15. Pooled spearman correlation coefficient for energy and nutrients stratified by time interval (12 months as cut-point). [file 12966_2020_1078_MOESM16_ESM.docx]

**Supplemental Table 15.** **Pooled spearman correlation coefficient for energy and nutrients stratified by time interval (12 months as cut-point) ***

| Nutrient | < 12 months | | | | | | ≥ 12 months | | | | | |
| --- | --- | --- | --- | --- | --- | --- | --- | --- | --- | --- | --- | --- |
|  | Crude | | | Energy-adjusted | | | Crude | | | Energy-adjusted | | |
|  | SCC (95% CI) | N | *I^2^* | SCC (95% CI) | N | *I^2^* | SCC (95% CI) | N | *I^2^* | SCC (95% CI) | N | *I^2^* |
| Energy | 0.654 (0.621, 0.684) | 72 | 85 | N/A | N/A | N/A | 0.620 (0.587, 0.651) | 36 | 80.1 | N/A | N/A | N/A |
| Protein | 0.611 (0.575, 0.644) | 72 | 85 | 0.577 (0.515, 0.633) | 30 | 85.8 | 0.597 (0.568, 0.624) | 36 | 70.5 | 0.529 (0.489, 0.567) | 34 | 69.1 |
| Fat | 0.629 (0.598, 0.658) | 73 | 81.4 | 0.577 (0.505, 0.641) | 26 | 87.4 | 0.603 (0.577, 0.628) | 33 | 64.2 | 0.527 (0.486, 0.564) | 30 | 66.7 |
| Plant fat | 0.605 (0.513, 0.683) | 5 | 44.7 | N/A | N/A | N/A | 0.464 (0.406, 0.518) | 3 | 0 | N/A | N/A | N/A |
| Animal fat | 0.685 (0.664, 0.705) | 4 | 0 | N/A | N/A | N/A | 0.512 (0.376, 0.626) | 2 | N/A | 0.374 (0.221, 0.510) | 1 | N/A |
| MUFA | 0.655 (0.611, 0.695) | 25 | 69 | 0.606 (0.500, 0.694) | 12 | 86.4 | 0.577 (0.544, 0.609) | 36 | 49.8 | 0.509 (0.448, 0.564) | 20 | 69.3 |
| PUFA | 0.638 (0.602, 0.671) | 34 | 73.5 | 0.579 (0.492, 0.655) | 13 | 83.8 | 0.564 (0.534, 0.591) | 25 | 43.7 | 0.481 (0.422, 0.537) | 18 | 61.7 |
| n-3 PUFA | 0.699 (0.544, 0.809) | 1 | N/A | 0.450 (0.223, 0.630) | 1 | N/A | 0.613 (0.567, 0.656) | 5 | 60.4 | 0.472 (0.398, 0.540) | 4 | 44.6 |
| n-6 PUFA | 0.570 (0.371, 0.718) | 1 | N/A | 0.450 (0.223, 0.630) | 1 | N/A | 0.596 (0.562, 0.627) | 5 | 26 | 0.434 (0.341, 0.520) | 4 | 62.1 |
| SFA | 0.660 (0.630, 0.688) | 36 | 68.6 | 0.601 (0.522, 0.670) | 15 | 85.1 | 0.597 (0.559, 0.632) | 31 | 76.5 | 0.543 (0.481, 0.600) | 22 | 79.1 |
| Linoleic acid | 0.646 (0.606, 0.684) | 4 | 65.7 | 0.592 (0.476, 0.688) | 5 | 88.3 | 0.558 (0.507, 0.603) | 7 | 20.8 | 0.518 (0.400, 0.619) | 4 | 65 |
| Linolenic acid | 0.630 (0.592, 0.666) | 3 | 53.3 | 0.642 (0.486, 0.759) | 3 | 91 | 0.619 (0.568, 0.665) | 2 | 0 | 0.521 (0.451, 0.585) | 1 | N/A |
| Trans-fat | 0.667 (0.532, 0.769) | 5 | 78.8 | 0.789 (0.619, 0.889) | 1 | N/A | 0.339 (0.210, 0.455) | 1 | N/A | 0.296 (0.166, 0.417) | 1 | N/A |
| Cholesterol | 0.653 (0.621, 0.683) | 38 | 71.3 | 0.640 (0.570, 0.700) | 15 | 83.3 | 0.575 (0.532, 0.615) | 30 | 80.1 | 0.488 (0.429, 0.543) | 22 | 72.4 |
| Lipid | 0.532 (0.458, 0.599) | 2 | 0 | 0.820 (0.669, 0.905) | 1 | N/A | 0.581 (0.491, 0.659) | 4 | 0 | 0.459 (0.276, 0.610) | 3 | 63.7 |
| Carbohydrate | 0.627 (0.590, 0.661) | 67 | 86.5 | 0.619 (0.562, 0.671) | 26 | 84.5 | 0.608 (0.570, 0.645) | 36 | 85.3 | 0.546 (0.496, 0.592) | 34 | 81.4 |
| Sucrose | 0.656 (0.452, 0.794) | 3 | 81.6 | 0.632 (0.513, 0.726) | 1 | N/A | 0.730 (0.667, 0.782) | 4 | 56.3 | N/A | N/A | N/A |
| Sugar | 0.691 (0.619, 0.751) | 9 | 81.2 | 0.639 (0.516, 0.737) | 4 | 88.6 | 0.670 (0.592, 0.735) | 2 | 69.1 | 0.779 (0.747, 0.808) | 1 | 27.2 |
| starch | 0.628 (0.497, 0.731) | 4 | 74.2 | 0.606 (0.553, 0.654) | 2 | 0 | 0.636 (0.587, 0.681) | 3 | 0 | N/A | N/A | N/A |
| Fiber | 0.641 (0.605, 0.674) | 57 | 82.2 | 0.642 (0.579, 0.698) | 24 | 86.8 | 0.623 (0.578, 0.663) | 32 | 84.9 | 0.606 (0.553, 0.653) | 29 | 78.3 |
| Soluble fiber | 0.710 (0.492, 0.845) | 4 | 87.4 | 0.775 (0.439, 0.921) | 2 | 85.3 | 0.648 (0.592, 0.697) | 10 | 75.8 | 0.546 (0.455, 0.626) | 8 | 69 |
| Insoluble fiber | 0.693 (0.486, 0.826) | 4 | 85.3 | 0.727 (0.510, 0.857) | 2 | 64.4 | 0.634 (0.592, 0.672) | 8 | 0 | 0.583 (0.501, 0.654) | 10 | 74.9 |
| Alcohol | 0.845 (0.816, 0.870) | 32 | 90.3 | 0.801 (0.729, 0.856) | 11 | 91.9 | 0.848 (0.787, 0.893) | 17 | 95.4 | 0.786 (0.713, 0.842) | 16 | 91 |
| Vitamin A | 0.632 (0.566, 0.689) | 29 | 90.2 | 0.567 (0.413, 0.690) | 12 | 93.5 | 0.565 (0.534, 0.594) | 13 | 41.8 | 0.534 (0.477, 0.588) | 10 | 66 |
| Retinol | 0.594 (0.551, 0.635) | 25 | 81 | 0.537 (0.429, 0.630) | 14 | 91.5 | 0.551 (0.512, 0.588) | 26 | 69.2 | 0.504 (0.460, 0.545) | 24 | 63.4 |
| Vitamin C | 0.622 (0.586, 0.655) | 43 | 83.4 | 0.610 (0.542, 0.670) | 22 | 87.8 | 0.603 (0.560, 0.642) | 51 | 86.4 | 0.566 (0.520, 0.610) | 35 | 76.7 |
| Vitamin D | 0.583 (0.515, 0.643) | 17 | 83.7 | 0.610 (0.546, 0.667) | 7 | 39.7 | 0.605 (0.524, 0.676) | 13 | 86 | 0.524 (0.370, 0.650) | 8 | 83.5 |
| Vitamin E | 0.591 (0.524, 0.650) | 25 | 93.3 | 0.573 (0.470, 0.660) | 12 | 91 | 0.628 (0.578, 0.673) | 29 | 85.6 | 0.533 (0.456, 0.603) | 18 | 76.3 |
| Vitamin K | 0.618 (0.479, 0.728) | 4 | 73.8 | 0.753 (0.572, 0.864) | 2 | 36.2 | 0.583 (0.471, 0.677) | 3 | 0 | 0.610 (0.503, 0.699) | 3 | 0 |
| Thiamin | 0.635 (0.600, 0.668) | 33 | 76.9 | 0.540 (0.459, 0.613) | 16 | 83.8 | 0.590 (0.557, 0.622) | 24 | 66.9 | 0.496 (0.443, 0.546) | 23 | 73 |
| Riboflavin | 0.654 (0.615, 0.690) | 33 | 84 | 0.600 (0.505, 0.681) | 15 | 90.9 | 0.626 (0.592, 0.658) | 23 | 72.2 | 0.554 (0.508, 0.597) | 20 | 65.5 |
| Niacin | 0.621 (0.536, 0.695) | 18 | 89.9 | 0.572 (0.476, 0.654) | 14 | 85.3 | 0.656 (0.556, 0.737) | 21 | 95 | 0.437 (0.353, 0.513) | 20 | 86 |
| Vitamin B6 | 0.564 (0.451, 0.660) | 17 | 85.6 | 0.646 (0.559, 0.720) | 7 | 56.1 | 0.608 (0.553, 0.657) | 10 | 57.7 | 0.489 (0.392, 0.574) | 12 | 77.3 |
| Folate | 0.591 (0.528, 0.647) | 31 | 86.8 | 0.642 (0.538, 0.726) | 12 | 86.7 | 0.596 (0.552, 0.637) | 18 | 72.2 | 0.573 (0.502, 0.637) | 14 | 71.6 |
| Vitamin B12 | 0.634 (0.551, 0.705) | 13 | 83.9 | 0.672 (0.545, 0.769) | 8 | 87.2 | 0.582 (0.505, 0.650) | 15 | 73.6 | 0.488 (0.416, 0.554) | 13 | 61.5 |
| Carotene | 0.615 (0.561, 0.663) | 8 | 87.5 | 0.549 (0.362, 0.694) | 5 | 96.3 | 0.586 (0.521, 0.643) | 19 | 86.6 | 0.489 (0.424, 0.549) | 16 | 68.9 |
| α-Carotene | 0.248 (-0.01, 0.480) | 1 | N/A | N/A | N/A | N/A | 0.534 (0.463, 0.598) | 0 | 57.1 | 0.470 (0.411, 0.527) | 0 | 31.9 |
| β-Carotene | 0.621 (0.559, 0.677) | 15 | 67.9 | 0.610 (0.536, 0.675) | 9 | 58.1 | 0.597 (0.552, 0.638) | 24 | 71.3 | 0.519 (0.482, 0.553) | 19 | 33.8 |
| Se | 0.702 (0.599, 0.782) | 8 | 89.8 | 0.719 (0.471, 0.862) | 4 | 92.9 | 0.583 (0.533, 0.630) | 7 | 9.7 | 0.466 (0.411, 0.519) | 7 | 0 |
| Mg | 0.662 (0.584, 0.728) | 19 | 89.8 | 0.664 (0.573, 0.738) | 10 | 81.2 | 0.600 (0.443, 0.722) | 11 | 89.2 | 0.563 (0.373, 0.708) | 9 | 89.6 |
| Ca | 0.627 (0.595, 0.657) | 44 | 80.7 | 0.619 (0.552, 0.677) | 23 | 87.6 | 0.592 (0.548, 0.633) | 45 | 83.9 | 0.559 (0.510, 0.604) | 32 | 76.6 |
| Fe | 0.594 (0.550, 0.634) | 41 | 87.7 | 0.613 (0.536, 0.679) | 20 | 88.8 | 0.617 (0.585, 0.647) | 36 | 66.9 | 0.528 (0.484, 0.570) | 27 | 65.4 |
| I | 0.828 (0.724, 0.894) | 2 | 19.8 | 0.744 (0.600, 0.841) | 1 | N/A | N/A | N/A | N/A | N/A | N/A | N/A |
| Zn | 0.616 (0.538, 0.683) | 17 | 87.2 | 0.626 (0.507, 0.722) | 11 | 89 | 0.635 (0.544, 0.712) | 9 | 78.5 | 0.529 (0.440, 0.606) | 7 | 50.1 |
| Cu | 0.796 (0.657, 0.883) | 4 | 87.4 | 0.742 (0.564, 0.854) | 3 | 92 | 0.619 (0.508, 0.710) | 2 | N/A | 0.695 (0.601, 0.770) | 3 | N/A |
| K | 0.669 (0.624, 0.708) | 30 | 84.6 | 0.656 (0.596, 0.707) | 14 | 71.1 | 0.616 (0.582, 0.648) | 21 | 66.4 | 0.557 (0.508, 0.601) | 20 | 66.3 |
| P | 0.683 (0.614, 0.742) | 16 | 81.9 | 0.639 (0.533, 0.725) | 10 | 83.8 | 0.587 (0.529, 0.639) | 27 | 82.9 | 0.534 (0.477, 0.586) | 20 | 75.1 |
| Na | 0.621 (0.571, 0.666) | 22 | 87.2 | 0.564 (0.456, 0.656) | 15 | 91.9 | 0.603 (0.562, 0.640) | 21 | 57.7 | 0.516 (0.469, 0.561) | 15 | 55.8 |
| Mn | 0.625 (0.544, 0.694) | 3 | 0 | 0.703 (0.542, 0.814) | 1 | N/A | 0.704 (0.612, 0.777) | 2 | N/A | 0.724 (0.638, 0.793) | 1 | N/A |

* CI, confidence interval; N/A: not available
