# Supplementary material for: A meta-analysis of the reproducibility of food frequency questionnaires in nutritional epidemiological studies
Source: Int J Behav Nutr Phys Act. 2021 Jan 11;18:12. doi: 10.1186/s12966-020-01078-4 (PMC7802360; doi:10.1186/s12966-020-01078-4)
Supplement: Supplementary file 17 — Additional file 17 Supplemental Table 16. Pooled intraclass correlation coefficient for energy and nutrients stratified by items of FFQ. [file 12966_2020_1078_MOESM17_ESM.docx]

**Supplemental Table 16. Pooled intraclass correlation coefficient for energy and nutrients stratified by items of FFQ**

| Nutrient | ≥ 120 | | | | | | < 120 | | | | | |
| --- | --- | --- | --- | --- | --- | --- | --- | --- | --- | --- | --- | --- |
|  | Crude | | | Energy-adjusted | | | Crude | | | Energy-adjusted | | |
|  | ICC (95% CI) | N | *I^2^* | ICC (95% CI) | N | *I^2^* | ICC (95% CI) | N | *I^2^* | ICC (95% CI) | N | *I^2^* |
| Energy | 0.721 (0.620, 0.799) | 34 | 97.6 | N/A | N/A | N/A | 0.692 (0.636, 0.740) | 27 | 90.2 | N/A | N/A | N/A |
| Protein | 0.653 (0.603, 0.697) | 32 | 86.5 | 0.643 (0.582, 0.697) | 14 | 75.6 | 0.644 (0.588, 0.694) | 31 | 89.9 | 0.544 (0.464, 0.616) | 11 | 72.5 |
| Fat | 0.679 (0.624, 0.728) | 25 | 87.8 | 0.631 (0.560, 0.692) | 8 | 66.6 | 0.613 (0.541, 0.675) | 30 | 92.7 | 0.510 (0.384, 0.618) | 11 | 87.4 |
| Plant fat | 0.572 (0.461, 0.665) | 5 | 60 | N/A | N/A | N/A | N/A | N/A | N/A | N/A | N/A | N/A |
| Animal fat | N/A | N/A | N/A | N/A | N/A | N/A | N/A | N/A | N/A | N/A | N/A | N/A |
| MUFA | 0.656 (0.613, 0.696) | 26 | 73 | 0.645 (0.565, 0.712) | 13 | 82.5 | 0.616 (0.543, 0.679) | 15 | 87.9 | 0.594 (0.406, 0.734) | 5 | 86.3 |
| PUFA | 0.685 (0.578, 0.769) | 26 | 95.9 | 0.609 (0.516, 0.687) | 13 | 85.1 | 0.577 (0.503, 0.644) | 19 | 89 | 0.476 (0.346, 0.588) | 5 | 64.4 |
| n-3 PUFA | N/A | N/A | N/A | N/A | N/A | N/A | N/A | N/A | N/A | N/A | N/A | N/A |
| n-6 PUFA | N/A | N/A | N/A | N/A | N/A | N/A | N/A | N/A | N/A | N/A | N/A | N/A |
| SFA | 0.715 (0.583, 0.810) | 27 | 97.6 | 0.658 (0.578, 0.726) | 13 | 83.9 | 0.647 (0.575, 0.709) | 22 | 91.4 | 0.603 (0.408, 0.746) | 6 | 89.7 |
| Linoleic acid | 0.727 (0.615, 0.809) | 3 | 80.2 | 0.689 (0.551, 0.790) | 2 | 85.2 | 0.557 (-0.03, 0.859) | 2 | 95.7 | 0.679 (0.533, 0.786) | 1 | N/A |
| Linolenic acid | 0.695 (0.654, 0.731) | 2 | 0 | N/A | N/A | N/A | 0.590 (0.205, 0.817) | 2 | 91.5 | N/A | N/A | N/A |
| EPA | N/A | N/A | N/A | N/A | N/A | N/A | N/A | N/A | N/A | N/A | N/A | N/A |
| DHA | N/A | N/A | N/A | N/A | N/A | N/A | N/A | N/A | N/A | N/A | N/A | N/A |
| Trans-fat | 0.646 (0.301, 0.841) | 2 | 88 | N/A | N/A | N/A | 0.573 (0.310, 0.754) | 2 | 82.5 | N/A | N/A | N/A |
| Cholesterol | 0.684 (0.618, 0.741) | 27 | 90 | 0.641 (0.558, 0.711) | 15 | 85.7 | 0.621 (0.546, 0.686) | 21 | 91.5 | 0.586 (0.509, 0.654) | 10 | 70.2 |
| Lipid | 0.701 (0.459, 0.846) | 4 | 93.4 | 0.662 (0.370, 0.835) | 4 | 94.5 | N/A | N/A | N/A | N/A | N/A | N/A |
| Carbohydrate | 0.686 (0.564, 0.779) | 31 | 98.1 | 0.680 (0.584, 0.757) | 10 | 0 | 0.670 (0.615, 0.719) | 31 | 89.9 | 0.603 (0.503, 0.686) | 1 | N/A |
| Sucrose | 0.699 (0.602, 0.777) | 1 | N/A | N/A | N/A | N/A | 0.610 (0.480, 0.713) | 3 | 76.8 | N/A | N/A | N/A |
| Sugar | 0.693 (0.610, 0.762) | 4 | 60.9 | N/A | N/A | N/A | 0.730 (0.506, 0.861) | 4 | 89.3 | N/A | N/A | N/A |
| Starch | 0.601 (0.393, 0.750) | 2 | 67.9 | N/A | N/A | N/A | 0.310 (0.174, 0.434) | 1 | N/A | N/A | N/A | N/A |
| Fiber | 0.690 (0.621, 0.749) | 24 | 90.7 | 0.701 (0.612, 0.773) | 11 | 89.9 | 0.677 (0.615, 0.731) | 30 | 93 | 0.634 (0.503, 0.737) | 10 | 90.9 |
| Soluble fiber | N/A | N/A | N/A | N/A | N/A | N/A | N/A | N/A | N/A | N/A | N/A | N/A |
| Insoluble fiber | N/A | N/A | N/A | N/A | N/A | N/A | N/A | N/A | N/A | N/A | N/A | N/A |
| Alcohol | 0.825 (0.762, 0.873) | 13 | 88.2 | 0.808 (0.694, 0.883) | 6 | 88.8 | 0.764 (0.697, 0.819) | 9 | 78.3 | 0.791 (0.680, 0.867) | 3 | 80.4 |
| Vitamin A | 0.660 (0.507, 0.772) | 12 | 96.4 | 0.669 (0.514, 0.782) | 7 | 91.8 | 0.594 (0.507, 0.667) | 15 | 91.2 | 0.479 (0.275, 0.641) | 5 | 87.6 |
| Retinol | 0.567 (0.464, 0.656) | 11 | 83.1 | 0.517 (0.271, 0.699) | 4 | 72.2 | 0.620 (0.534, 0.694) | 7 | 71.3 | 0.548 (0.401, 0.667) | 5 | 79.9 |
| Carotene | 0.512 (0.476, 0.546) | 3 | 0 | 0.599 (0.471, 0.703) | 2 | 0 | 0.672 (0.539, 0.773) | 6 | 95.9 | 0.456 (0.172, 0.671) | 3 | 91.9 |
| β-Carotene | 0.673 (0.590, 0.742) | 11 | 79.9 | 0.577 (0.371, 0.728) | 5 | 83.8 | 0.678 (0.626, 0.723) | 8 | 51.5 | 0.750 (0.628, 0.835) | 1 | N/A |
| Vitamin C | 0.679 (0.571, 0.764) | 25 | 97 | 0.723 (0.612, 0.806) | 11 | 93.2 | 0.679 (0.571, 0.764) | 22 | 94.1 | 0.530 (0.377, 0.655) | 11 | 91.8 |
| Vitamin D | 0.709 (0.436, 0.863) | 8 | 99.2 | 0.709 (0.404, 0.872) | 4 | 98.5 | 0.648 (0.548, 0.730) | 8 | 92.5 | 0.469 (0.311, 0.603) | 1 | N/A |
| Vitamin E | 0.691 (0.523, 0.806) | 18 | 98.5 | 0.659 (0.505, 0.772) | 8 | 94.6 | 0.637 (0.566, 0.699) | 16 | 90.2 | 0.537 (0.360, 0.677) | 7 | 91.4 |
| Vitamin K | 0.634 (0.353, 0.810) | 3 | 98.1 | N/A | N/A | N/A | 0.720 (0.586, 0.815) | 1 | N/A | N/A | N/A | N/A |
| Thiamin | 0.668 (0.594, 0.731) | 15 | 91.7 | 0.730 (0.613, 0.816) | 6 | 93.1 | 0.594 (0.542, 0.641) | 16 | 77.5 | 0.439 (0.376, 0.498) | 6 | 19.3 |
| Riboflavin | 0.698 (0.638, 0.750) | 12 | 87.6 | 0.766 (0.666, 0.839) | 4 | 91.3 | 0.641 (0.555, 0.712) | 16 | 92.9 | 0.480 (0.361, 0.583) | 6 | 75.4 |
| Niacin | 0.699 (0.603, 0.776) | 10 | 93.4 | 0.673 (0.585, 0.746) | 3 | 69.6 | 0.641 (0.573, 0.699) | 12 | 77.8 | 0.569 (0.396, 0.703) | 7 | 92.8 |
| Vitamin B6 | 0.782 (0.543, 0.904) | 9 | 98.7 | 0.756 (0.559, 0.872) | 4 | 97 | 0.537 (0.446, 0.616) | 4 | 51.6 | 0.459 (0.274, 0.611) | 1 | N/A |
| Folate | 0.618 (0.549, 0.679) | 13 | 86.5 | 0.589 (0.445, 0.703) | 3 | 79.5 | 0.656 (0.566, 0.731) | 12 | 91.7 | 0.610 (0.385, 0.766) | 3 | 82.5 |
| Vitamin B12 | 0.717 (0.499, 0.850) | 9 | 98.3 | 0.717 (0.488, 0.853) | 5 | 97.8 | 0.568 (0.462, 0.659) | 4 | 60.9 | 0.582 (0.474, 0.672) | 2 | 0 |
| Se | 0.704 (0.660, 0.744) | 7 | 38.5 | 0.619 (0.422, 0.761) | 3 | 82.2 | 0.581 (0.487, 0.662) | 4 | 55.9 | 0.480 (0.323, 0.611) | 1 | N/A |
| Mg | 0.685 (0.599, 0.755) | 12 | 91.7 | 0.605 (0.454, 0.722) | 5 | 89.8 | 0.654 (0.564, 0.728) | 7 | 72.7 | 0.679 (0.551, 0.777) | 1 | N/A |
| Ca | 0.598 (0.537, 0.654) | 25 | 87.5 | 0.622 (0.515, 0.710) | 12 | 89.2 | 0.664 (0.598, 0.721) | 27 | 93.2 | 0.664 (0.538, 0.760) | 11 | 92.9 |
| Iron | 0.616 (0.552, 0.673) | 17 | 84.9 | 0.568 (0.478, 0.645) | 9 | 74.6 | 0.656 (0.579, 0.721) | 22 | 93.6 | 0.562 (0.447, 0.659) | 10 | 83.8 |
| I | N/A | N/A | N/A | N/A | N/A | N/A | N/A | N/A | N/A | N/A | N/A | N/A |
| Zn | 0.596 (0.537, 0.648) | 14 | 75.3 | 0.577 (0.523, 0.627) | 7 | 13.2 | 0.594 (0.539, 0.644) | 12 | 59.3 | 0.581 (0.396, 0.720) | 5 | 87.3 |
| Cu | 0.648 (0.603, 0.690) | 2 | 0 | N/A | N/A | N/A | 0.682 (0.612, 0.742) | 2 | 0 | N/A | N/A | N/A |
| K | 0.633 (0.537, 0.712) | 13 | 92.6 | 0.542 (0.289, 0.723) | 4 | 95.7 | 0.710 (0.637, 0.771) | 12 | 90.5 | 0.746 (0.688, 0.794) | 3 | 0 |
| P | 0.592 (0.488, 0.680) | 14 | 91.8 | 0.626 (0.505, 0.723) | 7 | 83.1 | 0.622 (0.473, 0.737) | 9 | 90.3 | 0.667 (0.465, 0.804) | 2 | 83.1 |
| Na | 0.696 (0.430, 0.851) | 12 | 99.1 | 0.731 (0.517, 0.859) | 4 | 96.8 | 0.606 (0.492, 0.700) | 13 | 88.5 | 0.597 (0.210, 0.822) | 4 | 96.4 |
| Mn | N/A | N/A | N/A | N/A | N/A | N/A | N/A | N/A | N/A | N/A | N/A | N/A |
