# Supplementary material for: A meta-analysis of the reproducibility of food frequency questionnaires in nutritional epidemiological studies
Source: Int J Behav Nutr Phys Act. 2021 Jan 11;18:12. doi: 10.1186/s12966-020-01078-4 (PMC7802360; doi:10.1186/s12966-020-01078-4)
Supplement: Supplementary file 18 — Additional file 18 Supplemental Table 17. Pooled spearman correlation coefficient for energy and nutrients stratified by items of FFQ. [file 12966_2020_1078_MOESM18_ESM.docx]

**Supplemental Table 17. Pooled spearman correlation coefficient for energy and nutrients stratified by items of FFQ***

| Nutrient | ≥ 120 | | | | | | < 120 | | | | | |
| --- | --- | --- | --- | --- | --- | --- | --- | --- | --- | --- | --- | --- |
|  | Crude | | | Energy-adjusted | | | Crude | | | Energy-adjusted | | |
|  | SCC (95% CI) | N | *I^2^* | SCC (95% CI) | N | *I^2^* | SCC (95% CI) | N | *I^2^* | SCC (95% CI) | N | *I^2^* |
| Energy | 0.686 (0.650, 0.720) | 46 | 83.6 | N/A | N/A | N/A | 0.606 (0.572, 0.638) | 64 | 83.2 | N/A | N/A | N/A |
| Protein | 0.648 (0.610, 0.683) | 45 | 80.8 | 0.596 (0.538, 0.649) | 27 | 83.3 | 0.568 (0.534, 0.601) | 65 | 81 | 0.525 (0.481, 0.566) | 37 | 72.5 |
| Fat | 0.654 (0.621, 0.685) | 43 | 75.2 | 0.587 (0.519, 0.648) | 20 | 83 | 0.591 (0.560, 0.621) | 65 | 79.2 | 0.534 (0.487, 0.578) | 36 | 76.9 |
| Plant fat | 0.581 (0.353, 0.744) | 1 | N/A | N/A | N/A | N/A | 0.547 (0.458, 0.625) | 5 | 68.5 | N/A | N/A | N/A |
| Animal fat | 0.750 (0.673, 0.810) | 1 | N/A | N/A | N/A | N/A | 0.683 (0.648, 0.715) | 3 | 0 | N/A | N/A | N/A |
| MUFA | 0.643 (0.602, 0.681) | 34 | 97.6 | 0.566 (0.494, 0.630) | 16 | 80.1 | 0.571 (0.534, 0.605) | 27 | 90.2 | 0.535 (0.448, 0.611) | 16 | 80.6 |
| PUFA | 0.608 (0.560, 0.652) | 30 | 77.2 | 0.573 (0.499, 0.639) | 14 | 77.8 | 0.580 (0.544, 0.614) | 27 | 66 | 0.474 (0.405, 0.538) | 17 | 72.6 |
| n-3 PUFA | 0.634 (0.527, 0.722) | 1 | N/A | 0.557 (0.434, 0.659) | 1 | N/A | 0.618 (0.566, 0.665) | 5 | 66.8 | 0.449 (0.373, 0.519) | 4 | 38.7 |
| n-6 PUFA | 0.604 (0.424, 0.738) | 1 | 56.5 | 0.507 (0.323, 0.655) | 1 | 47.4 | 0.594 (0.562, 0.624) | 5 | 19.2 | 0.424 (0.323, 0.514) | 4 | 61.8 |
| SFA | 0.681 (0.643, 0.715) | 28 | 71.2 | 0.608 (0.546, 0.664) | 16 | 76.7 | 0.583 (0.546, 0.617) | 37 | 76.9 | 0.533 (0.471, 0.589) | 21 | 78.8 |
| Linoleic acid | 0.721 (0.542, 0.837) | 2 | 83.1 | 0.620 (0.484, 0.727) | 5 | 85.8 | 0.585 (0.522, 0.642) | 7 | 76.6 | 0.519 (0.401, 0.619) | 4 | 84.5 |
| Linolenic acid | 0.809 (0.731, 0.867) | 1 | N/A | 0.681 (0.468, 0.819) | 3 | 91.8 | 0.621 (0.580, 0.660) | 2 | 0 | 0.521 (0.451, 0.585) | 1 | N/A |
| EPA | 0.846 (0.713, 0.920) | 2 | 81.8 | N/A | N/A | N/A | 0.549 (0.265, 0.746) | 1 | N/A | N/A | N/A | N/A |
| DHA | 0.797 (0.736, 0.845) | 2 | 0 | N/A | N/A | N/A | 0.549 (0.265, 0.746) | 1 | N/A | N/A | N/A | N/A |
| Trans-fat | 0.618 (0.393, 0.772) | 5 | 92.8 | N/A | N/A | N/A | 0.599 (0.333, 0.777) | 1 | N/A | N/A | N/A | N/A |
| Cholesterol | 0.648 (0.590, 0.699) | 28 | 83.6 | 0.605 (0.516, 0.681) | 14 | 86.6 | 0.591 (0.558, 0.623) | 38 | 74.8 | 0.522 (0.465, 0.576) | 23 | 76.7 |
| Lipid | 0.555 (0.497, 0.608) | 5 | 0 | 0.459 (0.276, 0.610) | 3 | 63.7 | 0.469 (0.162, 0.694) | 1 | N/A | 0.820 (0.669, 0.905) | 1 | N/A |
| Carbohydrate | 0.654 (0.609, 0.694) | 44 | 86.8 | 0.598 (0.544, 0.647) | 27 | 80.2 | 0.611 (0.574, 0.646) | 61 | 86.3 | 0.576 (0.521, 0.626) | 33 | 84.7 |
| Sucrose | 0.656 (0.452, 0.794) | 3 | 81.6 | N/A | N/A | N/A | 0.730 (0.667, 0.782) | 4 | 56.3 | N/A | N/A | N/A |
| Sugar | 0.707 (0.653, 0.754) | 8 | 58.9 | 0.706 (0.656, 0.750) | 3 | 15.1 | 0.645 (0.369, 0.816) | 3 | 93.2 | 0.549 (0.019, 0.837) | 2 | 96.7 |
| Starch | 0.636 (0.530, 0.723) | 2 | 0 | N/A | N/A | N/A | 0.642 (0.602, 0.678) | 2 | 0 | N/A | N/A | N/A |
| Fiber | 0.653 (0.601, 0.698) | 38 | 84.9 | 0.671 (0.621, 0.716) | 22 | 79.7 | 0.629 (0.590, 0.664) | 49 | 84.5 | 0.580 (0.527, 0.628) | 31 | 79.9 |
| Soluble fiber | 0.659 (0.468, 0.792) | 4 | 85 | 0.634 (0.527, 0.722) | 2 | 0 | 0.659 (0.603, 0.709) | 10 | 76.8 | 0.581 (0.461, 0.679) | 8 | 81.3 |
| Insoluble fiber | 0.676 (0.490, 0.804) | 4 | 85.2 | 0.677 (0.624, 0.723) | 4 | 0 | 0.635 (0.592, 0.675) | 8 | 0 | 0.558 (0.453, 0.647) | 8 | 74.2 |
| Alcohol | 0.851 (0.803, 0.889) | 21 | 93 | 0.790 (0.722, 0.844) | 12 | 90.5 | 0.851 (0.808, 0.884) | 26 | 94 | 0.795 (0.730, 0.846) | 15 | 90.2 |
| Vitamin A | 0.677 (0.601, 0.741) | 19 | 91.9 | 0.657 (0.547, 0.744) | 7 | 88.7 | 0.553 (0.513, 0.590) | 23 | 67.1 | 0.494 (0.393, 0.584) | 15 | 85.9 |
| Retinol | 0.559 (0.476, 0.632) | 17 | 89 | 0.494 (0.350, 0.615) | 11 | 91.4 | 0.577 (0.540, 0.610) | 32 | 67.2 | 0.519 (0.465, 0.568) | 27 | 78.2 |
| Carotene | 0.654 (0.608, 0.696) | 38 | 85.8 | 0.663 (0.614, 0.707) | 21 | 75.6 | 0.584 (0.545, 0.621) | 58 | 85.3 | 0.551 (0.502, 0.596) | 36 | 78.3 |
| β-Carotene | 0.609 (0.544, 0.667) | 17 | 83.5 | 0.515 (0.363, 0.639) | 7 | 89.7 | 0.581 (0.503, 0.649) | 17 | 85.2 | 0.594 (0.497, 0.676) | 8 | 58.9 |
| Vitamin E | 0.618 (0.539, 0.686) | 26 | 92.8 | 0.595 (0.483, 0.688) | 12 | 88.9 | 0.632 (0.583, 0.676) | 26 | 86.9 | 0.521 (0.452, 0.583) | 18 | 78.8 |
| Vitamin K | 0.584 (0.459, 0.686) | 4 | 76.1 | 0.625 (0.515, 0.714) | 2 | 0 | 0.648 (0.473, 0.774) | 3 | 21.9 | 0.697 (0.471, 0.836) | 3 | 52.5 |
| Thiamin | 0.644 (0.603, 0.682) | 26 | 75.5 | 0.606 (0.547, 0.660) | 16 | 76.6 | 0.574 (0.537, 0.608) | 29 | 70.8 | 0.455 (0.403, 0.505) | 23 | 63.6 |
| Riboflavin | 0.677 (0.629, 0.721) | 24 | 83.9 | 0.653 (0.580, 0.716) | 13 | 83.6 | 0.611 (0.573, 0.646) | 30 | 77.9 | 0.532 (0.472, 0.588) | 22 | 79.5 |
| Niacin | 0.659 (0.555, 0.742) | 14 | 91.6 | 0.601 (0.506, 0.681) | 11 | 81.7 | 0.633 (0.532, 0.717) | 25 | 94.8 | 0.473 (0.393, 0.546) | 23 | 85.7 |
| Vitamin B6 | 0.664 (0.609, 0.711) | 12 | 50.2 | 0.612 (0.521, 0.689) | 7 | 66.1 | 0.524 (0.434, 0.603) | 19 | 82.8 | 0.519 (0.425, 0.603) | 12 | 77.2 |
| Folate | 0.638 (0.583, 0.686) | 26 | 85 | 0.675 (0.612, 0.728) | 12 | 75.7 | 0.564 (0.513, 0.612) | 27 | 78.4 | 0.533 (0.450, 0.607) | 14 | 73.7 |
| Vitamin B12 | 0.670 (0.594, 0.734) | 14 | 83.4 | 0.563 (0.421, 0.678) | 9 | 91.1 | 0.561 (0.481, 0.632) | 18 | 76 | 0.580 (0.474, 0.669) | 12 | 81.4 |
| Carotene | 0.713 (0.641, 0.773) | 7 | 89.1 | 0.610 (0.477, 0.715) | 7 | 91.8 | 0.554 (0.492, 0.611) | 18 | 88.2 | 0.451 (0.363, 0.532) | 14 | 84.9 |
| β-Carotene | 0.633 (0.572, 0.688) | 17 | 70.9 | 0.618 (0.544, 0.683) | 10 | 59.9 | 0.597 (0.544, 0.644) | 22 | 72.6 | 0.518 (0.476, 0.558) | 18 | 34.5 |
| Se | 0.678 (0.566, 0.765) | 7 | 89.5 | 0.668 (0.298, 0.864) | 3 | 94.6 | 0.618 (0.539, 0.685) | 8 | 67.3 | 0.521 (0.415, 0.612) | 8 | 75.2 |
| Mg | 0.687 (0.597, 0.759) | 17 | 92.5 | 0.630 (0.504, 0.731) | 11 | 90.9 | 0.597 (0.496, 0.682) | 17 | 82.5 | 0.625 (0.512, 0.717) | 8 | 73.9 |
| Ca | 0.645 (0.602, 0.685) | 38 | 83.4 | 0.597 (0.529, 0.658) | 22 | 85.1 | 0.592 (0.553, 0.627) | 53 | 83.3 | 0.579 (0.524, 0.628) | 33 | 83.2 |
| Fe | 0.639 (0.592, 0.682) | 33 | 83.5 | 0.626 (0.562, 0.682) | 21 | 83.4 | 0.569 (0.524, 0.612) | 46 | 85.2 | 0.519 (0.458, 0.574) | 26 | 78.7 |
| I | N/A | N/A | N/A | N/A | N/A | N/A | N/A | N/A | N/A | N/A | N/A | N/A |
| Zn | 0.662 (0.591, 0.723) | 17 | 88.4 | 0.653 (0.538, 0.745) | 10 | 87.9 | 0.535 (0.437, 0.621) | 9 | 70.2 | 0.510 (0.421, 0.590) | 8 | 58.1 |
| Cu | 0.727 (0.570, 0.833) | 5 | 88.2 | 0.703 (0.568, 0.801) | 5 | 87.3 | 0.838 (0.739, 0.901) | 1 | N/A | 0.827 (0.723, 0.895) | 1 | N/A |
| K | 0.658 (0.584, 0.722) | 19 | 87.9 | 0.667 (0.594, 0.731) | 12 | 78.4 | 0.628 (0.596, 0.658) | 30 | 65 | 0.573 (0.524, 0.617) | 22 | 65.2 |
| P | 0.643 (0.562, 0.712) | 16 | 87.1 | 0.586 (0.438, 0.704) | 10 | 91.5 | 0.607 (0.547, 0.660) | 27 | 81 | 0.577 (0.525, 0.624) | 20 | 65.1 |
| Na | 0.662 (0.604, 0.713) | 17 | 82.1 | 0.631 (0.533, 0.712) | 12 | 88 | 0.594 (0.544, 0.641) | 24 | 77.5 | 0.492 (0.428, 0.551) | 18 | 74.4 |
| Mn | 0.667 (0.590, 0.732) | 3 | 0 | N/A | N/A | N/A | 0.640 (0.531, 0.728) | 2 | 12.5 | N/A | N/A | N/A |

* CI, confidence interval; N/A: not available
