# Supplementary material for: A meta-analysis of the reproducibility of food frequency questionnaires in nutritional epidemiological studies
Source: Int J Behav Nutr Phys Act. 2021 Jan 11;18:12. doi: 10.1186/s12966-020-01078-4 (PMC7802360; doi:10.1186/s12966-020-01078-4)
Supplement: Supplementary file 19 — Additional file 19 Supplemental Table 18. Pooled intraclass correlation coefficient for energy and nutrients stratified by dietary recall interval. [file 12966_2020_1078_MOESM19_ESM.docx]

**Supplemental Table 18. Pooled intraclass correlation coefficient for energy and nutrients stratified by dietary recall interval***

| Nutrient | ≥ 12 months | | | | | | < 12 months | | | | | |
| --- | --- | --- | --- | --- | --- | --- | --- | --- | --- | --- | --- | --- |
|  | Crude | | | Energy-adjusted | | | Crude | | | Energy-adjusted | | |
|  | ICC (95% CI) | N | *I^2^* | ICC (95% CI) | N | *I^2^* | ICC (95% CI) | N | *I^2^* | ICC (95% CI) | N | *I^2^* |
| Energy | 0.678 (0.581, 0.756) | 41 | 97.1 | N/A | N/A | N/A | 0.739 (0.673, 0.793) | 15 | 83.6 | N/A | N/A | N/A |
| Protein | 0.624 (0.574, 0.669) | 44 | 88.2 | 0.621 (0.568, 0.670) | 22 | 78.6 | 0.673 (0.600, 0.734) | 14 | 83.6 | 0.417 (0.320, 0.506) | 3 | 0 |
| Fat | 0.621 (0.573, 0.665) | 35 | 84.8 | 0.594 (0.512, 0.665) | 14 | 86.6 | 0.640 (0.551, 0.715) | 15 | 86.9 | 0.427 (0.259, 0.570) | 5 | 59.5 |
| Plant fat | 0.579 (0.449, 0.684) | 4 | 68.1 | N/A | N/A | N/A | N/A | N/A | N/A | N/A | N/A | N/A |
| Animal fat | N/A | N/A | N/A | N/A | N/A | N/A | N/A | N/A | N/A | N/A | N/A | N/A |
| MUFA | 0.635 (0.591, 0.676) | 30 | 78.5 | 0.647 (0.575, 0.709) | 15 | 84 | 0.619 (0.492, 0.721) | 8 | 75.8 | 0.492 (0.238, 0.684) | 3 | 52.7 |
| PUFA | 0.626 (0.532, 0.705) | 34 | 95.4 | 0.581 (0.491, 0.658) | 15 | 86.8 | 0.666 (0.502, 0.784) | 8 | 87.1 | 0.497 (0.279, 0.666) | 3 | 39.9 |
| n-3 PUFA | N/A | N/A | N/A | N/A | N/A | N/A | N/A | N/A | N/A | N/A | N/A | N/A |
| n-6 PUFA | N/A | N/A | N/A | N/A | N/A | N/A | N/A | N/A | N/A | N/A | N/A | N/A |
| SFA | 0.671 (0.561, 0.757) | 36 | 97.3 | 0.656 (0.582, 0.719) | 16 | 86.2 | 0.694 (0.568, 0.788) | 10 | 84.1 | 0.517 (0.207, 0.732) | 3 | 67.4 |
| Linoleic acid | 0.732 (0.626, 0.812) | 3 | 81.5 | 0.685 (0.591, 0.760) | 3 | 71 | 0.537 (-0.01, 0.839) | 2 | 93.3 | N/A | N/A | N/A |
| Linolenic acid | 0.694 (0.653, 0.730) | 2 | 0 | N/A | N/A | N/A | 0.608 (0.169, 0.846) | 2 | 91.1 | N/A | N/A | N/A |
| EPA | N/A | N/A | N/A | N/A | N/A | N/A | N/A | N/A | N/A | N/A | N/A | N/A |
| DHA | N/A | N/A | N/A | N/A | N/A | N/A | N/A | N/A | N/A | N/A | N/A | N/A |
| Trans-fat | 0.551 (0.398, 0.673) | 3 | 75.1 | N/A | N/A | N/A | 0.769 (0.625, 0.863) | 1 | N/A | N/A | N/A | N/A |
| Cholesterol | 0.660 (0.599, 0.712) | 34 | 90.5 | 0.617 (0.555, 0.672) | 21 | 83 | 0.626 (0.487, 0.734) | 10 | 86.6 | 0.645 (0.397, 0.805) | 4 | 78 |
| Lipid | 0.701 (0.459, 0.846) | 4 | 93.4 | 0.662 (0.370, 0.835) | 4 | 94.5 | N/A |  | N/A | N/A | N/A | N/A |
| Carbohydrate | 0.676 (0.584, 0.751) | 43 | 97 | 0.666 (0.593, 0.728) | 20 | 89.7 | 0.632 (0.535, 0.713) | 14 | 89.1 | 0.446 (0.339, 0.542) | 3 | 15.1 |
| Sucrose | 0.666 (0.575, 0.740) | 3 | 59.6 | N/A | N/A | N/A | 0.529 (0.418, 0.625) | 1 | N/A | N/A | N/A | N/A |
| Sugar | 0.700 (0.593, 0.783) | 3 | 71.4 | N/A | N/A | N/A | 0.730 (0.506, 0.861) | 4 | 89.3 | N/A | N/A | N/A |
| Starch | 0.510 (0.351, 0.640) | 1 | N/A | N/A | N/A | N/A | 0.310 (0.174, 0.434) | 1 | N/A | N/A | N/A | N/A |
| Fiber | 0.658 (0.606, 0.705) | 38 | 89.8 | 0.700 (0.630, 0.759) | 18 | 89.9 | 0.671 (0.584, 0.743) | 11 | 79.7 | 0.428 (0.255, 0.575) | 3 | 61.8 |
| Soluble fiber | N/A | N/A | N/A | N/A | N/A | N/A | N/A | N/A | N/A | N/A | N/A | N/A |
| Insoluble fiber | N/A | N/A | N/A | N/A | N/A | N/A | N/A | N/A | N/A | N/A | N/A | N/A |
| Alcohol | 0.779 (0.694, 0.842) | 14 | 92.7 | 0.803 (0.717, 0.865) | 8 | 89.5 | 0.854 (0.789, 0.900) | 7 | 78.5 | 0.800 (0.707, 0.865) | 1 | N/A |
| Vitamin A | 0.636 (0.534, 0.720) | 18 | 92.6 | 0.611 (0.470, 0.721) | 11 | 92.7 | 0.518 (0.409, 0.612) | 6 | 76 | 0.430 (0.240, 0.588) | 1 | N/A |
| Retinol | 0.599 (0.527, 0.662) | 14 | 70.7 | 0.568 (0.442, 0.673) | 7 | 70.9 | 0.520 (0.334, 0.667) | 3 | 86.8 | 0.414 (0.214, 0.581) | 2 | 52.7 |
| Carotene | 0.586 (0.374, 0.739) | 6 | 93 | 0.512 (0.328, 0.658) | 5 | 86.2 | 0.510 (0.473, 0.544) | 1 | N/A | N/A | N/A | N/A |
| β-Carotene | 0.663 (0.614, 0.707) | 16 | 70.2 | 0.613 (0.456, 0.733) | 6 | 81.9 | 0.739 (0.611, 0.830) | 1 | N/A | N/A | N/A | N/A |
| Vitamin C | 0.667 (0.573, 0.744) | 31 | 95.9 | 0.686 (0.580, 0.769) | 17 | 94.7 | 0.581 (0.486, 0.662) | 11 | 87.3 | 0.398 (0.281, 0.503) | 5 | 53.7 |
| Vitamin D | 0.698 (0.410, 0.859) | 9 | 98.9 | 0.671 (0.391, 0.837) | 5 | 98.1 | 0.560 (0.528, 0.589) | 4 | 0 | N/A | N/A | N/A |
| Vitamin E | 0.683 (0.549, 0.782) | 23 | 97.5 | 0.636 (0.515, 0.732) | 13 | 94.2 | 0.513 (0.418, 0.596) | 7 | 79.9 | 0.353 (0.241, 0.456) | 2 | 0 |
| Vitamin K | 0.710 (0.672, 0.745) | 2 | 0 | N/A | N/A | N/A | 0.590 (0.141, 0.838) | 2 | 92.7 | N/A | N/A | N/A |
| Thiamin | 0.642 (0.578, 0.698) | 20 | 86.9 | 0.636 (0.516, 0.732) | 10 | 93.6 | 0.595 (0.504, 0.673) | 8 | 83.9 | 0.426 (0.320, 0.522) | 2 | 0 |
| Riboflavin | 0.661 (0.587, 0.724) | 16 | 89.3 | 0.660 (0.522, 0.765) | 8 | 94.7 | 0.624 (0.546, 0.692) | 8 | 80.2 | 0.423 (0.317, 0.519) | 2 | 0 |
| Niacin | 0.669 (0.609, 0.722) | 13 | 81.4 | 0.633 (0.516, 0.726) | 8 | 91.7 | 0.619 (0.517, 0.704) | 7 | 84.9 | 0.463 (0.360, 0.555) | 2 | 0 |
| Vitamin B6 | 0.769 (0.508, 0.901) | 8 | 99 | 0.756 (0.559, 0.872) | 4 | 97 | 0.516 (0.406, 0.613) | 3 | 31.1 | 0.459 (0.274, 0.611) | 1 | N/A |
| Folate | 0.596 (0.511, 0.669) | 15 | 86.5 | 0.621 (0.513, 0.709) | 5 | 77.1 | 0.634 (0.539, 0.713) | 7 | 84.4 | 0.450 (0.263, 0.604) | 1 | N/A |
| Vitamin B12 | 0.709 (0.513, 0.835) | 10 | 98.1 | 0.702 (0.501, 0.832) | 6 | 97.2 | 0.524 (0.411, 0.619) | 3 | 32.8 | 0.539 (0.370, 0.674) | 1 | N/A |
| Se | 0.683 (0.626, 0.733) | 8 | 53.5 | 0.586 (0.429, 0.709) | 4 | 78.7 | 0.654 (0.503, 0.767) | 2 | 64.7 | N/A | N/A | N/A |
| Mg | 0.652 (0.574, 0.719) | 11 | 84.2 | 0.617 (0.492, 0.717) | 6 | 87.5 | 0.658 (0.524, 0.760) | 5 | 85.7 | N/A | N/A | N/A |
| Ca | 0.615 (0.564, 0.661) | 39 | 87.2 | 0.661 (0.580, 0.730) | 20 | 91.7 | 0.636 (0.561, 0.701) | 9 | 80.2 | 0.481 (0.390, 0.563) | 3 | 0 |
| Fe | 0.633 (0.584, 0.677) | 28 | 81 | 0.583 (0.504, 0.651) | 15 | 82.2 | 0.588 (0.482, 0.678) | 8 | 87.5 | 0.492 (0.367, 0.599) | 4 | 51 |
| I | N/A | N/A | N/A | N/A | N/A | N/A | N/A | N/A | N/A | N/A | N/A | N/A |
| Zn | 0.601 (0.551, 0.645) | 21 | 69 | 0.592 (0.521, 0.654) | 11 | 63.1 | 0.571 (0.469, 0.659) | 4 | 80.5 | 0.369 (0.232, 0.492) | 1 | N/A |
| Cu | 0.652 (0.611, 0.690) | 3 | 0 | N/A | N/A | N/A | N/A | N/A | N/A | N/A | N/A | N/A |
| K | 0.618 (0.533, 0.690) | 17 | 88.9 | 0.637 (0.486, 0.752) | 7 | 93.6 | 0.692 (0.509, 0.815) | 4 | 89.7 | N/A | N/A | N/A |
| P | 0.605 (0.519, 0.678) | 17 | 86.2 | 0.635 (0.544, 0.711) | 9 | 80.7 | 0.589 (0.398, 0.731) | 5 | 90.9 | N/A | N/A | N/A |
| Na | 0.656 (0.425, 0.807) | 18 | 98.6 | 0.670 (0.474, 0.802) | 8 | 97 | 0.626 (0.512, 0.718) | 5 | 68.2 | N/A | N/A | N/A |
| Mn | N/A | N/A | N/A | N/A | N/A | N/A | N/A | N/A | N/A | N/A | N/A | N/A |

* CI, confidence interval; N/A: not available
