# Supplementary material for: A meta-analysis of the reproducibility of food frequency questionnaires in nutritional epidemiological studies
Source: Int J Behav Nutr Phys Act. 2021 Jan 11;18:12. doi: 10.1186/s12966-020-01078-4 (PMC7802360; doi:10.1186/s12966-020-01078-4)
Supplement: Supplementary file 20 — Additional file 20 Supplemental Table 19. Pooled spearman correlation coefficient for energy and nutrients stratified by dietary recall interval. [file 12966_2020_1078_MOESM20_ESM.docx]

**Supplemental Table 19. Pooled spearman correlation coefficient for energy and nutrients stratified by dietary recall interval ***

| Nutrient | ≥ 12 months | | | | | | < 12 months | | | | | |
| --- | --- | --- | --- | --- | --- | --- | --- | --- | --- | --- | --- | --- |
|  | Crude | | | Energy-adjusted | | | Crude | | | Energy-adjusted | | |
|  | SCC (95% CI) | N | *I^2^* | SCC (95% CI) | N | *I^2^* | SCC (95% CI) | N | *I^2^* | SCC (95% CI) | N | *I^2^* |
| Energy | 0.638 (0.606, 0.667) | 70 | 82.1 | N/A | N/A | N/A | 0.667 (0.617, 0.712) | 26 | 81.3 | N/A | N/A | N/A |
| Protein | 0.605 (0.573, 0.635) | 71 | 80.5 | 0.565 (0.528, 0.600) | 52 | 76.2 | 0.606 (0.556, 0.652) | 24 | 74.5 | 0.546 (0.406, 0.662) | 10 | 91.1 |
| Fat | 0.620 (0.592, 0.646) | 69 | 75.1 | 0.567 (0.524, 0.607) | 48 | 81.5 | 0.616 (0.560, 0.667) | 25 | 81.5 | 0.483 (0.340, 0.605) | 7 | 85 |
| Plant fat | 0.522 (0.434, 0.601) | 4 | 66.3 | N/A | N/A | N/A | 0.679 (0.502, 0.802) | 1 | N/A | N/A | N/A | N/A |
| Animal fat | 0.693 (0.661, 0.722) | 4 | 0 | N/A | N/A | N/A | N/A | N/A | N/A | N/A | N/A | N/A |
| MUFA | 0.616 (0.581, 0.650) | 41 | 97.1 | 0.544 (0.488, 0.595) | 25 | 74 | 0.605 (0.518, 0.678) | 15 | 83.6 | 0.582 (0.406, 0.717) | 7 | 90.9 |
| PUFA | 0.581 (0.545, 0.615) | 42 | 70.9 | 0.513 (0.458, 0.564) | 24 | 73 | 0.645 (0.566, 0.711) | 12 | 78.3 | 0.554 (0.391, 0.683) | 7 | 88.5 |
| n-3 PUFA | 0.657 (0.613, 0.697) | 4 | 0 | 0.477 (0.410, 0.540) | 4 | 21.1 | 0.596 (0.284, 0.793) | 1 | 87.7 | 0.434 (0.161, 0.646) | 1 | 79.1 |
| n-6 PUFA | 0.625 (0.575, 0.670) | 4 | 9.1 | 0.479 (0.413, 0.540) | 4 | 17.8 | 0.502 (0.391, 0.598) | 1 | N/A | 0.293 (0.010, 0.531) | 1 | 77.3 |
| SFA | 0.621 (0.586, 0.653) | 48 | 77.7 | 0.567 (0.516, 0.615) | 30 | 81.2 | 0.630 (0.547, 0.701) | 12 | 79.6 | 0.568 (0.431, 0.679) | 7 | 84.6 |
| Linoleic acid | 0.615 (0.552, 0.670) | 1 | N/A | 0.594 (0.469, 0.694) | 6 | 90.6 | N/A | N/A | N/A | 0.597 (0.526, 0.659) | 2 | 0 |
| Linolenic acid | 0.684 (0.576, 0.769) | 3 | 86.3 | 0.704 (0.273, 0.900) | 2 | 96.8 | N/A | N/A | N/A | 0.579 (0.462, 0.677) | 2 | 59.6 |
| EPA | 0.789 (0.704, 0.852) | 1 | N/A | N/A | N/A | N/A | 0.775 (0.236, 0.949) | 2 | 93.2 | N/A | N/A | N/A |
| DHA | 0.789 (0.704, 0.852) | 1 | N/A | N/A | N/A | N/A | 0.709 (0.378, 0.879) | 2 | 82.1 | N/A | N/A | N/A |
| Trans-fat | 0.616 (0.245, 0.830) | 3 | 96 | N/A | N/A | N/A | 0.524 (0.360, 0.658) | 2 | 0 | N/A | N/A | N/A |
| Cholesterol | 0.611 (0.574, 0.646) | 47 | 81.4 | 0.547 (0.496, 0.594) | 29 | 79.8 | 0.627 (0.532, 0.706) | 13 | 79.9 | 0.619 (0.447, 0.746) | 7 | 89.8 |
| Lipid | 0.581 (0.491, 0.659) | 3 | 0 | 0.459 (0.276, 0.610) | 3 | 63.7 | 0.532 (0.458, 0.599) | 3 | 0 | 0.820 (0.669, 0.905) | 1 | N/A |
| Carbohydrate | 0.636 (0.603, 0.668) | 68 | 84.8 | 0.588 (0.544, 0.629) | 50 | 84.6 | 0.624 (0.558, 0.682) | 23 | 86.1 | 0.586 (0.499, 0.662) | 9 | 78.1 |
| Sucrose | 0.679 (0.601, 0.743) | 5 | 63.3 | N/A | N/A | N/A | N/A | N/A | N/A | N/A | N/A | N/A |
| Sugar | 0.716 (0.656, 0.767) | 5 | 61.7 | 0.706 (0.650, 0.754) | 3 | 19.2 | 0.660 (0.526, 0.762) | 6 | 85 | 0.546 (0.024, 0.834) | 2 | 96.8 |
| Starch | 0.641 (0.604, 0.675) | 4 | 0 | N/A | N/A | N/A | N/A | N/A | N/A | N/A | N/A | N/A |
| Fiber | 0.626 (0.588, 0.662) | 60 | 84.2 | 0.612 (0.569, 0.652) | 44 | 81.4 | 0.648 (0.583, 0.706) | 17 | 74.4 | 0.675 (0.576, 0.753) | 8 | 84.9 |
| Soluble fiber | 0.627 (0.581, 0.669) | 8 | 9.8 | 0.602 (0.552, 0.647) | 7 | 0 | 0.683 (0.436, 0.834) | 4 | 90.2 | 0.582 (0.136, 0.832) | 3 | 93.5 |
| Insoluble fiber | 0.631 (0.584, 0.674) | 8 | 12.6 | 0.624 (0.568, 0.674) | 9 | 47.4 | 0.691 (0.529, 0.804) | 4 | 80.2 | 0.549 (0.209, 0.771) | 3 | 88.8 |
| Alcohol | 0.847 (0.811, 0.877) | 32 | 93.3 | 0.798 (0.753, 0.836) | 22 | 88.2 | 0.835 (0.786, 0.873) | 11 | 80.9 | 0.762 (0.549, 0.881) | 5 | 95.5 |
| Vitamin A | 0.594 (0.534, 0.647) | 26 | 85 | 0.550 (0.459, 0.630) | 17 | 88.8 | 0.679 (0.570, 0.764) | 10 | 92.1 | 0.621 (0.360, 0.792) | 4 | 92.8 |
| Retinol | 0.583 (0.542, 0.619) | 38 | 76.9 | 0.555 (0.506, 0.600) | 30 | 77.4 | 0.513 (0.399, 0.610) | 9 | 89.6 | 0.335 (0.151, 0.497) | 8 | 91.9 |
| Carotene | 0.617 (0.580, 0.651) | 68 | 85 | 0.594 (0.552, 0.632) | 48 | 81 | 0.599 (0.523, 0.665) | 16 | 85.8 | 0.634 (0.527, 0.721) | 8 | 84.9 |
| β-Carotene | 0.615 (0.535, 0.684) | 19 | 83.3 | 0.544 (0.444, 0.629) | 12 | 78.3 | 0.603 (0.507, 0.684) | 8 | 85.8 | 0.624 (0.528, 0.705) | 3 | 47.8 |
| Vitamin E | 0.618 (0.562, 0.669) | 37 | 89.5 | 0.542 (0.477, 0.601) | 27 | 84.5 | 0.652 (0.510, 0.759) | 8 | 95 | 0.716 (0.646, 0.774) | 2 | 27.1 |
| Vitamin K | 0.579 (0.462, 0.675) | 6 | 47.1 | 0.658 (0.553, 0.742) | 5 | 32.7 | 0.660 (0.631, 0.686) | 1 | 0 | N/A | N/A | N/A |
| Thiamin | 0.599 (0.563, 0.634) | 39 | 72.7 | 0.524 (0.473, 0.572) | 30 | 75.9 | 0.611 (0.561, 0.657) | 11 | 62.7 | 0.537 (0.408, 0.645) | 8 | 88 |
| Riboflavin | 0.633 (0.594, 0.670) | 38 | 80.2 | 0.586 (0.542, 0.628) | 26 | 73.3 | 0.640 (0.564, 0.704) | 11 | 85.6 | 0.589 (0.410, 0.724) | 8 | 94.3 |
| Niacin | 0.635 (0.542, 0.713) | 28 | 94.5 | 0.510 (0.435, 0.579) | 26 | 86.9 | 0.605 (0.488, 0.700) | 7 | 83.2 | 0.553 (0.404, 0.674) | 7 | 86.7 |
| Vitamin B6 | 0.605 (0.555, 0.650) | 19 | 64 | 0.529 (0.457, 0.594) | 16 | 73.7 | 0.568 (0.258, 0.772) | 5 | 92.1 | 0.762 (0.624, 0.854) | 2 | 57.6 |
| Folate | 0.608 (0.555, 0.656) | 30 | 78.8 | 0.605 (0.536, 0.665) | 20 | 81.2 | 0.606 (0.525, 0.677) | 13 | 86.7 | 0.634 (0.458, 0.762) | 5 | 87.8 |
| Vitamin B12 | 0.600 (0.544, 0.651) | 20 | 69.1 | 0.530 (0.462, 0.592) | 18 | 70.8 | 0.716 (0.567, 0.819) | 7 | 90.6 | 0.739 (0.516, 0.869) | 3 | 91.8 |
| Carotene | 0.589 (0.517, 0.653) | 20 | 89.8 | 0.501 (0.413, 0.579) | 18 | 89 | 0.677 (0.564, 0.766) | 3 | 92.1 | 0.558 (0.325, 0.727) | 3 | 94.1 |
| β-Carotene | 0.608 (0.566, 0.646) | 29 | 65.7 | 0.565 (0.524, 0.603) | 24 | 49 | 0.494 (0.390, 0.586) | 4 | 38.3 | 0.486 (0.313, 0.629) | 4 | 76.1 |
| Se | 0.641 (0.544, 0.721) | 10 | 84.2 | 0.555 (0.413, 0.670) | 8 | 87.5 | 0.661 (0.527, 0.763) | 4 | 85 | 0.608 (0.260, 0.816) | 3 | 91.9 |
| Mg | 0.651 (0.565, 0.723) | 20 | 87.6 | 0.632 (0.522, 0.722) | 15 | 88.8 | 0.628 (0.501, 0.728) | 8 | 87.7 | 0.614 (0.489, 0.714) | 4 | 74.9 |
| Ca | 0.612 (0.574, 0.647) | 60 | 83.6 | 0.590 (0.543, 0.633) | 43 | 83.8 | 0.623 (0.566, 0.674) | 20 | 79.8 | 0.594 (0.496, 0.678) | 11 | 85.2 |
| Iron | 0.618 (0.579, 0.656) | 49 | 82.2 | 0.583 (0.535, 0.626) | 36 | 79.5 | 0.577 (0.513, 0.636) | 19 | 81.2 | 0.523 (0.385, 0.638) | 10 | 90.1 |
| I | N/A | N/A | N/A | N/A | N/A | N/A | N/A | N/A | N/A | N/A | N/A | N/A |
| Zn | 0.633 (0.564, 0.694) | 19 | 82.1 | 0.562 (0.449, 0.656) | 12 | 85 | 0.586 (0.467, 0.685) | 5 | 84.1 | 0.690 (0.544, 0.795) | 5 | 87.6 |
| Cu | 0.699 (0.487, 0.833) | 4 | 89.6 | 0.765 (0.610, 0.864) | 3 | 82.3 | 0.838 (0.739, 0.901) | 1 | N/A | 0.682 (0.510, 0.801) | 3 | 85.6 |
| K | 0.628 (0.589, 0.664) | 37 | 74.2 | 0.626 (0.585, 0.663) | 28 | 67.6 | 0.596 (0.518, 0.664) | 7 | 62 | 0.536 (0.360, 0.675) | 5 | 86.6 |
| P | 0.602 (0.542, 0.655) | 34 | 84.8 | 0.592 (0.531, 0.646) | 24 | 80.9 | 0.675 (0.603, 0.735) | 7 | 58 | 0.549 (0.359, 0.696) | 5 | 88.8 |
| Na | 0.603 (0.555, 0.648) | 30 | 79.7 | 0.544 (0.466, 0.613) | 23 | 87.3 | 0.678 (0.616, 0.731) | 9 | 73 | 0.602 (0.510, 0.681) | 6 | 77.2 |
| Mn | 0.667 (0.590, 0.732) | 3 | 0 | N/A | N/A | N/A | 0.703 (0.542, 0.814) | 1 | N/A | N/A | N/A | N/A |

* CI, confidence interval; N/A: not available
