# Supplementary material for: A meta-analysis of the reproducibility of food frequency questionnaires in nutritional epidemiological studies
Source: Int J Behav Nutr Phys Act. 2021 Jan 11;18:12. doi: 10.1186/s12966-020-01078-4 (PMC7802360; doi:10.1186/s12966-020-01078-4)
Supplement: Supplementary file 21 — Additional file 21 Supplemental Table 20. Pooled intraclass correlation coefficient for energy and nutrients stratified by administration mode. [file 12966_2020_1078_MOESM21_ESM.docx]

**Supplemental Table 20. Pooled intraclass correlation coefficient for energy and nutrients stratified by administration mode***

| Nutrient | Self-administration | | | | | | Interview-administration | | | | | | Not available | | | | | |
| --- | --- | --- | --- | --- | --- | --- | --- | --- | --- | --- | --- | --- | --- | --- | --- | --- | --- | --- |
|  | Crude | | | Energy-adjusted | | | Crude | | | Energy-adjusted | | | Crude | | | Energy-adjusted | | |
|  | ICC (95% CI) | N | *I^2^* | ICC (95% CI) | N | *I^2^* | ICC (95% CI) | N | *I^2^* | ICC (95% CI) | N | *I^2^* | ICC (95% CI) | N | *I^2^* | ICC (95% CI) | N | *I^2^* |
| Energy | 0.690 (0.632, 0.741) | 25 | 88.9 | N/A | N/A | N/A | 0.737 (0.622, 0.820) | 25 | 98.2 | N/A | N/A | N/A | 0.676 (0.568, 0.760) | 11 | 83.5 | N/A | N/A | N/A |
| Protein | 0.646 (0.594, 0.692) | 29 | 85.7 | 0.588 (0.513, 0.654) | 10 | 48.9 | 0.684 (0.629, 0.732) | 24 | 90.1 | 0.601 (0.520, 0.672) | 13 | 87.4 | 0.551 (0.430, 0.653) | 10 | 82.5 | 0.644 (0.347, 0.823) | 2 | 87.7 |
| Fat | 0.672 (0.608, 0.727) | 22 | 90.5 | 0.598 (0.388, 0.749) | 4 | 87.7 | 0.627 (0.560, 0.686) | 21 | 90.3 | 0.534 (0.424, 0.629) | 11 | 89.3 | 0.612 (0.511, 0.696) | 12 | 79.3 | 0.614 (0.425, 0.751) | 4 | 69.9 |
| Plant fat | 0.530 (0.446, 0.606) | 4 | 0 | N/A | N/A | N/A | 0.699 (0.612, 0.770) | 1 | N/A | N/A | N/A | N/A | N/A | N/A | N/A | N/A | N/A | N/A |
| Animal fat | N/A | N/A | N/A | N/A | N/A | N/A | N/A | N/A | N/A | N/A | N/A | N/A | N/A | N/A | N/A | N/A | N/A | N/A |
| MUFA | 0.623 (0.568, 0.672) | 19 | 82 | 0.647 (0.545, 0.730) | 6 | 57.1 | 0.648 (0.575, 0.712) | 13 | 84.9 | 0.626 (0.507, 0.721) | 8 | 90.9 | 0.672 (0.575, 0.750) | 9 | 72.3 | 0.619 (0.393, 0.775) | 4 | 78.5 |
| PUFA | 0.630 (0.569, 0.683) | 19 | 85.6 | 0.560 (0.398, 0.689) | 6 | 76.2 | 0.652 (0.451, 0.790) | 14 | 97.8 | 0.560 (0.424, 0.672) | 8 | 91.4 | 0.642 (0.534, 0.730) | 12 | 83.7 | 0.619 (0.393, 0.775) | 4 | 78.5 |
| n-3 PUFA | N/A | N/A | N/A | N/A | N/A | N/A | N/A | N/A | N/A | N/A | N/A | N/A | N/A | N/A | N/A | N/A | N/A | N/A |
| n-6 PUFA | N/A | N/A | N/A | N/A | N/A | N/A | N/A | N/A | N/A | N/A | N/A | N/A | N/A | N/A | N/A | N/A | N/A | N/A |
| SFA | 0.679 (0.619, 0.731) | 22 | 88.8 | 0.648 (0.489, 0.765) | 6 | 81 | 0.723 (0.501, 0.856) | 15 | 98.6 | 0.628 (0.513, 0.721) | 9 | 91.2 | 0.640 (0.533, 0.727) | 12 | 83.4 | 0.651 (0.444, 0.792) | 4 | 77.3 |
| Linoleic acid | 0.642 (0.210, 0.864) | 3 | 96.3 | 0.750 (0.673, 0.810) | 1 | N/A | 0.690 (0.594, 0.765) | 2 | 51.1 | 0.635 (0.588, 0.677) | 2 | 0 | N/A | 1 | N/A | N/A | N/A | N/A |
| Linolenic acid | 0.608 (0.169, 0.846) | 2 | 91.1 | N/A | N/A | N/A | 0.694 (0.653, 0.730) | 2 | 0 | N/A | N/A | N/A | N/A | N/A | N/A | N/A | N/A | N/A |
| EPA | N/A | N/A | N/A | N/A | N/A | N/A | N/A | N/A | N/A | N/A | N/A | N/A | N/A | N/A | N/A | N/A | N/A | N/A |
| DHA | N/A | N/A | N/A | N/A | N/A | N/A | N/A | N/A | N/A | N/A | N/A | N/A | N/A | N/A | N/A | N/A | N/A | N/A |
| Trans-fat | 0.706 (0.596, 0.790) | 2 | 36.6 | N/A | N/A | N/A | 0.502 (0.391, 0.598) | 1 | N/A | N/A | N/A | N/A | 0.439 (0.239, 0.604) | 1 | N/A | N/A | N/A | N/A |
| Cholesterol | 0.666 (0.602, 0.721) | 22 | 89.7 | 0.621 (0.553, 0.679) | 11 | 55.7 | 0.664 (0.563, 0.746) | 16 | 93.7 | 0.603 (0.499, 0.689) | 11 | 90.3 | 0.613 (0.516, 0.694) | 10 | 72.3 | 0.709 (0.458, 0.856) | 3 | 73.6 |
| Lipid | 0.557 (0.331, 0.723) | 2 | 59.5 | 0.590 (0.459, 0.695) | 2 | 0 | 0.800 (0.605, 0.904) | 2 | 92 | 0.720 (0.211, 0.921) | 2 | 97.3 | N/A | N/A | N/A | N/A | N/A | N/A |
| Carbohydrate | 0.687 (0.632, 0.736) | 25 | 89.8 | 0.593 (0.426, 0.721) | 6 | 85.4 | 0.711 (0.577, 0.808) | 26 | 98.4 | 0.658 (0.563, 0.737) | 15 | 92.3 | 0.575 (0.465, 0.668) | 11 | 81.6 | 0.640 (0.443, 0.778) | 2 | 75.3 |
| Sucrose | 0.619 (0.424, 0.759) | 2 | 83.1 | N/A | N/A | N/A | N/A | N/A | N/A | N/A | N/A | N/A | 0.647 (0.489, 0.764) | 2 | 76.7 | N/A | N/A | N/A |
| Sugar | 0.701 (0.532, 0.816) | 5 | 86.5 | N/A | N/A | N/A | 0.730 (0.691, 0.764) | 1 | N/A | N/A | N/A | N/A | 0.717 (0.592, 0.809) | 2 | 34.9 | N/A | N/A | N/A |
| Starch | 0.406 (0.193, 0.584) | 2 | 73.7 | N/A | N/A | N/A | N/A | N/A | N/A | N/A | N/A | N/A | 0.690 (0.536, 0.799) | 1 | N/A | N/A | N/A | N/A |
| Fiber | 0.681 (0.604, 0.745) | 24 | 93.7 | 0.679 (0.505, 0.801) | 7 | 88.8 | 0.710 (0.646, 0.764) | 20 | 91 | 0.657 (0.555, 0.738) | 12 | 92.9 | 0.626 (0.534, 0.702) | 10 | 76.2 | 0.709 (0.486, 0.846) | 2 | 84.3 |
| Soluble fiber | N/A | N/A | N/A | N/A | N/A | N/A | N/A | N/A | N/A | N/A | N/A | N/A | N/A | N/A | N/A | N/A | N/A | N/A |
| Insoluble fiber | N/A | N/A | N/A | N/A | N/A | N/A | N/A | N/A | N/A | N/A | N/A | N/A | N/A | N/A | N/A | N/A | N/A | N/A |
| Alcohol | 0.811 (0.748, 0.859) | 12 | 82.4 | 0.791 (0.721, 0.845) | 5 | 51.2 | 0.780 (0.616, 0.879) | 6 | 95.5 | 0.822 (0.679, 0.904) | 4 | 93.5 | 0.811 (0.691, 0.887) | 4 | 81.5 | N/A | N/A | N/A |
| Vitamin A | 0.653 (0.550, 0.737) | 12 | 93.8 | 0.650 (0.400, 0.811) | 4 | 90.8 | 0.563 (0.468, 0.645) | 10 | 87.1 | 0.535 (0.385, 0.658) | 6 | 87.5 | 0.653 (0.474, 0.781) | 5 | 90.3 | 0.659 (-0.04, 0.926) | 2 | 97.2 |
| Retinol | 0.622 (0.560, 0.677) | 9 | 33.3 | 0.541 (0.403, 0.654) | 6 | 57.4 | 0.555 (0.430, 0.660) | 8 | 90 | 0.537 (0.300, 0.710) | 3 | 88.6 | 0.679 (0.522, 0.792) | 1 | N/A | N/A | 1 | N/A |
| Carotene | 0.675 (0.590, 0.745) | 6 | 87.3 | 0.551 (0.448, 0.639) | 3 | 0 | 0.570 (0.274, 0.767) | 3 | 97.2 | 0.446 (-0.00, 0.744) | 2 | 95.9 | N/A | N/A | N/A | N/A | N/A | N/A |
| β-Carotene | 0.695 (0.623, 0.755) | 12 | 78.3 | 0.544 (0.197, 0.771) | 4 | 85.8 | 0.645 (0.552, 0.722) | 4 | 66.8 | 0.690 (0.594, 0.765) | 2 | 51.1 | 0.647 (0.572, 0.711) | 3 | 0 | N/A | N/A | N/A |
| Vitamin C | 0.686 (0.618, 0.745) | 20 | 91.2 | 0.652 (0.466, 0.782) | 7 | 87.7 | 0.662 (0.527, 0.764) | 19 | 97.7 | 0.673 (0.528, 0.781) | 11 | 96.3 | 0.621 (0.463, 0.742) | 8 | 90.9 | 0.471 (0.140, 0.707) | 4 | 92.9 |
| Vitamin D | 0.645 (0.552, 0.723) | 9 | 92.4 | 0.679 (0.586, 0.755) | 1 | N/A | 0.755 (0.364, 0.919) | 5 | 99.5 | 0.718 (0.300, 0.904) | 2 | 99 | 0.618 (0.517, 0.702) | 2 | 0 | 0.469 (0.311, 0.603) | 1 | N/A |
| Vitamin E | 0.649 (0.586, 0.705) | 13 | 85.9 | 0.664 (0.567, 0.742) | 4 | 52.7 | 0.677 (0.490, 0.805) | 16 | 98.7 | 0.581 (0.391, 0.724) | 9 | 96.5 | 0.682 (0.524, 0.795) | 5 | 86.7 | 0.613 (0.207, 0.838) | 2 | 92.1 |
| Vitamin K | 0.750 (0.596, 0.850) | 1 | N/A | N/A | N/A | N/A | 0.624 (0.346, 0.801) | 3 | 98.1 | N/A | N/A | N/A | N/A | N/A | N/A | N/A | N/A | N/A |
| Thiamin | 0.666 (0.607, 0.718) | 10 | 82.3 | 0.880 (0.839, 0.910) | 1 | N/A | 0.620 (0.556, 0.677) | 16 | 87.7 | 0.550 (0.440, 0.644) | 9 | 89.5 | 0.586 (0.381, 0.737) | 5 | 89 | 0.636 (0.313, 0.827) | 2 | 89.2 |
| Riboflavin | 0.705 (0.632, 0.767) | 10 | 90.7 | 0.859 (0.813, 0.895) | 1 | N/A | 0.671 (0.611, 0.723) | 14 | 87.8 | 0.579 (0.438, 0.692) | 9 | 93.7 | 0.530 (0.238, 0.735) | 4 | 90.6 | N/A | N/A | N/A |
| Niacin | 0.695 (0.623, 0.755) | 9 | 75.6 | 0.739 (0.661, 0.802) | 1 | N/A | 0.667 (0.577, 0.741) | 11 | 92.7 | 0.606 (0.487, 0.703) | 8 | 91.1 | 0.514 (0.416, 0.601) | 2 | 0 | 0.400 (0.231, 0.544) | 1 | N/A |
| Vitamin B6 | 0.681 (0.553, 0.777) | 6 | 87.7 | 0.839 (0.787, 0.880) | 1 | N/A | 0.826 (0.272, 0.968) | 4 | 99.5 | 0.670 (0.406, 0.830) | 4 | 97.1 | 0.600 (0.501, 0.683) | 3 | 44.8 | N/A | N/A | N/A |
| Folate | 0.679 (0.597, 0.746) | 10 | 90.9 | N/A | N/A | N/A | 0.636 (0.558, 0.703) | 11 | 87.7 | 0.597 (0.495, 0.684) | 6 | 76.4 | 0.507 (0.281, 0.680) | 4 | 85 | N/A | N/A | N/A |
| Vitamin B12 | 0.599 (0.509, 0.676) | 6 | 73.2 | 0.690 (0.599, 0.763) | 1 | N/A | 0.742 (0.406, 0.901) | 6 | 98.9 | 0.682 (0.456, 0.825) | 6 | 97.3 | 0.660 (0.552, 0.745) | 1 | N/A | N/A | N/A | N/A |
| Se | 0.658 (0.593, 0.715) | 3 | 0 | N/A | N/A | N/A | 0.668 (0.574, 0.744) | 5 | 77 | 0.539 (0.352, 0.685) | 2 | 65.2 | 0.668 (0.496, 0.790) | 3 | 82.9 | 0.632 (0.294, 0.829) | 2 | 89.8 |
| Mg | 0.710 (0.583, 0.804) | 7 | 89 | 0.679 (0.551, 0.777) | 1 | N/A | 0.635 (0.541, 0.714) | 9 | 89.8 | 0.567 (0.381, 0.708) | 4 | 91.4 | 0.703 (0.606, 0.779) | 3 | 50.9 | 0.739 (0.629, 0.821) | 1 | N/A |
| Ca | 0.629 (0.551, 0.696) | 23 | 92.4 | 0.667 (0.558, 0.754) | 8 | 69.6 | 0.652 (0.590, 0.707) | 22 | 91.2 | 0.627 (0.518, 0.717) | 13 | 94 | 0.588 (0.408, 0.723) | 7 | 89.2 | 0.665 (0.317, 0.855) | 2 | 91.1 |
| Iron | 0.649 (0.564, 0.721) | 17 | 92.6 | 0.540 (0.400, 0.656) | 6 | 59.2 | 0.654 (0.586, 0.713) | 16 | 90.2 | 0.553 (0.473, 0.624) | 11 | 80.8 | 0.560 (0.383, 0.698) | 6 | 86.8 | 0.669 (0.240, 0.879) | 2 | 93.8 |
| I | N/A | N/A | N/A | N/A | N/A | N/A | N/A | N/A | N/A | N/A | N/A | N/A | N/A | N/A | N/A | N/A | N/A | N/A |
| Zn | 0.555 (0.502, 0.604) | 12 | 41 | 0.571 (0.480, 0.651) | 5 | 3.8 | 0.642 (0.582, 0.696) | 11 | 80.8 | 0.593 (0.475, 0.690) | 6 | 84.1 | 0.549 (0.468, 0.622) | 3 | 0 | 0.430 (0.265, 0.570) | 1 | N/A |
| Cu | 0.670 (0.573, 0.748) | 1 | N/A | N/A | N/A | N/A | 0.656 (0.614, 0.694) | 3 | 0 | N/A | N/A | N/A | N/A | N/A | N/A | N/A | N/A | N/A |
| K | 0.749 (0.678, 0.806) | 10 | 90.3 | 0.709 (0.590, 0.799) | 1 | N/A | 0.629 (0.530, 0.711) | 12 | 93 | 0.625 (0.448, 0.754) | 6 | 94.5 | 0.533 (0.363, 0.669) | 3 | 69.4 | N/A | N/A | N/A |
| P | 0.573 (0.519, 0.623) | 8 | 0 | 0.686 (0.596, 0.760) | 4 | 0 | 0.650 (0.527, 0.746) | 12 | 95.3 | 0.606 (0.467, 0.715) | 5 | 89.8 | 0.416 (0.028, 0.694) | 3 | 90.2 | N/A | N/A | N/A |
| N/A | 0.672 (0.596, 0.736) | 5 | 53.8 | N/A | N/A | N/A | 0.683 (0.438, 0.834) | 14 | 99 | 0.684 (0.470, 0.822) | 7 | 97.4 | 0.546 (0.396, 0.669) | 6 | 80.6 | 0.549 (0.406, 0.667) | 1 | N/A |
| Mn | N/A | N/A | N/A | N/A | N/A | N/A | N/A | N/A | N/A | N/A | N/A | N/A | N/A | N/A | N/A | N/A | N/A | N/A |

* CI, confidence interval; N/A: not available
