# Supplementary material for: A meta-analysis of the reproducibility of food frequency questionnaires in nutritional epidemiological studies
Source: Int J Behav Nutr Phys Act. 2021 Jan 11;18:12. doi: 10.1186/s12966-020-01078-4 (PMC7802360; doi:10.1186/s12966-020-01078-4)
Supplement: Supplementary file 22 — Additional file 22 Supplemental Table 21. Pooled spearman correlation coefficient for energy and nutrients stratified by administration mode. [file 12966_2020_1078_MOESM22_ESM.docx]

**Supplemental Table 21. Pooled spearman correlation coefficient for energy and nutrients stratified by administration mode***

| Nutrient | Self-administration | | | | | | Interview-administration | | | | | | Not available | | | | | |
| --- | --- | --- | --- | --- | --- | --- | --- | --- | --- | --- | --- | --- | --- | --- | --- | --- | --- | --- |
|  | Crude | | | Energy-adjusted | | | Crude | | | Energy-adjusted | | | Crude | | | Energy-adjusted | | |
|  | SCC (95% CI) | N | *I^2^* | SCC (95% CI) | N | *I^2^* | SCC (95% CI) | N | *I^2^* | SCC (95% CI) | N | *I^2^* | SCC (95% CI) | N | *I^2^* | SCC (95% CI) | N | *I^2^* |
| Energy | 0.670 (0.637, 0.700) | 58 | 83.6 | N/A | N/A | N/A | 0.644 (0.599, 0.685) | 35 | 86.4 | N/A | N/A | N/A | 0.554 (0.458, 0.637) | 13 | 77.3 | N/A | N/A | N/A |
| Protein | 0.628 (0.598, 0.656) | 56 | 75.2 | 0.578 (0.532, 0.621) | 34 | 77.5 | 0.610 (0.562, 0.652) | 36 | 86.2 | 0.549 (0.483, 0.610) | 23 | 84.2 | 0.513 (0.426, 0.590) | 14 | 72.1 | 0.480 (0.337, 0.600) | 7 | 76.3 |
| Fat | 0.644 (0.612, 0.673) | 57 | 79.8 | 0.568 (0.518, 0.615) | 32 | 79 | 0.610 (0.571, 0.646) | 33 | 78.7 | 0.566 (0.484, 0.638) | 17 | 87 | 0.555 (0.491, 0.613) | 14 | 55.9 | 0.471 (0.360, 0.569) | 7 | 64.4 |
| Plant fat | 0.618 (0.480, 0.726) | 4 | 62.3 | N/A | N/A | N/A | 0.482 (0.430, 0.531) | 2 | 0 | N/A | N/A | N/A | N/A | N/A | N/A | N/A | N/A | N/A |
| Animal fat | 0.735 (0.667, 0.790) | 2 | 0 | N/A | N/A | N/A | 0.683 (0.646, 0.716) | 2 | 0 | N/A | N/A | N/A | N/A | 1 | N/A | N/A | N/A | N/A |
| MUFA | 0.629 (0.596, 0.661) | 25 | 88.9 | 0.556 (0.495, 0.611) | 20 | 76.3 | 0.585 (0.522, 0.642) | 25 | 98.2 | 0.584 (0.434, 0.703) | 8 | 87.6 | 0.584 (0.455, 0.690) | 11 | 83.5 | 0.458 (0.238, 0.633) | 4 | 84.8 |
| PUFA | 0.624 (0.588, 0.656) | 31 | 71.6 | 0.528 (0.462, 0.588) | 18 | 74.2 | 0.552 (0.489, 0.610) | 16 | 75 | 0.542 (0.418, 0.645) | 9 | 87 | 0.562 (0.457, 0.651) | 10 | 77.6 | 0.443 (0.278, 0.583) | 4 | 73.3 |
| n-3 PUFA | 0.618 (0.574, 0.658) | 5 | 52 | 0.477 (0.410, 0.540) | 4 | 21.1 | N/A | N/A | N/A | N/A | N/A | N/A | 0.596 (0.284, 0.793) | 1 | 87.7 | 0.434 (0.161, 0.646) | 1 | 79.1 |
| n-6 PUFA | 0.600 (0.574, 0.625) | 5 | 3 | 0.479 (0.413, 0.540) | 4 | 17.8 | N/A | N/A | N/A | N/A | N/A | N/A | 0.502 (0.391, 0.598) | 1 | N/A | 0.293 (0.010, 0.531) | 1 | 77.3 |
| SFA | 0.644 (0.611, 0.676) | 39 | 78.7 | 0.583 (0.524, 0.636) | 23 | 82.5 | 0.604 (0.552, 0.652) | 17 | 69.8 | 0.564 (0.462, 0.652) | 10 | 83.4 | 0.575 (0.485, 0.653) | 9 | 69.8 | 0.476 (0.331, 0.601) | 4 | 68.5 |
| Linoleic acid | 0.553 (0.503, 0.601) | 6 | 39.4 | 0.493 (0.396, 0.580) | 5 | 71.1 | 0.695 (0.625, 0.754) | 3 | 70.9 | 0.658 (0.543, 0.749) | 4 | 85.3 | N/A | N/A | N/A | N/A | N/A | N/A |
| Linolenic acid | N/A | N/A | N/A | N/A | N/A | N/A | 0.684 (0.576, 0.769) | 3 | 86.3 | 0.642 (0.486, 0.759) | 4 | 91 | N/A | N/A | N/A | N/A | N/A | N/A |
| EPA | 0.891 (0.833, 0.929) | 1 | N/A | N/A | N/A | N/A | 0.700 (0.402, 0.864) | 2 | 80 | N/A | N/A | N/A | N/A | N/A | N/A | N/A | N/A | N/A |
| DHA | 0.806 (0.711, 0.873) | 1 | N/A | N/A | N/A | N/A | 0.700 (0.402, 0.864) | 2 | 80 | N/A | N/A | N/A | N/A | N/A | N/A | N/A | N/A | N/A |
| Trans-fat | 0.696 (0.502, 0.823) | 3 | 85.9 | N/A | N/A | N/A | 0.512 (0.284, 0.685) | 3 | 79.2 | N/A | N/A | N/A | N/A | N/A | N/A | N/A | N/A | N/A |
| Cholesterol | 0.644 (0.615, 0.671) | 37 | 69 | 0.577 (0.529, 0.620) | 21 | 66.5 | 0.594 (0.521, 0.659) | 18 | 85.7 | 0.568 (0.445, 0.670) | 11 | 91.2 | 0.524 (0.404, 0.626) | 11 | 82.2 | 0.401 (0.240, 0.541) | 5 | 73.8 |
| Lipid | 0.613 (0.499, 0.706) | 2 | 0 | 0.526 (0.329, 0.679) | 2 | 53.4 | 0.532 (0.466, 0.592) | 4 | 0 | 0.626 (-0.06, 0.911) | 2 | 93.8 | N/A | N/A | N/A | N/A | N/A | N/A |
| Carbohydrate | 0.672 (0.642, 0.700) | 55 | 80 | 0.599 (0.549, 0.644) | 33 | 80.8 | 0.604 (0.546, 0.657) | 33 | 90.8 | 0.594 (0.516, 0.662) | 19 | 89.3 | 0.550 (0.451, 0.635) | 13 | 79.8 | 0.496 (0.419, 0.567) | 8 | 41.3 |
| Sucrose | 0.711 (0.630, 0.777) | 6 | 72.4 | N/A | N/A | N/A | N/A | N/A | N/A | N/A | N/A | N/A | 0.693 (0.607, 0.762) | 1 | N/A | N/A | N/A | N/A |
| Sugar | 0.688 (0.611, 0.752) | 10 | 81.2 | 0.608 (0.397, 0.757) | 4 | 90.7 | 0.693 (0.642, 0.737) | 1 | N/A | 0.723 (0.677, 0.764) | 1 | N/A | N/A | N/A | N/A | N/A | N/A | N/A |
| Starch | 0.636 (0.530, 0.723) | 2 | 0 | N/A | N/A | N/A | 0.642 (0.602, 0.678) | 2 | 0 | N/A | N/A | N/A | N/A | N/A | N/A | N/A | N/A | N/A |
| Fiber | 0.666 (0.635, 0.695) | 52 | 79.7 | 0.641 (0.594, 0.683) | 32 | 80.7 | 0.618 (0.544, 0.681) | 23 | 89.1 | 0.607 (0.519, 0.683) | 16 | 88.2 | 0.549 (0.447, 0.637) | 12 | 77.8 | 0.522 (0.381, 0.640) | 5 | 69.3 |
| Soluble fiber | 0.693 (0.654, 0.728) | 10 | 56 | 0.602 (0.552, 0.647) | 7 | 0 | 0.671 (0.169, 0.896) | 2 | 90.5 | 0.869 (0.756, 0.932) | 1 | N/A | 0.486 (0.314, 0.626) | 2 | 54.3 | 0.349 (0.050, 0.590) | 2 | 80.5 |
| Insoluble fiber | 0.678 (0.624, 0.725) | 8 | 41.5 | 0.624 (0.568, 0.674) | 9 | 47.4 | 0.597 (0.266, 0.802) | 2 | 77.6 | 0.809 (0.653, 0.900) | 1 | N/A | 0.583 (0.483, 0.666) | 2 | 0 | 0.382 (0.138, 0.583) | 2 | 72.6 |
| Alcohol | 0.874 (0.843, 0.900) | 35 | 93.4 | 0.831 (0.787, 0.866) | 19 | 89.1 | 0.752 (0.697, 0.797) | 9 | 79 | 0.693 (0.642, 0.737) | 6 | 36.4 | 0.789 (0.632, 0.883) | 3 | 83.5 | 0.601 (0.383, 0.756) | 2 | 77.6 |
| Vitamin A | 0.679 (0.622, 0.729) | 20 | 89 | 0.603 (0.510, 0.681) | 10 | 83.8 | 0.570 (0.502, 0.631) | 14 | 83.7 | 0.553 (0.373, 0.692) | 7 | 93.6 | 0.490 (0.379, 0.587) | 8 | 68.7 | 0.434 (0.199, 0.622) | 5 | 86.1 |
| Retinol | 0.612 (0.570, 0.651) | 29 | 72.5 | 0.555 (0.492, 0.613) | 22 | 80.6 | 0.558 (0.498, 0.613) | 15 | 83.5 | 0.481 (0.380, 0.570) | 12 | 87.8 | 0.346 (0.135, 0.527) | 5 | 82 | 0.358 (0.156, 0.532) | 4 | 78.4 |
| Carotene | 0.657 (0.627, 0.685) | 52 | 76.9 | 0.618 (0.571, 0.660) | 35 | 80 | 0.597 (0.539, 0.650) | 30 | 89.3 | 0.591 (0.514, 0.658) | 17 | 86.2 | 0.508 (0.391, 0.609) | 10 | 78.8 | 0.430 (0.217, 0.603) | 5 | 83 |
| β-Carotene | 0.662 (0.632, 0.690) | 17 | 44.3 | 0.577 (0.512, 0.635) | 9 | 56.5 | 0.577 (0.426, 0.695) | 10 | 92.3 | 0.593 (0.338, 0.767) | 5 | 88.6 | 0.492 (0.127, 0.739) | 3 | 89.8 | 0.492 (0.338, 0.621) | 1 | N/A |
| Vitamin E | 0.647 (0.610, 0.682) | 31 | 79.6 | 0.570 (0.499, 0.635) | 17 | 81.5 | 0.634 (0.516, 0.729) | 15 | 96.4 | 0.548 (0.381, 0.680) | 9 | 93.7 | 0.469 (0.263, 0.635) | 6 | 83 | 0.503 (0.412, 0.583) | 4 | 0 |
| Vitamin K | 0.627 (0.537, 0.704) | 3 | 0 | 0.648 (0.560, 0.721) | 3 | 0 | 0.568 (0.369, 0.717) | 4 | 77.1 | 0.692 (0.104, 0.921) | 2 | 76.2 | N/A | N/A | N/A | N/A | N/A | N/A |
| Thiamin | 0.626 (0.586, 0.662) | 26 | 75 | 0.563 (0.505, 0.616) | 21 | 75.1 | 0.599 (0.553, 0.642) | 20 | 77.3 | 0.492 (0.400, 0.576) | 12 | 84.9 | 0.557 (0.463, 0.639) | 9 | 66.5 | 0.426 (0.301, 0.537) | 6 | 61.6 |
| Riboflavin | 0.679 (0.649, 0.706) | 28 | 65 | 0.615 (0.544, 0.678) | 19 | 84.2 | 0.636 (0.586, 0.681) | 18 | 84.4 | 0.573 (0.502, 0.636) | 12 | 82.7 | 0.457 (0.304, 0.587) | 8 | 79.1 | 0.394 (0.143, 0.598) | 4 | 81.8 |
| Niacin | 0.652 (0.596, 0.702) | 19 | 72.8 | 0.496 (0.432, 0.554) | 17 | 62 | 0.695 (0.553, 0.797) | 14 | 97.7 | 0.586 (0.462, 0.689) | 12 | 93.6 | 0.437 (0.273, 0.576) | 6 | 75.3 | 0.372 (0.285, 0.452) | 5 | 0 |
| Vitamin B6 | 0.671 (0.619, 0.718) | 17 | 68.7 | 0.610 (0.550, 0.663) | 14 | 64.2 | 0.483 (0.339, 0.606) | 7 | 77 | 0.359 (0.255, 0.455) | 3 | 0 | 0.486 (0.385, 0.575) | 3 | 20.1 | 0.332 (0.123, 0.513) | 2 | 39.7 |
| Folate | 0.658 (0.626, 0.688) | 29 | 68.5 | 0.619 (0.566, 0.666) | 19 | 72 | 0.556 (0.458, 0.641) | 14 | 88.1 | 0.632 (0.365, 0.803) | 5 | 91.8 | 0.444 (0.267, 0.593) | 6 | 78.6 | 0.304 (0.159, 0.437) | 2 | 0 |
| Vitamin B12 | 0.666 (0.608, 0.717) | 17 | 77.6 | 0.575 (0.473, 0.662) | 14 | 88.7 | 0.572 (0.461, 0.665) | 11 | 78.8 | 0.565 (0.422, 0.680) | 7 | 78.4 | N/A | N/A | N/A | N/A | N/A | N/A |
| Carotene | 0.627 (0.587, 0.664) | 16 | 72.8 | 0.499 (0.405, 0.583) | 13 | 87.5 | 0.603 (0.473, 0.707) | 7 | 95.6 | 0.568 (0.373, 0.716) | 6 | 95.2 | 0.411 (0.226, 0.569) | 2 | 47.7 | 0.381 (0.050, 0.636) | 2 | 81 |
| β-Carotene | 0.646 (0.600, 0.689) | 26 | 70.9 | 0.583 (0.531, 0.631) | 20 | 59 | 0.567 (0.508, 0.621) | 9 | 51.7 | 0.526 (0.479, 0.570) | 6 | 0 | 0.464 (0.333, 0.577) | 4 | 49.9 | 0.388 (0.265, 0.499) | 2 | 0 |
| Se | 0.642 (0.553, 0.717) | 6 | 68.5 | 0.562 (0.410, 0.683) | 5 | 81.9 | 0.686 (0.527, 0.799) | 5 | 92.9 | 0.705 (0.065, 0.934) | 2 | 97.1 | 0.600 (0.501, 0.684) | 4 | 52.6 | 0.486 (0.368, 0.590) | 4 | 53.9 |
| Mg | 0.721 (0.649, 0.780) | 15 | 82.2 | 0.672 (0.611, 0.725) | 10 | 55.6 | 0.599 (0.476, 0.698) | 13 | 91.7 | 0.573 (0.377, 0.719) | 8 | 92.8 | 0.650 (0.551, 0.731) | 2 | 0 | 0.571 (0.413, 0.696) | 1 | N/A |
| Ca | 0.664 (0.636, 0.691) | 47 | 72.9 | 0.608 (0.566, 0.646) | 29 | 71 | 0.590 (0.532, 0.644) | 29 | 88.3 | 0.592 (0.496, 0.674) | 19 | 91.7 | 0.495 (0.401, 0.578) | 11 | 68.3 | 0.481 (0.398, 0.557) | 7 | 40.3 |
| Iron | 0.642 (0.605, 0.677) | 42 | 80.6 | 0.615 (0.566, 0.658) | 24 | 71.9 | 0.597 (0.542, 0.648) | 24 | 85.4 | 0.544 (0.458, 0.621) | 17 | 87.1 | 0.496 (0.393, 0.586) | 9 | 68.8 | 0.444 (0.285, 0.579) | 6 | 79.1 |
| I | N/A | N/A | N/A | N/A | N/A | N/A | N/A | N/A | N/A | N/A | N/A | N/A | N/A | N/A | N/A | N/A | N/A | N/A |
| Zn | 0.638 (0.573, 0.694) | 12 | 66.4 | 0.584 (0.476, 0.675) | 9 | 75.7 | 0.631 (0.512, 0.726) | 9 | 92.2 | 0.669 (0.499, 0.789) | 6 | 92.6 | 0.568 (0.415, 0.691) | 5 | 74.9 | 0.455 (0.355, 0.544) | 3 | 0 |
| Cu | 0.741 (0.599, 0.837) | 4 | 80.3 | 0.742 (0.638, 0.819) | 3 | 51.4 | 0.761 (0.332, 0.929) | 2 | 95.4 | 0.710 (0.479, 0.849) | 3 | 93.6 | N/A | N/A | N/A | N/A | N/A | N/A |
| K | 0.671 (0.633, 0.706) | 24 | 69.3 | 0.628 (0.583, 0.669) | 19 | 53.5 | 0.618 (0.558, 0.672) | 18 | 85.7 | 0.624 (0.559, 0.682) | 12 | 77.2 | 0.546 (0.436, 0.639) | 7 | 64.1 | 0.357 (0.107, 0.565) | 3 | 75.2 |
| P | 0.641 (0.573, 0.700) | 18 | 77.1 | 0.607 (0.549, 0.660) | 14 | 59 | 0.637 (0.563, 0.701) | 18 | 88.5 | 0.597 (0.492, 0.684) | 12 | 89 | 0.517 (0.409, 0.611) | 7 | 64.5 | 0.398 (0.234, 0.540) | 4 | 66.1 |
| N/A | 0.661 (0.626, 0.695) | 19 | 38.8 | 0.583 (0.525, 0.636) | 16 | 64.4 | 0.611 (0.531, 0.679) | 16 | 92 | 0.542 (0.402, 0.657) | 10 | 93.9 | 0.513 (0.409, 0.605) | 6 | 55.6 | 0.470 (0.327, 0.592) | 4 | 60.1 |
| Mn | 0.704 (0.627, 0.767) | 3 | 0 | N/A | N/A | N/A | 0.601 (0.506, 0.682) | 2 | 0 | N/A | N/A | N/A | N/A | 1 | N/A | N/A | N/A | N/A |

* CI, confidence interval; N/A: not available
